# Assessment of rhodolith‐forming species diversity in British Columbia uncovers novel cryptic diversity in the genera *Boreolithothamnion* and *Rhodolithia* gen. nov. (Florideophyceae, Rhodophyta) and the occurrence of hybrid rhodoliths

**DOI:** 10.1111/jpy.70066

**Published:** 2025-08-13

**Authors:** Keelie E. Taylor, Gary W. Saunders

**Affiliations:** ^1^ Department of Biology, Centre for Environmental and Molecular Algal Research University of New Brunswick Fredericton New Brunswick Canada

**Keywords:** British Columbia, DNA barcoding, rhodoliths, SEM, species diversity

## Abstract

Rhodolith collections in British Columbia have historically been limited, and published regional species diversity data are poor. The acquisition of recent collections, notably from rhodolith beds in Haida Gwaii, provided an opportunity to assess diversity in these waters. The DNA barcode markers COI‐5P, *rbc*L‐3P, and *psb*A were used to identify unique genetic groups, which were then placed into a phylogenetic context with other coralline algae and subsequently observed anatomically. These analyses uncovered six rhodolith‐forming species: two known, viz. *Boreolithothamnion phymatodeum* and *Boreolithothamnion soriferum*; a species provisionally called *Boreolithothamnion* sp. 1heterocladum; and three novel species described here, viz. *Boreolithothamion astragaloi* sp. nov., *Boreolithothamnion tanuense* sp. nov., and *Rhodolithia gracilis* gen. et. sp. nov., which comprises three varieties. Of particular interest, sequences of the ITS rDNA region showed the variety *Rhodolithia gracilis* var. *gracilis* × *ramosa* var. nov. to be a hybrid of the other two varieties: *Rhodolithia gracilis* var. *gracilis* var. nov. and *Rhodolithia gracilis* var. *ramosa* var. nov. Although understanding the full extent of BC rhodolith beds will require additional sampling, these findings indicate that rhodoliths are widespread and diverse in British Columbia.

**Abbreviations**
BC: British Columbia
COI‐5P: cytochrome *c* oxidase subunit 1
ITS: internal transcribed spacer (of the nuclear ribosomal cistron)
LSU: (nuclear) large subunit ribosomal (DNA)
PCR: polymerase chain reaction
*psb*A: photosystem II protein D1
*rbc*L: ribulose biphosphate carboxylase large subunit

## INTRODUCTION

**FIGURE 1:**
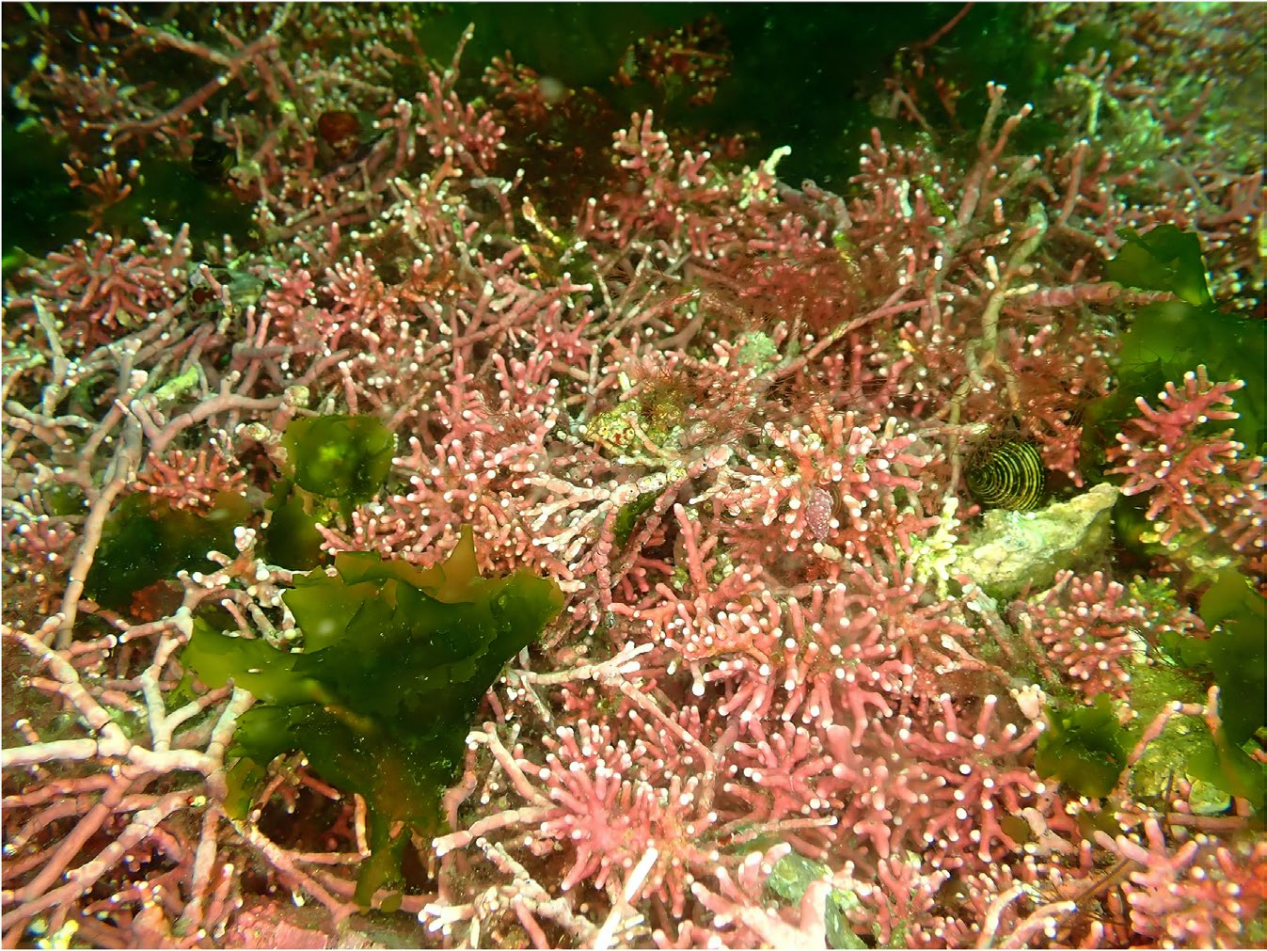
Image of the rhodolith aggregation in the channel between Murchison Island and Faraday Island at Haida Gwaii, BC, from late summer 2022 (Photo credit: G.W. Saunders).

Rhodoliths are a long‐lived but slow‐growing morphology of non‐geniculate coralline algae that live unattached to the substratum (Foster, 2001; Teichert et al., 2014). To be considered a true rhodolith, rather than an algal encrustation or coating, the structure must largely consist of algal biomass (Foster, 2001; Teichert et al., 2014). This definition is particularly applicable to nucleated rhodoliths growing around a core of exogenous material (Freiwald & Henrich, 1994). Nucleated rhodoliths can also be described as autogenic, while non‐nucleated rhodoliths can be described as biogenic (Fredericq et al., 2019). Rhodolith beds (Figure 1) are defined as communities that are dominated by free‐living corallines and are considered one of the four major benthic ecosystems alongside kelp forests, reefs, and seagrass meadows (Foster, 2001).

Rhodoliths are distributed globally and are located throughout the photic zone, typically on soft calcareous sediments (Foster, 2001; Hinojosa‐Arango & Riosmena‐Rodríguez, 2004; Konar et al., 2006; Richards et al., 2022; Robinson et al., 2017). Global rhodolith distribution is shaped by factors such as temperature and light availability, with many species having distinct ranges (Burdett et al., 2012; Fragkopoulou et al., 2021; Pardo et al., 2014). Tropical beds tend to have relatively high rhodolith‐forming species diversity, while polar beds tend to be dominated by a few species (Hinojosa‐Arango & Riosmena‐Rodríguez, 2004; Richards et al., 2022; Teichert et al., 2012; Ward et al., 2021); however, for beds at higher latitudes, notably those in the Northeast Pacific, rhodolith abundance, diversity, and distribution are still comparatively poorly documented (Robinson et al., 2017; Ward et al., 2021). This scarcity of information is particularly noticeable in British Columbia (BC) as there is little published data on the diversity of rhodoliths located there despite reports of rhodoliths existing in the region (British Columbia Marine Conservation Analysis Project Team, 2011; Foster, 2001; Lindstrom et al., 2021; Melbourne et al., 2017; Pardo et al., 2014; Peña et al., 2021; Robinson et al., 2017).

The lack of research in this field can be attributed, in part, to the difficulties of morphological identification owing to the high degree of phenotypic plasticity and evolutionary convergence exhibited by rhodoliths (Basso et al., 2022; Foster et al., 2013; Melbourne et al., 2017; Pardo et al., 2014; Robinson et al., 2017). The gross morphology of rhodoliths is highly variable and environmentally driven, with most collections being categorized into six main growth forms: encrusting, warty, fruticose, lumpy, branched, and foliose (Foster, 2001; Harvey et al., 2016; Melbourne et al., 2017; Sciberras et al., 2009; Teichert et al., 2014). However, growth form is rarely indicative of species, and many rhodolith‐forming species cannot be correctly identified based on morphology alone (Basso et al., 2022; Foster et al., 2013; Fragkopoulou et al., 2021; Melbourne et al., 2017; Richards et al., 2022).

Prior to the widespread use of DNA barcoding, morphological features such as tissue construction, cell shape, cell connections, and reproductive conceptacles were considered diagnostic characters for the identification of non‐geniculate coralline specimens (Johansen, 1981; Woelkerling, 1988). However, many of these characters exhibit overlap among species and even between genera, which frequently makes morphology insufficient for accurate identifications and may result in underestimating the diversity of a population (Richards et al., 2022). In the context of rhodoliths, accurate assessments of diversity are further complicated by variation in rhodolith formation and reproduction among different populations. Current literature has suggested that rhodoliths form from fragmented pieces of existing rhodoliths, from detached nodules of parent crusts, and from spores that settle on and grow around a core material (Foster, 2001; Freiwald & Henrich, 1994; Sciberras et al., 2009). Although rhodoliths with reproductive structures have been documented, they appear to be more common in nucleated or previously nucleated varieties, and conceptacles can become buried or infilled (Konar et al., 2006; Krayesky‐Self et al., 2016; Melbourne et al., 2017; Peña, Rousseau, et al., 2014; Teichert et al., 2012). Conceptacle morphology is often a critical component for alpha taxonomy among the coralline algae (Lanfranco et al., 1999; Woelkerling, 1988), so their absence or obfuscation can further compound the difficulties of rhodolith identification, particularly in novel collections.

Rhodoliths are of high ecological importance because the beds they form promote and support highly diverse benthic ecosystems, primarily through their role as ecosystem engineers (Anderson et al., 2023; Barberá et al., 2003; Foster, 2001; Harvey et al., 2016; Hinojosa‐Arango & Riosmena‐Rodríguez, 2004; Peña, Bárbara, et al., 2014; Steller et al., 2003). Additionally, rhodoliths are key contributors to several biogeochemical cycles such as the global carbon and sulfur cycles (Fredericq et al., 2019; Freiwald & Henrich, 1994; Kamenos et al., 2008; Schubert et al., 2019). From an economic standpoint, rhodolith beds are used as sources of calcium carbonate (Barberá et al., 2003; Foster, 2001), as paleoenvironmental indicators (Basso, 2015; Foster, 2001; Halfar et al., 2000), and as commercial fishing grounds (Barberá et al., 2003). However, as rhodoliths have a slow growth rate, they are considered non‐renewable and are sensitive to both environmental changes and anthropogenic disturbances (Barberá et al., 2003; de Araújo Costa et al., 2023; Foster et al., 2013; Martin & Hall‐Spencer, 2017; Melbourne et al., 2023; Teichert et al., 2014; Vásquez‐Elizondo & Enriquez, 2016). Rhodolith beds are currently listed as threatened and declining habitats (Pardo et al., 2014), and degraded beds have been observed to experience major losses of diversity or changes in community composition (Gabara et al., 2018; Legrand et al., 2024).

Given the importance of rhodoliths both environmentally and economically, preserving their productivity and diversity is critical. However, the lack of data in both the primary literature and herbaria databases on rhodolith diversity in places like BC has hindered conservation efforts. The existing data consist mainly of unidentified specimens or specimens assigned to genetic groups that have yet to be described. Compared to the amount of primary literature and data available on tropical and European beds, rhodolith research in BC is in its infancy. To implement a program for monitoring changes in species composition and preserving ecosystem integrity, it is crucial to have baseline data. Furthermore, making these data accessible in online databases provides a source of information for future studies that may target specific species or focus on ecological interactions within a bed. With this in mind, assessing the species diversity of rhodoliths in BC, and BC's known biodiversity hotspots, is a high priority. This study will be the first of many steps toward preserving the integrity of rhodolith beds in the Northeast Pacific as climate change continues to accelerate.

**FIGURE 2:**
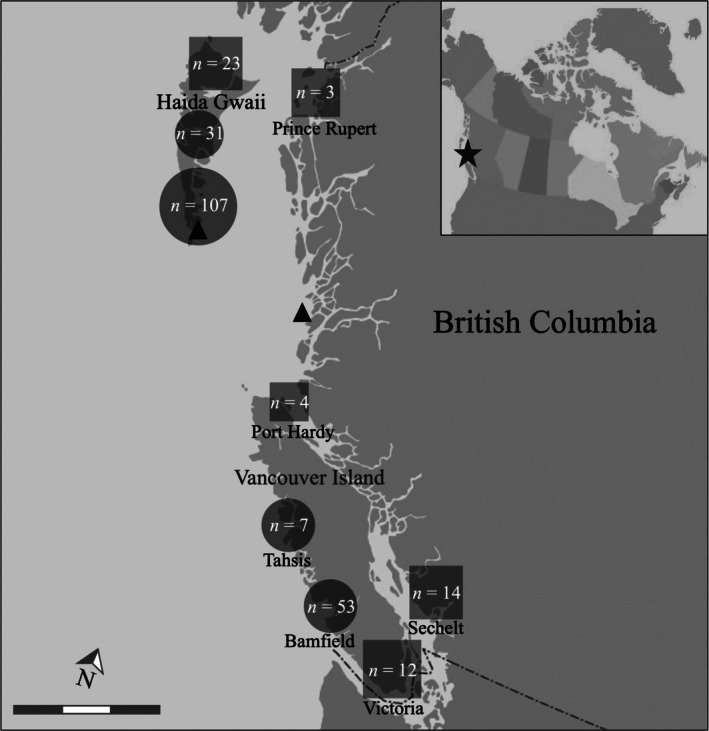
British Columbia barcode survey regions sampled via SCUBA from 1998 to 2023. For surveys conducted by the Saunders lab, squares are regions where rhodoliths were not observed and circles are regions with rhodolith collections, where *n* is the number of dives in each region. Triangles are the collection sites of the Beaty Herbarium rhodolith specimens. Insert is a map of northern North America with the BC coast indicated by a star. (Scale = 200 km; Basemap sourced from Toporama, Natural Resources Canada; contains information licensed under the Open Government License—Canada.)

In 1998, the Saunders lab began a series of DNA barcode surveys in BC that aimed to collect ~5 individuals from every seaweed morphology observed. If rhodoliths were present in the areas being surveyed, representative samples were collected. Particular target regions for the surveys included Bamfield and Tahsis on Vancouver Island, the Sechelt Inlet, Prince Rupert Sound, and the Haida Gwaii archipelago (Figure 2). In 2011, routine surveys at Haida Gwaii, BC, revealed the presence of a large rhodolith bed in the channel between Murchison Island and Faraday Island. This channel was revisited in 2019 and 2021; however, substantial and targeted sampling of rhodolith specimens did not begin until 2022, with the aim of evaluating the species diversity of rhodolith‐forming coralline algae in BC waters. To assess diversity, DNA barcoding data were used to identify unique genetic groups, which were then observed anatomically and placed into a phylogenetic context with other coralline algae. Six non‐geniculate rhodolith‐forming species were uncovered, including four novel species of which three have been described in this study. Additionally, one of these novel species belonged to a new genus and comprised three varieties, one of which was determined to be a hybrid of the other two varieties.

## MATERIALS AND METHODS

### Historical collections

The Connell Memorial Herbarium (UNB; Thiers, 2025) algal database was searched for rhodolith collections that took place during BC barcode surveys prior to the start of this study in 2022. Additionally, rhodolith collections from the Beaty Biodiversity Herbarium (UBC; Thiers, 2025) database (Lipsen & Pitblado, 2024) were similarly searched and documented.

### Field sites and sampling

Rhodolith samples were collected from the subtidal zone via SCUBA during routine barcode surveys of the BC algal flora. The collection metadata for rhodolith (*n* = 299) and crustose (*n* = 10) coralline specimens used in this study are in Table S1. A subsample of each specimen was dried in silica gel for subsequent DNA extraction. Whole specimens were either air dried or dried in vials of silica gel and submitted to the Connell Memorial Herbarium (UNB) as vouchers. Alternatively, photographic e‐vouchers were taken.

### DNA barcoding

DNA was extracted from silica‐dried subsamples following the procedure outlined in Saunders and McDevit (2012). Using methods outlined in Saunders and Moore (2013), polymerase chain reaction (PCR) amplification for the COI‐5P mitochondrial gene marker and the *rbc*L‐3P and *psb*A plastid gene markers was attempted as needed to assign specimens to a genetic group. All PCR products were sent to Genome Quebec for Sanger sequencing. Sequences were aligned and edited in Geneious 2015.9.0. Barcode gap analyses of the COI‐5P, *rbc*L‐3P, and *psb*A sequences were conducted using uncorrected pairwise distances. These analyses determined the unique genetic groups present within our collections by calculating intraspecific variation and the distance to the nearest neighbor. To identify possible hybridization in closely related clusters of these genetic groups, the full nuclear ITS rDNA region was generated as outlined in Saunders and Moore (2013).

### Morphological assessment and microscopy

For brightfield microscopy, small subsamples of each specimen were decalcified in 5% acetic acid (pH 2.4) overnight to prepare each sample for sectioning. For inner anatomical observations, protuberances and branches were sectioned longitudinally, while crustose portions of thalli were sectioned vertically. Sections were made using a Leica CM1850 freezing microtome at 14, 16, 20, and 25 μm thicknesses. Slides were stained with aniline blue and mounted with a solution of 50% Karo syrup and 4% formaldehyde in seawater. A Leica CTR5000 microscope was used to observe both vegetative and reproductive structures, and an OMAX A3514OU microscope‐mounted digital camera was used to capture photomicrographs of features of interest. Cell and tissue measurements were taken in ImageJ (https://imagej.net/ij/).

For scanning electron microscopy, biogenic rhodoliths were fractured both longitudinally and in cross section using a single‐edge razor blade and a hammer to create a flat fracture face. A new razor blade was used for each fracture. Autogenic rhodoliths growing around shells were dipped in liquid nitrogen and fractured vertically using a clean chisel and hammer, while autogenic rhodoliths growing around lithoclastic material were double‐bagged and crushed in a workshop vise to create vertical fractures. This latter technique facilitated observation of the interface between the algal and nucleal material and observation of an intact hypothallium in crustose portions of thalli. Fractured material was mounted on aluminum stubs using forceps and clear glue or adhesive tabs. Stubs were coated in 28 nm of gold and viewed with a JSM 6400 scanning microscope at an accelerating voltage of 15 kV.

### Phylogenetic analyses

In addition to the COI‐5P, *rbc*L‐3P, and *psb*A gene sequences as described above, we generated full‐length sequences for the *rbc*L gene and the nuclear ribosomal LSU rDNA gene (Saunders & Moore, 2013) for both rhodolith and/or crustose representatives of each genetic group identified in the DNA barcode gap analyses, as well as for a range of representative taxa from the Hapalidiales (Table S1). The PCR products were sent to Genome Quebec for Sanger sequencing. From these sequences, four single‐gene alignments were generated: COI‐5P with 48 sequences of 664 bp, *rbc*L with 46 sequences of 1363 bp, *psb*A with 49 sequences of 954 bp, and LSU rDNA with 48 sequences of 1676 bp. These alignments were each analyzed in Geneious 2015.9.0 using maximum likelihood analyses with the GTR + I + G nucleotide substitution model, partitioning by codon for protein coding genes using RAxML and 500 bootstrap replicates to estimate node robustness. A concatenated COI‐5P + *rbc*L + *psb*A + LSU alignment of 49 sequences (4657 bp) was constructed following no detection of conflicts using the same analyses described for the single‐gene alignments, with the addition of partitioning by gene and by codon for the three protein coding genes and with 1000 bootstrap replicates. The resulting phylogenetic tree was rooted on an outgroup of genera from the Corallinales (Nelson et al., 2015; Table S1).

## RESULTS

### Documenting rhodolith collections in British Columbia

Since initiating barcode surveys of the BC algal flora, members of the Saunders lab have conducted approximately 250 SCUBA sampling events across the region (Figure 2). The first rhodolith encounter was in Tahsis in 2008, with three rhodolith specimens collected (Table S1). Dives at Bamfield (Wizard Island) and Haida Gwaii (Hotspring Island, Kwuna Island, Tanu Island, Murchison/Faraday channel, Skidegate channel) between 2010 and 2015 added a further 12 rhodolith specimens to the herbarium collections. In 2019 and 2021, dives revisiting the Murchison/Faraday channel produced three more rhodolith collections. When targeted sampling began in 2022, 128 additional rhodolith samples from the previously sampled Murchison/Faraday site and three more nearby sites (Murchison Island anchorage, Nereo Pinnacle in the Faraday Pass, and a second site in the Murchison/Faraday channel) were added to the collections (Table S1). Divers observed hydrodynamic conditions typical of most rhodolith beds at these sampling sites: shelter from harsh waves but exposure to moderate currents. In 2023 a team of researchers revisited sites at Hotspring Island, Tanu Island, and the Murchison/Faraday channel and additionally visited a new site at Stansung Island. As a result, 153 rhodolith specimens were added to the collections. Review of the Beaty Herbarium database uncovered one rhodolith specimen from Rose Harbour, Haida Gwaii (UBC; A082594), and three rhodolith specimens from Gosling Island off mainland BC (UBC; A092056, A092057, A092058), all of which are currently unidentified (Figure 2).

### Description of novel rhodolith taxa

Herbarium acronyms follow Index Herbariorum online (Thiers, 2025).

#### *Boreolithothamnion astragaloi* K.E.Taylor & G.W.Saunders sp. nov., Figure 3

**FIGURE 3:**
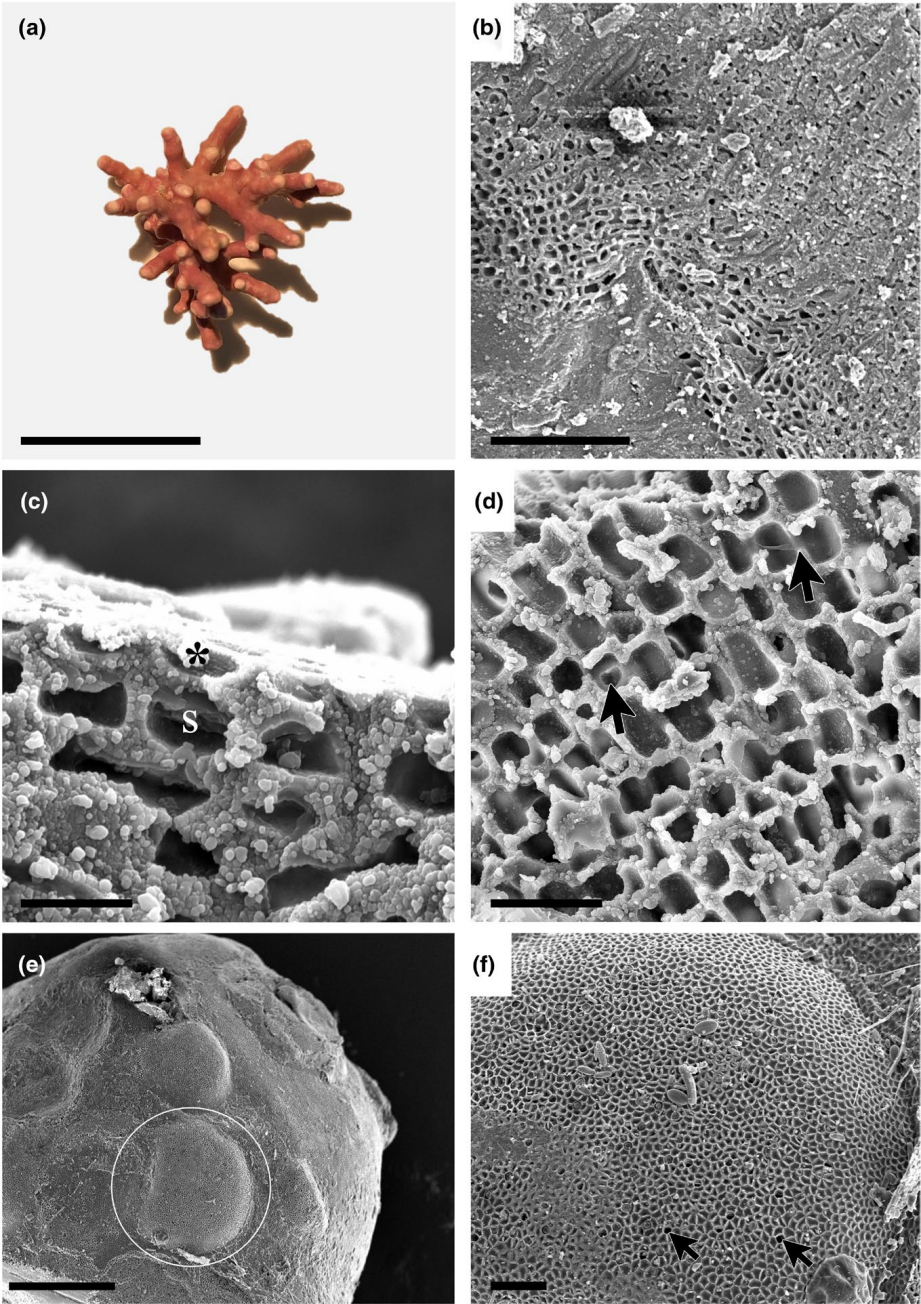
*Boreolithothamnion astragaloi* sp. nov. (a) Non‐nucleated rhodolith specimen in the fruticose growth form (Holotype, GWS048600, scale = 14 mm). (b) Vertical section of the crustose portion of the thallus growing on lithoclastic material showing monomerous, non‐coaxial construction (GWS049844, scale = 100 μm). (c) Longitudinal section of the protuberance showing a flared epithallial cell (*) and subepithallial initial (s) (GWS048600, scale = 10 μm). (d) Longitudinal section of the protuberance showing the presence of abundant cell fusions (arrows) (GWS048600, scale = 20 μm). (e) Surface view of the multiporate sporangial conceptacle (circle) on a protuberance (GWS049821, scale = 400 μm). (f) Magnified view of the multiporate conceptacle showing open pores (arrows) (GWS049821, scale = 50 μm).

**Holotype:** UNB GWS048600, coll. G.W. Saunders & C. Brooks, 2 August 2022, subtidal (5 m)

**Type Locality:** Channel between Murchison Island and Faraday Island, Haida Gwaii, BC, Canada, 52.59687, –131.47513

**Isotypes:** UNB GWS047999, GWS048562, GWS048563, GWS048564, GWS048566, GWS048567, GWS048568, GWS048569, GWS048570, GWS048571, GWS048572, GWS048573, GWS048577, GWS048579, GWS048580, GWS048581, GWS048582, GWS048583, GWS048584, GWS048585, GWS048586, GWS048588, GWS048591, GWS049592, GWS048596, GWS048597, GWS048598, GWS048599, GWS048600, GWS048601, GWS048606, GWS048607, GWS048609, GWS048610, GWS048611, GWS048613, GWS048614, GWS048615, GWS048617, GWS048621, GWS048623, GWS048624, GWS048625

**Description:** Living thalli are reddish‐pink in color. Non‐geniculate crusts with numerous short, knob‐like protuberances, or free‐living rhodoliths in the encrusting, warty, lumpy, and fruticose (Figure 3a) growth forms, both nucleated and non‐nucleated. In crustose portions of thalli, growth is monomerous and plumose/non‐coaxial, with hypothallial filaments growing more or less parallel to the substratum (Figure 3b). In protuberances, growth is monomerous/radial with rhodolith specimens exhibiting growth bands. Hypothallial cells are larger than perithallial cells and rectangular with rounded corners, 10–14 μm in length and 2–6 μm in height. Perithallial cells become smaller toward the epithallus, are ovoid in shape, 6–12 μm in length and 4–7 μm in width. Subepithallial initials are as long as or longer than the subtending perithallial cell. The epithallus consists of a single layer of flared cells (Figure 3c). Secondary pit connections are absent, whereas cell fusions are abundant in both tissues (Figure 3d). Sporangial conceptacles are raised, flat‐topped, and multiporate (Figure 3e); pores are observed both with and without apical plugs (Figure 3f). Gametangial conceptacles were not observed. Differences in the COI‐5P, *rbc*L‐3P, and *psb*A gene sequences are diagnostic for this species. The high degree of morphological plasticity and shared characters exhibited by members of this genus means that specimens cannot be reliably identified by morphology alone. Holotype sequences (GWS048600: PQ882800, *rbc*L‐3P) and representative sequences (GWS046708: PW883042, COI‐5P; PQ888585, *psb*A) can be found on GenBank.

**Etymology:** Named for Astragaloi (Gr., n.) = dice made of sheep knucklebones, wood, or bronze from ancient Greco‐Roman times, modernly used as another name for the game of jacks, which rhodoliths are often compared to in terms of shape

**Distribution:** Skidegate Inlet and northeast Gwaii Hanaas, Haida Gwaii, BC, Canada

**Habit and Habitat:** Subtidal from 5 to 22 m depth, as crusts growing epizoically on shells or as free‐living rhodoliths

#### *Boreolithothamnion tanuense* K.E.Taylor & G.W.Saunders sp. nov., Figure 4

**FIGURE 4:**
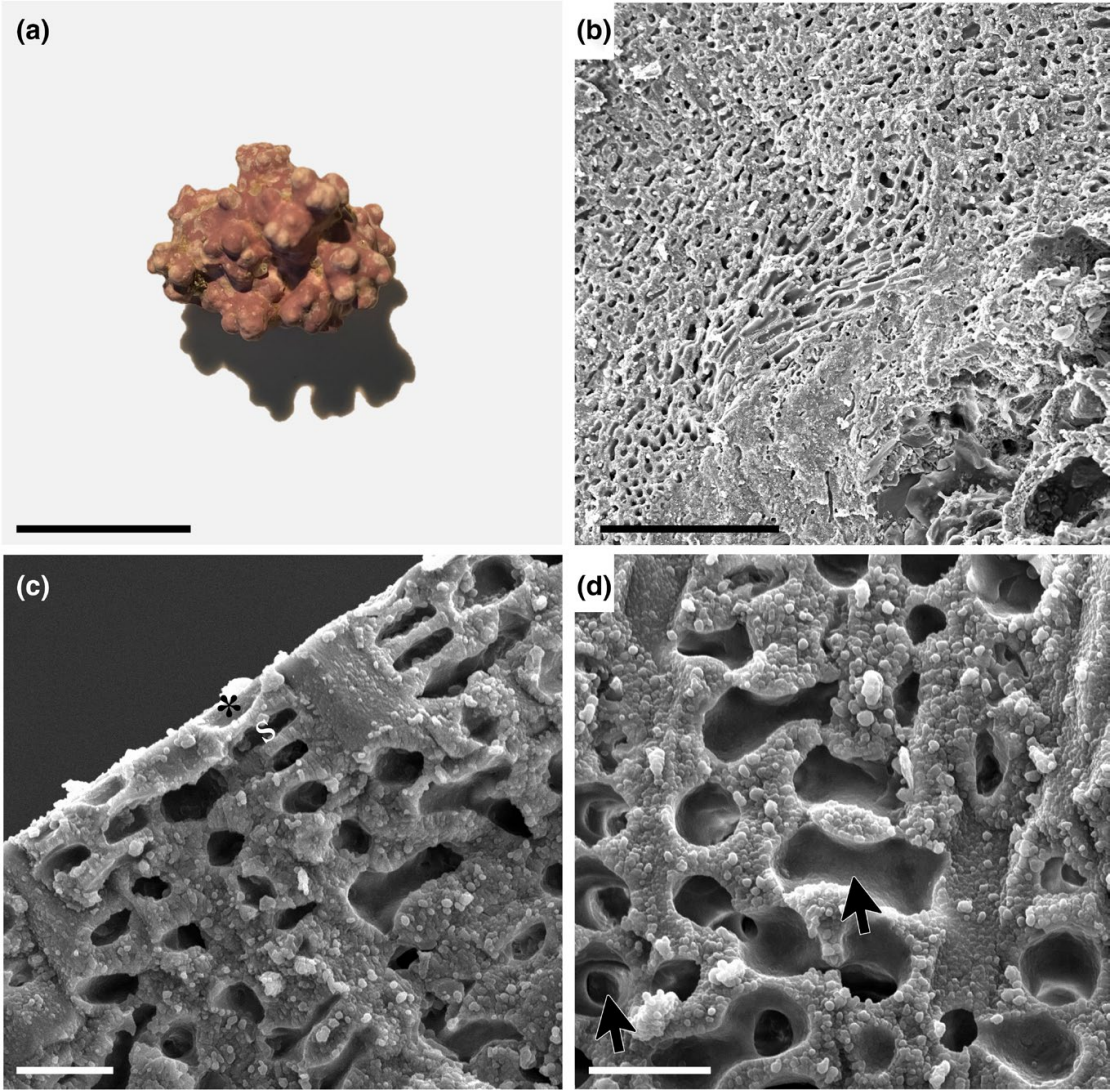
*Boreolithothamnion tanuense* sp. nov. (a) Nucleated rhodolith specimen with numerous warty protuberances (Holotype, GWS049846, scale = 14 mm). (b) Vertical section of crustose portion of thallus growing on lithoclastic material showing monomerous, non‐coaxial construction (GWS049842, scale = 100 μm). (c) Longitudinal section of protuberance showing flared epithallial cell (*) and subepithallial initial (s) (GWS049815, scale = 10 μm). (d) Longitudinal section of protuberance showing the presence of abundant cell fusions (arrows) (GWS049815, scale = 10 μm).

**Holotype:** UNB GWS049846, coll. L. Lee, D. Okamoto, L. Vigneault & A. Galloway, 23 July 2023, subtidal (10.4 m)

**Type Locality:** Tanu Island, Haida Gwaii, BC, Canada, 52.762, –131.618

**Isotypes:** UNB GWS049815, GWS049824, GWS049839, GWS049842, GWS049845, GWS049846, GWS049853

**Description:** Living thalli are reddish‐pink in color. Non‐geniculate crusts with or without short protuberances or free‐living rhodoliths in the warty, lumpy (Figure 4a) and early‐stage fruticose growth forms. All observed rhodoliths are nucleated. In crustose portions of thalli, growth is monomerous and plumose/non‐coaxial, with perithallial filaments arching up from basal hypothallial cells that grow more or less parallel to the substratum (Figure 4b). In protuberances, growth is monomerous/radial. Hypothallial cells are larger than perithallial cells and rectangular with rounded corners, 13–25 μm in length and 3–6 μm in height. Hypothallial cells transition smoothly to perithallial filaments. Perithallial cells become smaller toward the epithallus, are ovoid in shape, 5–9 μm in length and 4–6 μm in width. Subepithallial initials are as long as or longer than the subtending perithallial cells. The epithallus consists of a single layer of flared cells (Figure 4c). Secondary pit connections are absent, whereas cell fusions are abundant in both tissues (Figure 4d). Reproductive specimens were not observed. Differences in the COI‐5P, *rbc*L‐3P, and *psb*A gene sequences are diagnostic for this species. The high degree of morphological plasticity and shared characters exhibited by members of this genus mean that specimens cannot be reliably identified by morphology alone. Holotype sequences (GWS049846: PQ883007, *rbc*L‐3P; PQ888715, *psb*A) and representative sequences (GWS020939: KM254875, COI‐5P) can be found on GenBank.

**Etymology:** Named after the type locality for the species: Tanu Island, Haida Gwaii

**Distribution:** Skidegate Inlet and northeast Gwaii Hanaas, Haida Gwaii, BC, Canada, as well as Pebble Beach, Stillwater Cove, California, United States

**Habit and Habitat:** Subtidal from 4.6 to 11 m depth, as crusts growing epilithically on cobble and pebbles, epizoically on shells, or as free‐living rhodoliths

#### *Rhodolithia* K.E.Taylor & G.W.Saunders gen. nov.

**Generitype species:** *Rhodolithia gracilis* K.E.Taylor & G.W.Saunders sp. nov.

**Description:** Coralline algae growing as non‐geniculate crusts lacking protuberances or as free‐living rhodoliths. Crusts are thin and strongly adherent to the substratum with a slight lighter‐colored margin. Crustose growth is monomerous and plumose/non‐coaxial, with perithallial filaments arching up from basal hypothallial cells that grow more or less parallel to the substratum. Hypothallial cells are larger than perithallial cells and rectangular with rounded corners. Perithallial cells become shorter toward the epithallus and are ovoid in shape. Rhodoliths are of the irregularly branched to fruticose growth form with blunt branch‐tips, and growth is monomerous/radial. Medullary cells are rounded, and ovoid cortical cells become shorter toward the epithallus. In both crusts and rhodoliths, subepithallial initials are typically as short as or shorter than the subtending perithallial cell. The epithallus consists of a single layer of flattened but not flared cells. Secondary pit connections are absent, whereas cell fusions are abundant. Crustose sporangial specimens were observed with flat‐topped, multiporate conceptacles raised above the thallus surface. Apical pore plugs are present. Bisporangia and zonately arranged tetrasporangia were observed. Sporangial features are diagnostic of the family Hapalidiaceae. Gametangial specimens were not observed.

**Etymology:** Refers to the rhodolith morphology formed by varieties of the generitype species

**Comments:** Many vegetative characters are shared with the genera *Phymatolithon* and *Leptophytum* (Table 1), and as the gametangial morphology is currently unknown, distinguishing this genus anatomically is challenging if not impossible.

**TABLE 1:** Morphological and anatomical comparison of vegetative and reproductive characters among the genera *Rhodolithia* gen. nov., *Phymatolithon*, and *Leptophytum*. Morphological data for *Phymatolithon* and *Leptophytum* based on descriptions in Adey (1964), Adey et al. (2001, 2015, 2018) and microscopic analyses conducted by the first author.

Morphological feature	*Rhodolithia*	*Phymatolithon*	*Leptophytum*
**Gross morphology**			
Crustose form	Yes, thin	Yes, thin	Yes, thin
Rhodolith form	Yes, biogenic	Yes, biogenic and autogenic	Yes, autogenic
Protuberances	No	No	No
**Vegetative anatomy**			
Adherence	Strongly adherent	Strongly adherent	More or less adherent
Attachment	Fully attached	Primarily fully attached	Fully attached
Tissue construction	Monomerous, non‐coaxial	Monomerous, non‐coaxial	Monomerous, non‐coaxial
Hypothallial cell shape	Rectangular	Rectangular	Rectangular
Hypothallial cell size	Longer than tall	Longer than tall	Longer than tall
Perithallial cell shape	Ovoid	Squarish to rectangular	Oblong to ovoid
Perithallial cell size	Wider than long	Wide as or wider than long	Longer than wide
Subepithallial initials^a^	Short as or shorter than	Shorter than	Short as or shorter than
Epithallial cell shape	Flattened but not flared	Generally flattened, not flared	Generally flattened, not flared
Epithallial layers	1	0–2	1–3
Trichocytes	Not observed	Absent	Generally absent
1° pit‐plug connections	Present	Present	Present
2° pit‐plug connections	Absent	Absent	Absent
Cell fusions	Present	Present	Present
**Reproductive anatomy**			
Conceptacle primordia	Not observed	Initiated below intercalary meristem (deep)	Initiated below intercalary meristem (shallow)
**Sporangial anatomy**			
Multiporate vs. uniporate	Multiporate	Multiporate	Multiporate
Conceptacle roof shape	Raised, flat‐topped	Raised, generally flat‐topped; or sunken	Primarily raised, flat‐topped to minutely rounded; occasionally sunken
Pore plugs	Present, apical	Present, apical	Present, apical
Rosette cells	6–8	6–8	6–7
Sporangia	Bi‐ and tetrasporangia	Bi‐ and tetrasporangia	Bi‐ and tetrasporangia
Spore arrangement	Zonate	Zonate	Zonate
**Gametangial anatomy**			
Multiporate vs. uniporate	Not observed	Uniporate	Uniporate
Conceptacle roof shape	Not observed	Raised, domed to conical; or weakly sunken	Raised, domed
Spermatangial systems	Not observed	Dendroid on chamber walls	Simple (on floor, walls and roof of chamber); to scarcely dendroid (on floor only)
Gominoblast development	Not observed	Across entire upper region of fusion cell	Absent from periphery of fusion cell

^a^ In comparison to subtending perithallial cell.

#### *Rhodolithia gracilis* K.E.Taylor & G.W.Saunders sp. nov., Figure 5

**FIGURE 5:**
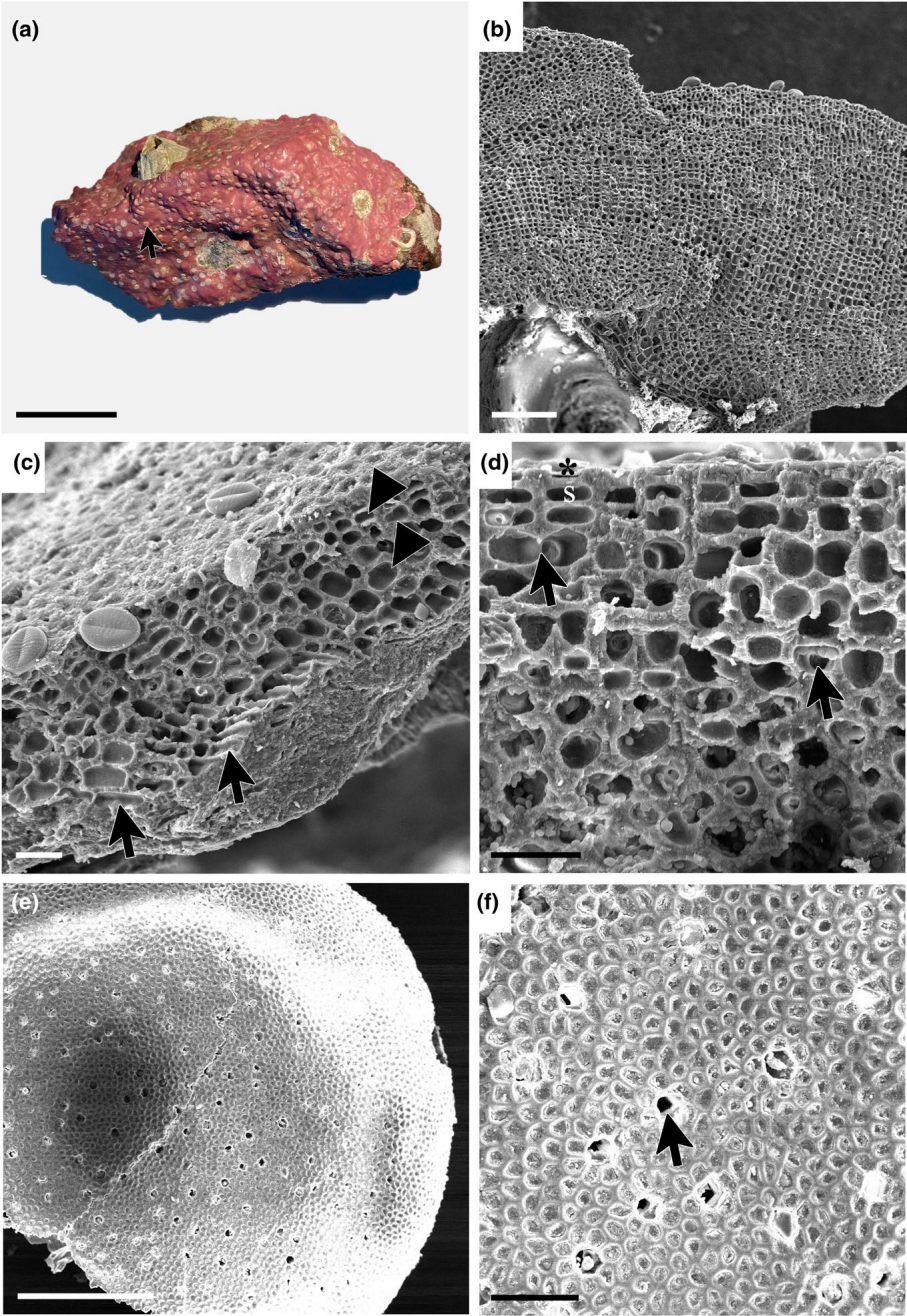
*Rhodolithia gracilis* sp. nov. (Holotype, GWS009188). (a) Crustose specimen growing on cobble with numerous multiporate conceptacles (arrow) (scale = 20 mm). (b) Vertical section of thallus showing thin hypothallus and monomerous construction (scale = 100 μm). (c) Vertical section of thallus showing rectangular hypothallial cells (arrows) and ovoid perithallial cells (arrowheads) that grow smaller toward the thallus surface (scale = 20 μm). (d) Vertical section of thallus showing flattened epithallial cell (*) and subepithallial initial (s) as well as cell fusions (arrows) (scale = 20 μm). (e) Multiporate sporangial conceptacle in surface view (scale = 200 μm). (f) Magnified view of conceptacle surface showing open pore (arrow) (scale = 40 μm).

**Holotype:** UNB GWS009188, coll. G.W. Saunders & B. Clarkston, 22 September 2007, subtidal (10 m)

**Type Locality:** Gilbert Island, Broken Group, Vancouver Island, BC, Canada, 48.879, –125.327

**Description:** Living thalli are bright pink in color and grow as non‐geniculate crusts lacking protuberances (Figure 5a). Growth is monomerous and plumose/non‐coaxial, with perithallial filaments arching up from basal hypothallial cells that grow more or less parallel to the substratum (Figure 5b,c). Hypothallial cells are larger than perithallial cells and rectangular with rounded corners, 9–20 μm in length and 2–5 μm in height (Figure 5c). Hypothallial cells transition smoothly to perithallial filaments. Perithallial cells become shorter toward the epithallus (Figure 5c) and are ovoid in shape, 4–9 μm in length and 5–11 μm in width. Subepithallial initials are typically as short as or shorter than the subtending perithallial cell (Figure 5d). The epithallus consists of a single layer of flattened but not flared cells. Secondary pit connections are absent, whereas cell fusions are abundant. Sporangial specimens with flat‐topped, multiporate conceptacles raised above the thallus surface were observed (Figure 5e). Conceptacle pores were observed both with and without (arrow) apical plugs (Figure 5f). Pores are bordered by six to eight cells. Bisporangia and zonately arranged tetrasporangia were observed. Gametangial specimens were not observed. Differences in the COI‐5P, *rbc*L‐3P, *psb*A gene, and ITS rDNA region sequence are diagnostic for this species. Holotype sequences (GWS009188: PQ883120, ITS; HM918901, COI‐5P; PQ882926, *rbc*L; PQ888593, *psb*A) can be found on GenBank.

**Etymology:** Named for the thin, fragile nature of crustose collections

**Distribution:** Gwaii Hanaas, Haida Gwaii and Bamfield, Vancouver Island, as well as Sechelt Inlet and Howe Sound, BC, Canada

**Habit and Habitat:** Subtidal from 7 to 16 m depth, as crusts growing epilithically on cobble and pebbles

**Comments:** This species comprises three varieties: crust‐forming *Rhodolithia gracilis* var. *gracilis* is the nominate variety, rhodolith‐forming *Rhodolithia gracilis* var. *ramosa* is the second variety, and rhodolith‐forming *Rhodolithia gracilis* var. *gracilis* × *ramosa* is the third variety and a hybrid of the previous two varieties.

#### *Rhodolithia gracilis* var. *ramosa* K.E.Taylor & G.W.Saunders var. nov., Figure 6

**FIGURE 6:**
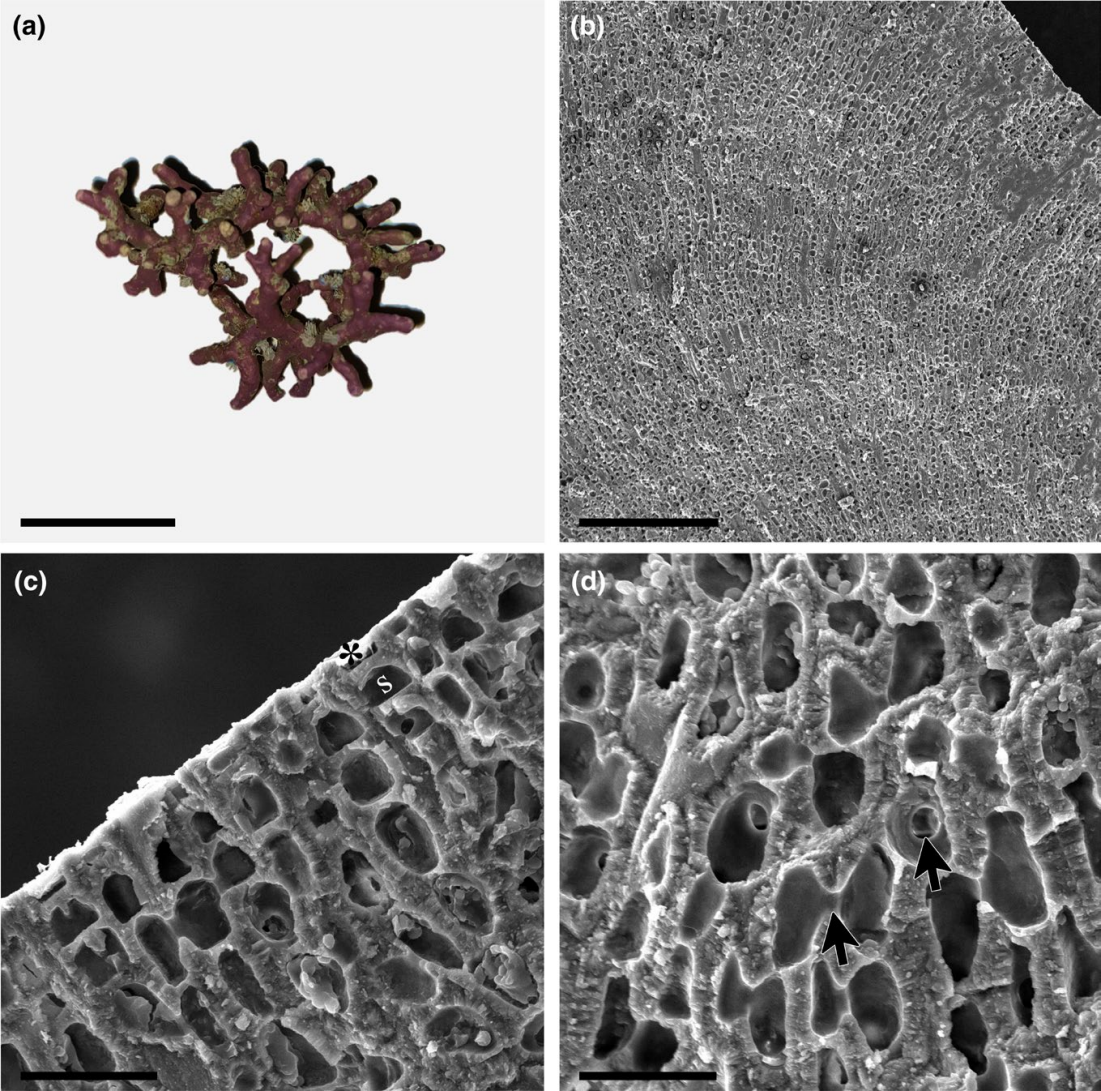
*Rhodolithia gracilis* var. *ramosa* var. nov. (Holotype, GWS010257). (a) Fruticose rhodolith specimen (scale = 20 mm). (b) Longitudinal section of protuberance showing monomerous/radial growth (scale = 200 μm). (c) Longitudinal section of protuberance showing flattened epithallial cell (*) and subepithallial initial (s) (scale = 20 μm). (d) Longitudinal section of protuberance showing cell fusions (arrows) (scale = 20 μm).

**Holotype:** UNB GWS010257, coll. G.W. Saunders & B. Clarkston, 25 May 2008, subtidal (13 m)

**Type Locality:** Tahsis, Princesa Channel, Vancouver Island, BC, Canada, 49.7247, –126.643

**Isotypes:** UNB GWS010269, GWS010270

**Description:** Thalli are dull pink in color and grow as non‐geniculate rhodoliths in the fruticose growth form (Figure 6a). Branches/protuberances growing off the main axis end in rounded but not clubbed tips. Growth is radial/monomerous and plumose/non‐coaxial, with cortical filaments arching out from core medullary tissue (Figure 6b). Medullary cells are rounded, 5–14 μm in length and 5–15 μm in width. Cortical cells are ovoid in shape, 6–13 μm in length and 5–11 μm in width. Subepithallial initials are typically as short as or shorter than the subtending perithallial cell (Figure 6c). The epithallus consists of a single layer of flattened but not flared cells. Secondary pit connections are absent, while cell fusions are abundant throughout (Figure 6d). No reproductive structures were observed. Differences in the ITS rDNA region sequence are diagnostic for this variety. Holotype sequences (GWS010257: PQ883095, ITS; PQ883024, COI‐5P; PQ888458, *psb*A) and representative sequences (GWS010269: PQ883004, *rbc*L‐3P) can be found on GenBank.

**Etymology:** Named for the densely branched nature of fruticose rhodolith collections

**Distribution:** Tahsis, Princesa Channel, Vancouver Island, BC, Canada

**Habit and Habitat:** Subtidal from 8 to 13 m depth, as free‐living rhodoliths

#### *Rhodolithia gracilis* var. *gracilis* × *ramosa* K.E.Taylor & G.W.Saunders var. nov., Figure 7

**FIGURE 7:**
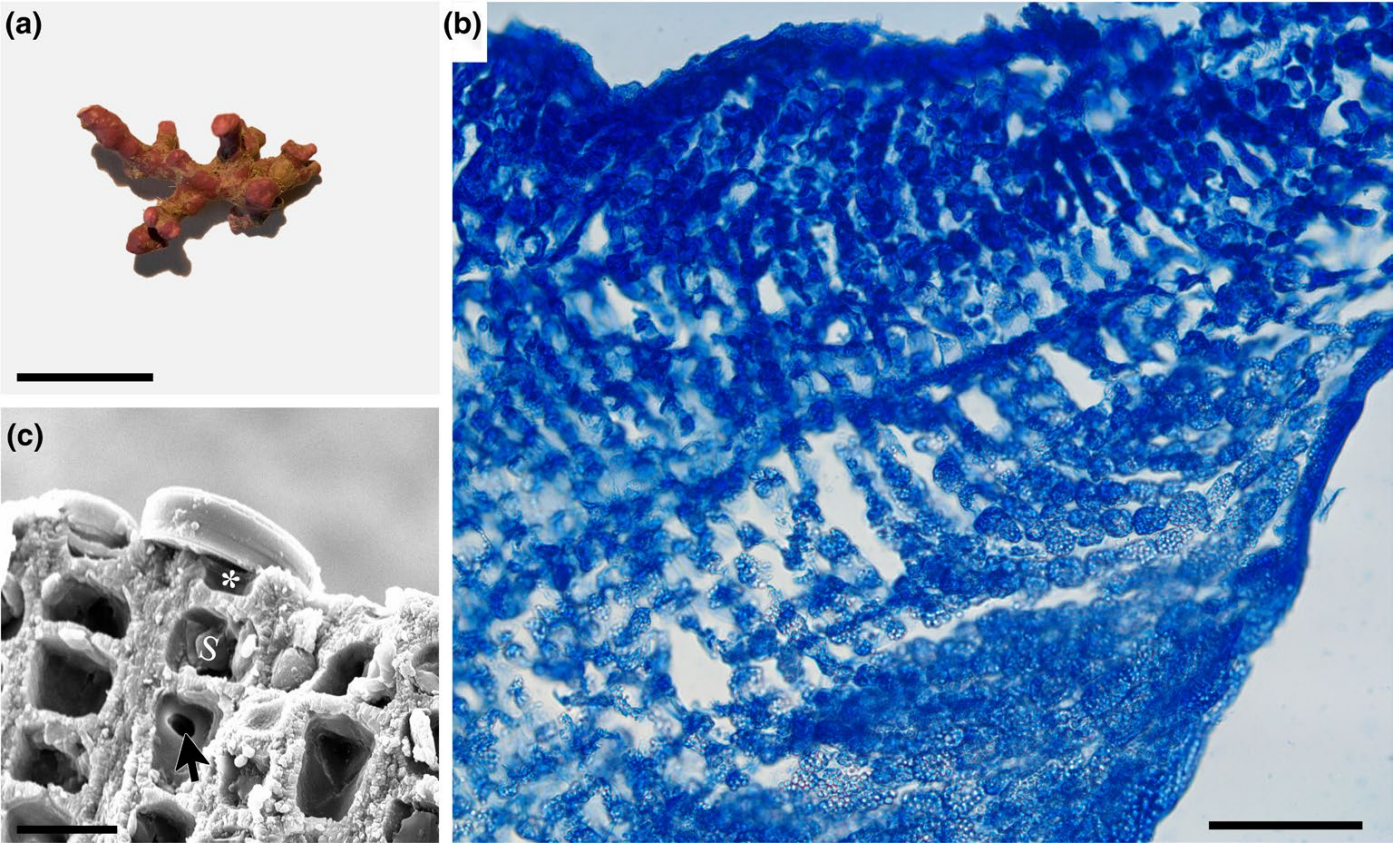
*Rhodolithia gracilis* var. *gracilis* × *ramosa* var. nov. (a) Irregularly branched rhodolith specimen (Holotype, GWS028207, scale = 14 mm). (b) Longitudinal section of protuberance showing monomerous/radial growth (GWS028207, scale = 100 μm). (c) Longitudinal section of protuberance showing flattened epithallial cell (*), subepithallial initial (s) and a cell fusion (arrow) (GWS031003, scale = 10 μm).

**Holotype:** UNB GWS028207, coll. G.W. Saunders and K. Dixon, 7 July 2011, subtidal (22 m)

**Type Locality:** Hotspring Island, Haida Gwaii, BC, Canada, 52.5779, –131.43768

**Description:** Living thalli are reddish‐pink in color and grow as non‐geniculate rhodoliths in an irregularly branched growth form (Figure 7a) Branches/protuberances growing off the main axis are short and end in clubbed tips. Growth is radial/monomerous and plumose/non‐coaxial, with cortical filaments arching out from core medullary tissue (Figure 7b). Medullary cells are rounded, 9–19 μm in length and 6–12 μm in width. Cortical cells become smaller toward the epithallus and are ovoid in shape, 5–11 μm in length and 4–11 μm in width. Subepithallial initials are typically as short as or shorter than the subtending perithallial cell (Figure 7c). The epithallus consists of a single layer of flattened but not flared cells. Secondary pit connections are absent, whereas cell fusions are abundant throughout (Figure 7c). No reproductive specimens were observed. The ITS rDNA region exhibits additivity of both parental ITS types. Medullary cells are somewhat longer in hybrid specimens (9–19 μm) compared to *Rhodolithia gracilis* var. *ramosa* (5–14 μm). However, as these ranges overlap, medullary cell length should be used as a supplementary character to molecular data. Holotype sequences (GWS028207: PQ883127, ITS; PQ883049, COI‐5P; PQ882979, *rbc*L‐3P; PQ888667, *psb*A) can be found on GenBank.

**Etymology:** Hybrid of *Rhodolithia gracilis* var. *gracilis* and *Rhodolithia gracilis* var. *ramosa*

**Distribution:** Northeast Gwaii Haanas, Haida Gwaii, BC, Canada

**Habit and Habitat:** Subtidal from 10 to 22 m depth, as free‐living rhodoliths

**Comments:** In this hybrid variety, COI‐5P, *rbc*L‐3P, and *psb*A gene sequences are of either the *Rhodolithia gracilis* var. *ramosa* or *Rhodolithia gracilis* var. *gracilis* parental type and should not be used as the basis for molecular identification.

### Barcode gap analyses

Of the 299 non‐geniculate BC rhodolith collections used in this study, 266 were successfully barcoded variously using the COI‐5P (*n* = 18), *rbc*L‐3P (*n* = 127) and *psb*A (*n* = 217) genes. Barcode gap analyses of the COI‐5P and *rbc*L‐3P genes revealed six non‐geniculate rhodolith‐forming genetic groups belonging to the genera *Boreolithothamnion* and *Rhodolithia* gen. nov. (Table 2). Two of these genetic groups are currently known species in the BC flora: *Boreolithothamnion soriferum* (*n* = 12) and *Boreolithothamnion phymatodeum* (*n* = 63); one remains undescribed: *Boreolithothamnion* sp. 1heterocladum (*n* = 33); and three are novel species described in this study: *B. astragaloi* sp. nov. (*n* = 135), *B. tanuense* sp. nov. (*n* = 11), and *R. gracilis* sp. nov. (*n* = 22), which comprises three varieties. The nominate variety is crust‐forming *R. gracilis* var. *gracilis* var. nov. (*n* = 10), the second variety is rhodolith‐forming *R. gracilis* var. *ramosa* var. nov. (*n* = 3), and the third, which is a hybrid of the aforementioned varieties, is rhodolith‐forming *R. gracilis* var. *gracilis* × *ramosa* var. nov. (*n* = 9). In all genetic groups, both the maximum intraspecific variation and the distance to the nearest neighbor were higher in the COI‐5P gene than in the *rbc*L‐3P gene (Table 2). Barcode gap analyses with *psb*A gene sequence data did not yield consistent results with the COI‐5P and *rbc*L‐3P genes for *Boreolithothamnion* sp. 1heterocladum, *Boreolithothamnion glaciale*, *B*. sp. 1glaciale, and *B. phymatodeum* (Table S2). There was 0% interspecific distance among *Boreolithothamnion* sp. 1heterocladum, *B. glaciale*, and *Boreolithothamnion* sp. 1glaciale in the *psb*A gene, and for *B. phymatodeum*, intraspecific variation (0.44%) was higher than the distance to its nearest neighbor *B. tanuense* (0.4%). This marker was thus not used in determining the genetic groups present in BC collections, but nonetheless was useful in assigning specimens to established groups with some exceptions (discussed below).

**TABLE 2:** Intraspecific and interspecific variation in the COI‐5P (664 bp) and *rbc*L‐3P (~800 bp) gene regions for rhodolith‐forming and crustose genetic groups of *Boreolithothamnion* and *Rhodolithia* gen. nov., where *n* is the number of sequences.

Species	COI‐5P				*rbc*L‐3P			
	*n*	Max intraspecific variation (%)	Nearest neighbor	Distance to nearest neighbor (%)	*n*	Max intraspecific variation (%)	Nearest neighbor	Distance to nearest neighbor (%)
***Boreolithothamnion***								
*B. astragaloi*	5	0	*B. soriferum*	4.37	77	0	*B. soriferum*	0.88
*B. glaciale*	47	0.9	*B*. sp. 1glaciale	3.46	56	0	*B*. sp. 1glaciale	0.70
*B. phymatodeum*	13	0.56	*B. tanuense*	4.82	30	0.14	*B. tanuense*	1.54
*B. soriferum*	2	0	*B. astragaloi*	4.37	4	0	*B. astragaloi*	0.88
*B*. sp. 1glaciale	2	0	*B. glaciale*	3.46	2	0	*B*. sp. 1heterocladum	0.51
*B*. sp. 1heterocladum	12	0.45	*B*. sp. 1glaciale	3.77	53	0.14	*B*. sp. 1glaciale	0.51
*B. tanuense*	2	0.3	*B. phymatodeum*	4.82	5	0	*B. phymatodeum*	1.54
***Rhodolithia***								
*R. gracilis* var. *gracilis* ^a^	11	0.35	*R. gracilis* var. *ramosa*	2.11	7	0.47	*R. gracilis* var. *ramosa*	0.77
*R. gracilis* var. *ramosa* ^b^	9	0	*R. gracilis* var. *gracilis*	2.11	4	0	*R. gracilis* var. *gracilis*	0.77

^a^ Hybrid specimens with the COI‐5P and *rbc*L‐3P sequences of *R. gracilis* var. *gracilis* were included in these analyses.

^b^ Hybrid specimens with the COI‐5P and *rbc*L‐3P sequences of *R. gracilis* var. *ramosa* were included in these analyses.

**FIGURE 8:**
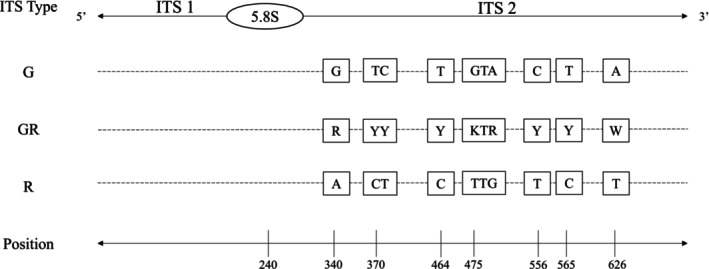
Nucleotide site differences between three ITS rDNA region types (left column), where G is the ITS rDNA region type from *Rhodolithia gracilis* var. *gracilis*, GR is the ITS rDNA region type from *Rhodolithia gracilis* var. *gracilis* × *ramosa*, and R is the ITS rDNA region type from *Rhodolithia gracilis* var. *ramosa*. Boxes represent nucleotide polymorphisms, with the hybrid variety showing additivity of both parent varieties at the nine variable sites.

**FIGURE 9:**
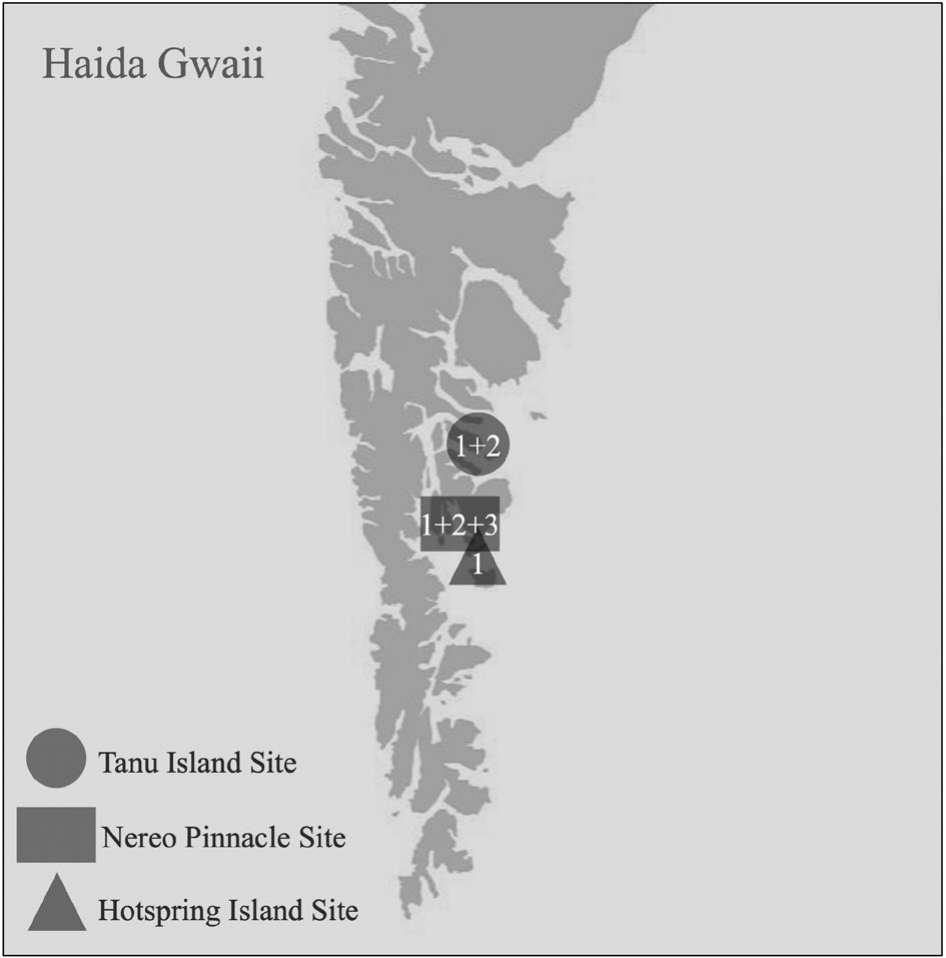
Map of southern Haida Gwaii, BC, showing sites where *Rhodolithia gracilis* var. *gracilis* × *ramosa* specimens were collected and the genetic variant types at each site. Type 1 (*n* = 3) has a mixed ITS rDNA region and mitochondrial and plastid sequences from *R. gracilis* var. *ramosa*. Type 2 (*n* = 3) has a mixed ITS rDNA region, a mitochondrial sequence from *R. gracilis* var. *ramosa*, and a plastid sequence from *R. gracilis* var. *gracilis*. Type 3 (*n* = 1) has a mixed ITS rDNA region, a mitochondrial sequence from *R. gracilis* var. *gracilis*, and a plastid sequence from *R. gracilis* var. *ramosa*. (Basemap sourced from Toporama, Natural Resources Canada; contains information licensed under the Open Government License—Canada.)

*Rhodolithia gracilis* var. *gracilis* × *ramosa* was revealed to be a hybrid of *R. gracilis* var. *gracilis* and *R. gracilis* var. *ramosa* through analysis of the ITS rDNA region. In the hybrid, sequences from the ITS1 rDNA region were of poor quality, while sequences from the ITS2 rDNA region displayed nine sites with double peaks, which suggests contribution from two ITS rDNA types (Figure 8). *Rhodolithia gracilis* var. *gracilis* was determined to be the first contributing parent type, while *R. gracilis* var. *ramosa* was determined to be the second contributing parent type. There were three genetic variants uncovered among the hybrid collections. Type 1 (*n* = 3) had a mixed ITS rDNA region from both parents and mitochondrial and plastid sequences from *R. gracilis* var. *ramosa* (Table 3). This type has been collected at the Tanu, Nereo Pinnacle, and Hotspring, Haida Gwaii, sites (Figure 9). Type 2 (*n* = 3) had a mixed ITS rDNA region from both parents, a mitochondrial sequence from *R. gracilis* var. *ramosa*, and a plastid sequence from *R. gracilis* var. *gracilis*. This type has been collected at the Tanu and Nereo Pinnacle sites. Type 3 (*n* = 1) had a mixed ITS rDNA region from both parents, a mitochondrial sequence from *R. gracilis* var. *gracilis* and a plastid sequence from *R. gracilis* var. *ramosa*; it has only been collected at the Nereo Pinnacle site. For the specimens without COI‐5P gene sequences (GWS046927, GWS046929), a genetic variant type has not been assigned; however, the ITS rDNA region and plastid gene sequences suggest these specimens are either Type 2 or a presumed Type 4, which would have a mixed ITS rDNA region and mitochondrial and plastid sequences from *R. gracilis* var. *gracilis*. *Rhodolithia gracilis* var. *ramosa* has only been collected from Vancouver Island, whereas *R. gracilis* var. *gracilis* has been collected from both Haida Gwaii and Vancouver Island.

**TABLE 3:** ITS, COI‐5P, *rbc*L‐3P, and *psb*A sequence combinations in *Rhodolithia gracilis* var. *gracilis* × *ramosa* specimens. G represents a sequence type from *Rhodolithia gracilis* var. *gracilis*, R represents a sequence type from *Rhodolithia gracilis* var. *ramosa*, and GR represents a sequence type that is a mix of both parent sequences (n.d. means there is no sequence for that marker and specimen). This results in three known hybrid variants: Type 1 (*n* = 3) has a mixed ITS and mitochondria and plastid sequences from *R. gracilis* var. *ramosa*. Type 2 (*n* = 3) has a mixed ITS, a mitochondria sequence from *R. gracilis* var. *ramosa*, and a plastid sequence from *R. gracilis* var. *gracilis*. Type 3 (*n* = 1) has a mixed ITS, a mitochondria sequence from *R. gracilis* var. *gracilis*, and a plastid sequence from *R. gracilis* var. *ramosa*. COI‐5P has not been successfully generated for GWS046927 and GWS046929, so a variant type has not been assigned.

Variety	GWS#	ITS	COI‐5P	*rbc*L‐3P	*psb*A	Variant
*R. gracilis* var. *gracilis* × *ramosa*	GWS028207	GR	R	R	R	1
	GWS031003	GR	R	R	R	1
	GWS046930	GR	R	R	R	1
	GWS031004	GR	R	G	G	2
	GWS046927	GR	n.d.	G	G	—
	GWS046928	GR	R	n.d.	G	2
	GWS046929	GR	n.d.	n.d.	G	—
	GWS046931	GR	R	n.d.	G	2
	GWS046932	GR	G	R	R	3

### Phylogenetic analyses

**FIGURE 10:**
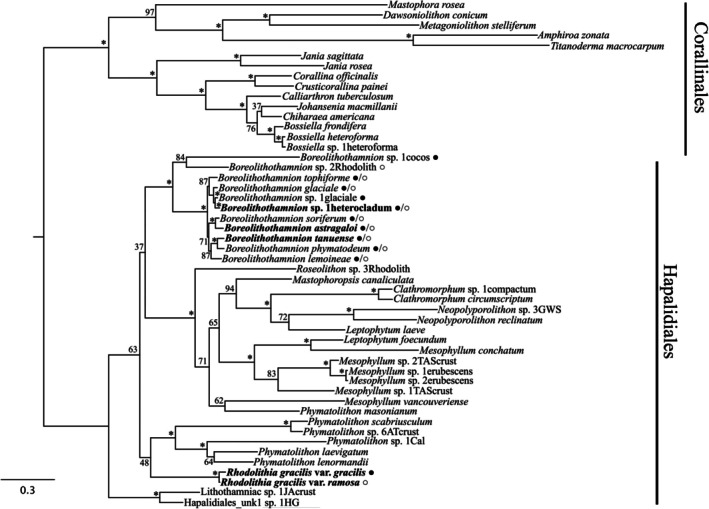
Maximum likelihood (RAxML) tree for the concatenated COI‐5P + *rbc*L + *psb*A + LSU alignment. ML bootstrap support is indicated by the values at each node; an asterisk represents support greater than or equal to 98%. Bolded font indicates a novel species or variety. For species in the *Boreolithothamnion* clade and varieties of the novel genus *Rhodolithia*, ● indicates that the species or variety has been collected in crustose form and ○ indicates that the species or variety has been collected in rhodolith form. Scale bar = substitutions per site.

Analyses of single‐gene alignments returned consistent tree topologies, although support for nodes was variable among the markers as expected. The concatenated COI‐5P + *rbc*L + *psb*A + LSU alignment resulted in the best‐supported phylogeny (Figure 10). Rhodolith‐forming *Boreolithothamnion soriferum* and *B. astragaloi* were sibling species with full bootstrap support. Similarly, *B. phymatodeum* and *B. tanuense* were sibling species with 100% bootstrap support. *Boreolithothamnion* sp. 1heterocladum, however, formed a sibling group with *Boreolithothamnion* sp. 1glaciale, which has only been collected in crustose form (Figure 10). Sibling taxa *R. gracilis* var. *gracilis* and *R. gracilis* var. *ramosa* were assigned to the novel genus, *Rhodolithia*, due to the clade's long branch length and weak association to known genera included in the analyses. *R. gracilis* var. *gracilis* × *ramosa* was not included in the phylogeny, as its sequences were variously from either parent type.

## DISCUSSION

### Diversity, morphology, and distribution of non‐geniculate rhodolith‐forming taxa in BC

Although rhodoliths and the beds they form have a well‐documented global occurrence, the volume of research conducted on these benthic habitats is limited compared with other habitats of similar importance, such as coral reefs and kelp forests (de Araújo Costa et al., 2023; Tuya et al., 2023). Much of the rhodolith research conducted to date has focused on larger tropical beds such as those in Brazil or on the colder‐water European beds such as those around Scotland (Barberá et al., 2003; de Araújo Costa et al., 2023; Horta et al., 2016; Pardo et al., 2014). Many other regions with known rhodolith populations have been far less studied. In the Northeast Pacific, rhodolith research has been limited to a few recently identified beds in Alaska (Konar et al., 2006; Robinson et al., 2017; Ward et al., 2021). In BC, rhodolith research has lagged even farther behind, with only a handful of papers and herbarium specimens indicating the presence of rhodoliths in the region (Foster, 2001; Melbourne et al., 2017; Pardo et al., 2014). This study has provided a substantial assessment of the diversity of rhodolith‐forming species of coralline algae in BC, particularly in the important biodiversity hotspots within the province such as Haida Gwaii. A combination of anatomical and molecular data uncovered six rhodolith‐forming species in BC. *Boreolithothamnion phymatodeum* and *B. soriferum* had both previously been documented in the Northeast Pacific, the former as crusts and the latter as both crusts and rhodoliths (Gabrielson et al., 2023; Peña et al., 2021; Sloan & Bartier, 2000; Ward et al., 2021). The remaining four were novel discoveries in the NE Pacific. *Boreolithothamnion* sp. 1heterocladum requires further study (discussed below), while *B. astragaloi* sp. nov., *B. tanuense* sp. nov., and *R. gracilis* gen. et sp. nov. are the novel taxa described herein. *R. gracilis* comprised one crust‐forming and two rhodolith‐forming varieties. The rhodolith‐forming variety *R. gracilis* var. *gracilis* × *ramosa* was a hybrid of the other two *R. gracilis* varieties.

Many studies to date have highlighted the importance of supplementing morphological observations with molecular data to accurately identify coralline algal species (Adey et al., 2001; Robinson et al., 2017). The extensive anatomical work conducted on the BC rhodolith collections here serves to further highlight how environmentally driven morphological plasticity within a species and the convergence of morphological characters both across and within genera can make identification by morphology alone impossible. On an external basis, growth form is not indicative of species but rather the environmental regime to which the individuals were exposed (Foster, 2001). Most of our collections were biogenic and fruticose to branched, or autogenic and warty to lumpy, with each genetic group exhibiting a variety of growth forms. A study by Harvey et al. (2016) on Australian rhodoliths had similar results, with a single species producing specimens of up to six different growth forms. Observation of the internal anatomy of *Boreolithothamnion* collections here revealed that most vegetative characters were shared across members of the genus, as even hypothallial and perithallial cell size exhibited overlap between species. Identifying diagnosable characters in our collections was further complicated by many of our specimens having inactive conceptacles or lacking them entirely. Biogenic rhodolith collections were not observed with reproductive structures, and while some autogenic collections were observed with conceptacles, the majority were in the sporophyte stage and many conceptacles were empty or broken. Uniporate conceptacles were only observed in two autogenic *Boreolithothamnion* sp. 1heterocladum rhodolith specimens. In some rhodolith‐forming species, such as *Phymatolithon calcareum*, it appears not uncommon for specimens to be collected in the sporophyte stage or even occasionally the gametophyte stage (Konar et al., 2006; Pardo et al., 2019). However, reproductive specimens can be far rarer or entirely absent in other species (Pardo et al., 2017, 2019), which is further evidenced by this study's collections. Asexual species or species with only rare sporophyte collections result in less‐comprehensive alpha taxonomy, which is why our study used anatomical data as a supplement to molecular identifications.

In general, rhodolith beds are composed of two to three species, and it is not uncommon for one species to be dominant (Riosmena‐Rodríguez et al., 2010; Steller et al., 2003). For example, rhodolith‐producing species in the genus *Boreolithothamnion* are commonly dominant in assemblages at higher latitudes (Peña et al., 2021). However, there can be upward of 12 rhodolith‐forming species present in a region (Hernández‐Kantun et al., 2017; Pardo et al., 2014; Richards et al., 2022; Riosmena‐Rodríguez et al., 2010). That said, warmer‐water beds are reported to have greater rhodolith diversity than cold‐water beds; for example, beds in the Gulf of California are composed of six main rhodolith species (Hinojosa‐Arango & Riosmena‐Rodríguez, 2004), and in the Guadeloupe archipelago, a single bed can have as many as eight rhodolith‐forming species (Peña, Bárbara, et al., 2014). In contrast, arctic to subarctic beds have been more commonly reported to have only one to two rhodolith species. For example, rhodolith beds around Svalbard, Norway, have been reported to be primarily composed of biogenic *B. glaciale* rhodoliths with additional small amounts of autogenic *Phymatolithon tenue* (Teichert et al., 2012). In Alaska, the rhodolith beds identified by Konar et al. (2006) and Ward et al. (2021) were both reported to be monospecific (composed of *Phymatolithon calcareum* and *B. soriferum*, respectively). Compared to other beds in the Northeastern Pacific, the rhodolith populations in BC have exhibited much higher diversity, with six rhodolith‐forming species uncovered by our preliminary surveys. In Gwaii Hanaas, Haida Gwaii, where our sampling was the most extensive, the four sampling areas (Murchison/Faraday channel, Hotspring Island, Tanu Island, and Stansung Island) had four or five rhodolith‐forming species present at each site. Considerable molecular work has been conducted on rhodolith specimens from Norway, including Svalbard (Peña et al., 2021); however, for the Alaskan beds, only the Kinzarof Lagoon bed (Ward et al., 2021) has had its monospecific composition assessed by molecular data (Peña et al., 2021). The purported *P. calcareum* bed in Herring Bay was initially only assessed using morphological data, and identifications were based on the species' assumed polymorphic nature and the internal anatomy of Alaskan specimens being roughly similar to Atlantic *P. calcareum* (Konar et al., 2006). Subsequent papers cite these identifications as incorrect (Teichert et al., 2014), and recent molecular work by Peña et al. (2021) determined that at least one *P. calcareum* specimen from Herring Bay, Alaska, was *B. soriferum*. Based on this, it is highly likely that the Herring Bay bed contains greater diversity than previously reported and includes cryptic species that will require molecular analyses for identification. Diversity may even be as high as what was observed here for Haida Gwaii.

Compared to beds in Eastern Canada at similar latitudes, BC also appears to have greater species diversity. In Newfoundland and Labrador, rhodolith beds have been reported to consist mainly of *Boreolithothamnion glaciale* and *B. tophiforme*, with *Leptophytum foecundum* rhodoliths occurring to a lesser extent (Adey & Hayek, 2011; Bélanger & Gagnon, 2023; Hernández‐Kantun et al., 2017). However, similar to the Herring Bay, Alaska, bed, these rhodolith communities also appeared to have been primarily, if not solely, assessed morphologically, so the same issue of possible undocumented cryptic diversity remains for these Northwestern Atlantic beds.

In terms of distribution, both *Boreolithothamnion phymatodeum* and *B. soriferum* have well‐documented distributions in the Northeast Pacific, with the former documented from Calvert Island, BC, south to San Nicholas Island, California, and the latter observed from the Aleutian Islands, Alaska, south to BC, Canada (Gabrielson et al., 2023; Melbourne et al., 2017; Peña et al., 2021; Ward et al., 2021). *Boreolithothamnion phymatodeum* collections from this study have extended the species' known distribution range northward to Gwaii Hanaas, Haida Gwaii, BC, and its depth range down to 20 m subtidal. Additionally, the Haida Gwaii specimens were the first instance of this species being collected in rhodolith form, although these records are all autogenic (Table S1). *Boreolithothamnion soriferum* has been documented in both rhodolith and crustose form across its Pacific range; however, the prior reports of rhodolith collections from BC (Peña et al., 2021) were both UNB herbarium specimens collected from Haida Gwaii in 2010–2011 (Table S1). Collections from this study have extended the Pacific depth range down to 10.4 m subtidal.

The species *Boreolithothamnion* sp. 1heterocladum has a currently documented distribution of Haida Gwaii and Bamfield in BC, Canada, and the Juan de Fuca Strait in Washington, United States. There is some preliminary evidence that *Boreolithothamnion* sp. 1heterocladum may be synonymous with the southern hemisphere taxon *Lithothamnion heterocladum* (Paul Gabrielson, personal communication, September 20, 2024). However, as *L. heterocladum* does not currently have published type sequence data, this synonymy requires further investigation. In the Northeast Pacific, *Boreolithothamnion* sp. 1heterocladum was previously misidentified as *B. glaciale* based on *psb*A gene data, which this study noted does not reliably distinguish among *B. glaciale*, *Boreolithothamnion* sp. 1heterocladum, and *Boreolithothamnion* sp. 1glaciale. Peña et al. (2021) reported one rhodolith and two crustose collections from Washington as *B. glaciale*; however, their *rbc*L‐3P gene sequences matched *Boreolithothamnion* sp. 1heterocladum. Additionally, two crustose collections identified as *B. glaciale* by Peña et al. (2021) from BC and Washington were a match to the *Boreolithothamnion* sp. 1glaciale genetic group based on *rbc*L‐3P gene sequences. Peña et al. (2021) also reported three additional crustose *B. glaciale* specimens from Washington and California; however, these identifications were based solely on *psb*A gene sequence data so could not be confirmed in this study. Similarly, *B. glaciale* collections from BC included in both Hind et al. (2019) and Twist et al. (2023) were identified using the *psb*A gene, meaning they could also have been *B*. sp. 1heterocladum or *B*. sp. 1glaciale. This study's results concerning the *psb*A gene as an accurate barcoding marker are not novel. Bruce and Saunders (2017) observed that *psb*A did not distinguish between the red algal sister taxa *Ptilota filicina* and *Pt. subita* despite the COI‐5P gene identifying them as unique genetic groups. Similar to *B*. sp. 1heterocladum and *B*. sp. 1glaciale having previously been assigned to the Atlantic/Arctic *B. glaciale*, *Pt. filicina*, and *Pt. subita* were previously included in the Atlantic/Arctic *Pt. serrata* in the study by Yang et al. (2004) based on *psb*A gene data. Surveys conducted by the Saunders lab in the Northeast Pacific have not uncovered genuine *B. glaciale* south of Nome, Alaska (Table S1). Both the Saunders lab and Peña et al. (2021) collections of *B. glaciale* exhibited high levels of intraspecific variation in the COI‐5P gene, suggesting further investigation may be warranted to determine any geographic trends in genetic variation and whether collections comprise additional cryptic species.

Both *Boreolithothamnion astragaloi* and *Rhodolithia gracilis* var. *gracilis* × *ramosa* have currently only been collected in Haida Gwaii, and *R. gracilis* var. *ramosa* has only been collected from Tahsis on Vancouver Island. *Boreolithothamnion tanuense*, however, has also been collected in Stillwater Cove, California. It is likely that many of our novel taxa have a larger range than has been currently documented, and they may be located as far south as California, as well as north to Alaska. Many species once thought to be endemic to Haida Gwaii have also been collected in California, particularly those that have been absent in southern BC, making their ranges discontinuous (Saunders, 2014). *Boreolithothamnion tanuense*, in particular, has not been collected in crustose or rhodolith form in southern BC. Although additional sampling is necessary to elucidate the full geographic range of this species, it is possible that, like many algal species, *B. tanuense* was transported via kelp rafts northward along the Davidson Current (Saunders, 2014). There are rhodolith beds adjacent to kelp forests in California (Gabara, 2020); however, it is more likely the crustose form of this species growing epilithically or epizoically became attached to a kelp holdfast and was rafted north, particularly as the UNB herbarium specimen from California is crustose (Table S1).

Although not collected as a rhodolith, the undescribed crust‐forming *Phymatolithon* sp. 1Cal (Figure 10) is noteworthy from a biogeographical standpoint, as this Californian collection was the first molecular confirmation of *Phymatolithon* in the Northeast Pacific (Adey et al., 2018; Gabrielson & Lindstrom, 2018; Kittle et al., 2022). Some historical collections from BC (UBC; A062406, A038657, A035880, A036687; Lipsen & Pitblado, 2024) were initially identified as *Pseudolithophyllum muricatum*. However, *Ps. muricatum* has since undergone taxonomic revision and is now *Crusticorallina muricata* (Hind et al., 2016).

The novel genus *Rhodolithia* is currently monospecific, consisting only of the novel species *R. gracilis*. This species and its constituent varieties were placed in a novel genus because our phylogeny only weakly allied *R. gracilis* var. *gracilis* and *R. gracilis* var. *ramosa* with members of the genus *Phymatolithon*, and the clade had a long branch length consistent with a prolonged period of evolutionary isolation. However, from an anatomical standpoint, *Rhodolithia* shares most vegetative and sporangial characters with *Phymatolithon* but also many with the more distantly related *Leptophytum* (Table 1; Adey et al., 2001; Athanasiadis & Adey, 2006; Kittle et al., 2022). *Phymatolithon* and *Leptophytum* have primarily been distinguished from one another using reproductive morphology such as the branching of spermatangial systems in male conceptacles (Adey et al., 2001; Kittle et al., 2022). The limited sporangial material and lack of gametangial collections for *R. gracilis* complicated the identification of distinguishing reproductive characters for this genus. Therefore, molecular data are the only way to reliably distinguish and identify members of these genera pending the observation of additional reproductive features in *Rhodolithia*. Adding to the conundrum, the phylogenetic analyses conducted in this study (Figure 10) resolved both *Phymatolithon* and *Leptophytum* as polyphyletic, indicating that further taxonomic study is warranted.

### *Rhodolithia gracilis* var. *gracilis* × *ramosa* as a hybrid variety

Analysis of the ITS rDNA region sequences established that *Rhodolithia gracilis* var. *gracilis* × *ramosa* is a hybrid, of which the novel crust‐forming variety *R. gracilis* var. *gracilis* and the novel rhodolith‐forming variety *R. gracilis* var. *ramosa* are the parents. Although the ITS rDNA region has many copies, it is well established that this marker undergoes rapid concerted evolution resulting in the homogenization of sequences within a species (Elder & Turner, 1995; Osuna‐Mascaró et al., 2022). However, in the event of hybridization, particularly in recent hybrids, homogenization often remains incomplete, making the ITS rDNA region uniquely qualified for detecting hybrid organisms (Baldwin et al., 1995; Osuna‐Mascaró et al., 2022). In addition to ITS rDNA region data, analysis of organellar markers determined *R. gracilis* var. *gracilis* × *ramosa* specimens to comprise three genetic variant types.

Both *Rhodolithia gracilis* var. *gracilis* and *R. gracilis* var. *ramosa* have been collected from Vancouver Island sites. However, unlike *R. gracilis* var. *gracilis*, *R. gracilis* var. *ramosa* has not been collected from Haida Gwaii in either rhodolith or crustose form, despite the extensive sampling of corallines conducted there. Additionally, the resulting hybrid has only been collected in Haida Gwaii, although it is possible populations will be uncovered at Bamfield or Tahsis following further sampling. That said, average hydrodynamic forces around rhodolith beds are typically insufficient to transport rhodoliths over long distances (Millar & Gagnon, 2018) suggesting that *R. gracilis* var. *ramosa* could have also occurred at Haida Gwaii, either historically or concurrently. Due to their geographic overlap and ability to interbreed, the parents are considered as varieties rather than species. Although it is still unclear if interbreeding is rare or occurs somewhat commonly, the genetic variation within the hybrid collections suggests the interbreeding was not a single event. However, the two parents were still genetically distinct based on barcode gap analyses and were also morphologically distinct based on current collections given that *R. gracilis* var. *gracilis* has only been collected in crustose form and *R. gracilis* var. *ramosa* has only been collected in rhodolith form. That said, the rhodolith collections for *R. gracilis* var. *ramosa* are all biogenic and lack conceptacles, so it is likely this variety also exists in a reproductive crustose or autogenic rhodolith form that would warrant morphological comparison to *R. gracilis* var. *gracilis*.

Of the *Rhodolithia gracilis* varieties, the rhodolith‐forming hybrid *R. gracilis* var. *gracilis* × *ramosa* is interesting in that it appears to be restricted to greater depths (10–22 m) compared to other BC rhodolith collections that have either a larger depth range or have been observed solely in shallower waters. For example, *Boreolithothamnion astragaloi* has a large depth range of 5–22 m, whereas *B. soriferum* and *B*. sp. 1heterocladum both have smaller ranges, with those in our collections having a maximum depth of ≤11 m. However, this could be a consequence of surveys focusing on shallower sites and the difficulties of sampling coralline crusts in the subtidal.

Interestingly, all our hybrid specimens are biogenic, and none were observed to bear conceptacles, indicating that they are likely reproductively isolated from their parent varieties and propagate through asexual fragmentation. Asexual propagation via fragmentation is a common recruitment strategy in rhodoliths (Bélanger & Gagnon, 2023; Foster, 2001; Freiwald & Henrich, 1994). An inability to reproduce sexually is often seen as a reduction in fitness; however, many red algal populations composed solely of asexual propagules, such as aggregations of *Asparagopsis armata*, have been observed to have increased post‐recruitment survivorship (Wright et al., 2022). Rhodoliths are likely no exception to this trend. Additionally, rhodolith beds are conducive to propagation via fragmentation given that it is commonly mediated by hydrodynamic forces and the abundant grazers of the associated community (Foster, 2001; Herren et al., 2006). This means that the hybrid's apparent restriction to an asexual rhodolith lifestyle likely aids its survival, providing it with a similar fitness compared to other rhodolith‐forming species.

Looking at the genetic variation present in our hybrid collections, the three known types and potential fourth genetic type within the populations suggest the occurrence of multiple hybridization events. In terms of inheritance, the ITS rDNA region demonstrated consistent biparental inheritance across all the putative hybrid collections. However, inheritance of the mitochondrial and plastid genes was not quite so simple, as the Type 2 and Type 3 variants exhibited a different pattern of inheritance for each organelle. It is generally considered that both plastid and mitochondrial genomes are primarily maternally inherited (Greiner et al., 2014), although there have been documented exceptions (Breton & Stewart, 2015; Mignerot et al., 2019; Park et al., 2021). Organellar inheritance patterns in coralline algae are not well understood (Hind & Saunders, 2013); however, research by Mignerot et al. (2019) and Park et al. (2021) has suggested that inheritance can be variably maternal or paternal in different crosses and that mitochondrial and plastid genomes can be inherited from different parents in the same offspring. These alternative avenues of inheritance would allow for the uniparental organellar inheritance seen in Type 1 and hypothetical Type 4 variants as well as the uncoupled organelle inheritance seen in Type 2 and Type 3. However, if organellar inheritance is strictly uniparental, the Type 2 and Type 3 variants (as well as the additive ITS rDNA region data) could also be explained by coalescence of genetically different specimens, as the red algae exhibit a high frequency of natural coalescence (Santelices et al., 2017), and the occurrence of multispecific rhodoliths could provide conditions for somatic fusion (Harvey et al., 2016; Martín et al., 1993; Peña et al., 2021; Teichert et al., 2012). The PCR amplification of a coalesced individual may result in a messy sequence, an additive sequence, or a clean sequence from either genotype (González & Santelices, 2008). However, if coalescence had occurred, we would expect to see some messy or additive organelle sequences in addition to the additive ITS rDNA region sequences. Only clean organelle sequences matching to one parental genotype were observed in our hybrid collections, making coalescence an unlikely possibility.

Given that this project was a preliminary survey of BC rhodolith diversity, collections of the *Rhodolithia gracilis* varieties were not specifically targeted. To fully elucidate the inheritance patterns and evolutionary age of the hybrid variety, additional specimens should be collected for use in population genetics studies. Additional sampling at Haida Gwaii and Tahsis could also determine if *R. gracilis* var. *ramosa* and *R. gracilis* var. *gracilis* × *ramosa* exhibit any geographic overlap and if either variety grows in the crustose morphology. Further sampling should also seek to detect additional hybrid taxa, as with the disturbance of rhodolith beds increasing on a global scale due to factors such as increased storm frequency and human‐related activity, hybridization rates may be increasing. A correlation between habitat disruption and increased hybridization was observed in vascular plants by Beddows and Rose (2018). However, in the case of rhodoliths, initial hybridization would likely occur between parent crustose species in or near disturbed beds as they have higher rates of sexual reproduction than have been observed in rhodoliths.

Although this study has greatly expanded our knowledge on the diversity of rhodoliths in BC, it is only a preliminary step in understanding the true extent of rhodolith beds in the region. Outside of Haida Gwaii, our dataset has been limited to scant collections from Tahsis and Bamfield from early barcoding surveys of the province. Sampling at Haida Gwaii was targeted at rhodolith collections in recent years and resulted in the bulk of our collections; however, the geographic scope of this sampling was still limited to key sites with known rhodolith aggregations. The extent of these rhodolith beds is still unknown, and it is possible that many of our sites are not isolated pockets but instead small pieces of a larger interconnected bed, as rhodolith beds can reach several square kilometers in size (McCoy & Kamenos, 2015). Conducting further collections at Tahsis, Bamfield, and Gosling Island (UBC specimens) is also warranted, as the current specimens likely do not provide the full picture of diversity at these sites. On a larger scale, investigating areas with similar environmental conditions will likely yield other rhodolith sites throughout BC, and as our collections to date have demonstrated a high degree of cryptic diversity, it is probable that beds throughout BC contain additional novel diversity requiring molecular and anatomical assessment. Additional comprehensive sampling may also reveal distribution patterns and dominant species that do not present in our current data. Building upon this initial research to develop a comprehensive taxonomic and geographic baseline will provide the information necessary for future researchers to study ecological interactions within these beds and how they are changing under the impacts of climate change.

## CONCLUSIONS

Rhodolith beds are widespread and rich benthic ecosystems in terms of the communities they support and the ecological services they provide. However, despite their known significance, research concerning them is lagging compared to what is being conducted on the other major benthic ecosystems such as kelp forests and coral reefs. Prior to this study, there were little concrete data on the diversity of rhodolith‐forming coralline algae in BC. Living in the age of accelerating climate change and anthropogenic consequences that affect keystone ecosystems like rhodolith beds makes it critical to have a baseline assessment of the species diversity in a region so that compositional changes and potential bed degradation can be effectively monitored and tracked. This study is only a preliminary step in improving BC rhodolith research so it is comparable to other better‐studied areas of the globe. However, despite its initiatory nature, this research uncovered a swath of novel diversity and topics to be expanded upon in future research while highlighting the importance of using a combination of molecular and anatomical data during diversity assessments. Going forward, a spotlight should be placed on BC with the goal of determining the true extent of rhodolith beds in the Northeast Pacific and uncovering the remaining cryptic diversity within them.

## AUTHOR CONTRIBUTIONS

**Keelie E. Taylor:** Data curation (equal); formal analysis (equal); funding acquisition (supporting); investigation (equal); methodology (equal); writing – original draft (lead); writing – review and editing (equal). **Gary W. Saunders:** Conceptualization (lead); data curation (equal); formal analysis (equal); funding acquisition (lead); investigation (equal); methodology (equal); resources (lead); writing – review and editing (equal).

## FUNDING INFORMATION

We would like to thank the Northeast Algal Society Development Committee for awarding the NEAS Graduate Research Grant that helped fund our second season of sampling. Discovery and Accelerator grants to GWS from the Natural Sciences and Engineering Research Council of Canada and funding from the Canada Foundation for Innovation and the New Brunswick Innovation Foundation supported this research.

## Supporting information


**Table S1.** Collection data and GenBank accession numbers for rhodolith, crustose and geniculate coralline specimens used in morphological and/or molecular analyses. Holotype and isotype specimens are indicated following the herbarium ID.

**Table S2.** Intraspecific and interspecific variation in the *psb*A (~950 bp) gene region for rhodolith‐forming and crustose genetic groups of *Boreolithothamnion* and *Rhodolithia* gen. nov., where *n* is the number of sequences.
