# Supplementary material for: Assessment of rhodolith‐forming species diversity in British Columbia uncovers novel cryptic diversity in the genera Boreolithothamnion and Rhodolithia gen. nov. (Florideophyceae, Rhodophyta) and the occurrence of hybrid rhodoliths
Source: J Phycol. 2025 Aug 13;61(5):1371–93. doi: 10.1111/jpy.70066 (PMC12547645; doi:10.1111/jpy.70066)
Supplement: Supplementary file 1 — Table S1. Collection data and GenBank accession numbers for rhodolith, crustose and geniculate coralline specimens used in morphological and/or molecular analyses. Holotype and isotype specimens are indicated following the herbarium ID. [file JPY-61-1371-s002.docx]

**Table S1.**

| **Taxon name** | **Herbarium ID** | **Collection data** | **GenBank acc. No. (COI-5P)** | **GenBank acc. No. (*rbc*L-3P)** | **GenBank acc. No. (*rbc*L)** | **Genbank acc. No. (*psb*A)** | **Genbank acc. No. (LSU)** | **Genbank acc. No. (ITS)** |
| --- | --- | --- | --- | --- | --- | --- | --- | --- |
| *Amphiroa zonata* | GWS013768 | Mid Intertidal on rock, articulated, Katsuura, Chiba prefecture, Japan, 35.139722 140.321389, 5.8.2009, K. Hind & M. Baba | HM916709 |  | MH777616 | MH777612 | PQ137881 |  |
| *Amphiroa zonata* | GWS013772 | Mid Intertidal on rock, articulated, Katsuura, Chiba prefecture, Japan, 35.139722 140.321389, 5.8.2009, K. Hind & M. Baba | HM916656 |  |  |  |  |  |
| *Boreolithothamnion astragaloi* | GWS020737 | Subtidal (6 m) on invert shell, rhodolith, Kwuna I., Haida Gwaii, British Columbia, Canada, 53.21722 -131.98589, 6.6.2010, G.W. Saunders & K. Dixon | HQ545049 |  |  |  |  |  |
| *Boreolithothamnion astragaloi* | GWS028208 | Subtidal (22 m) (anchor dredge), rhodolith, Hotspring I. (back side in channel), Gwaii Haanas, British Columbia, Canada, 52.5779 -131.43768,  7.7.2011, G.W. Saunders & K. Dixon |  | PQ882881 |  |  |  |  |
| *Boreolithothamnion astragaloi* | GWS038778 | Subtidal (5 m) on rock & shell, rhodolith, Renner Pass, Skidegate Ch., Haida Gwaii, British Columbia, Canada, 53.19446 -132.13161, 13.7.2015, G.W. Saunders, M. Bruce & T. Bringloe | MW536948 |  |  |  |  |  |
| *Boreolithothamnion astragaloi* | GWS045700 | Subtidal (6 m), rhodolith, Channel btw Murchison & Faraday I., Gwaii Haanas, British Columbia, Canada, 52.59687 -131.47513, 20.7.2019, G.W. Saunders & C. Brooks |  | PQ882968 |  |  |  |  |
| *Boreolithothamnion astragaloi* | GWS045707 | Subtidal (6 m), rhodolith, Channel btw Murchison & Faraday I., Gwaii Haanas, British Columbia, Canada, 52.59687 -131.47513, 20.7.2019, G.W. Saunders & C. Brooks |  |  |  | PQ888696 |  |  |
| *Boreolithothamnion astragaloi* | GWS046703 | Subtidal (7 m), rhodolith, Channel btw Murchison & Faraday I., Gwaii Haanas, British Columbia, Canada, 52.59703 –  131.47345, 27.7.2022, G.W. Saunders & C. Brooks |  |  | PQ882794 | PQ888442 | PQ883058 |  |
| *Boreolithothamnion astragaloi* | GWS046705 | Subtidal (7 m), rhodolith, Channel btw Murchison & Faraday I., Gwaii Haanas, British Columbia, Canada, 52.59703 -131.47345, 27.7.2022, G.W. Saunders & C. Brooks |  | PQ882994 |  | PQ888689 |  |  |
| *Boreolithothamnion astragaloi* | GWS046706 | Subtidal (7 m), rhodolith, Channel btw Murchison & Faraday I., Gwaii Haanas, British Columbia, Canada, 52.59703 -131.47345, 27.7.2022, G.W. Saunders & C. Brooks | PQ883050 | PQ882989 |  | PQ888682 |  |  |
| *Boreolithothamnion astragaloi* | GWS046707 | Subtidal (7 m), rhodolith, Channel btw Murchison & Faraday I., Gwaii Haanas, British Columbia, Canada, 52.59703 -131.47345, 27.7.2022, G.W. Saunders & C. Brooks | PQ883044 | PQ882941 |  | PQ888617 |  |  |
| *Boreolithothamnion astragaloi* | GWS046708 | Subtidal (7 m), rhodolith, Channel btw Murchison & Faraday I., Gwaii Haanas, British Columbia, Canada, 52.59703 –  131.47345, 27.7.2022, G.W. Saunders & C. Brooks | PQ883042 | PQ882914 |  | PQ888585 |  |  |
| *Boreolithothamnion astragaloi* | GWS046710 | Subtidal (7 m), rhodolith, Channel btw Murchison & Faraday I., Gwaii Haanas, British Columbia, Canada, 52.59703 -131.47345, 27.7.2022, G.W. Saunders & C. Brooks |  | PQ882977 |  | PQ888665 |  |  |
| *Boreolithothamnion astragaloi* | GWS046712 | Subtidal (7 m), rhodolith, Channel btw Murchison & Faraday I., Gwaii Haanas, British Columbia, Canada, 52.59703 -131.47345, 27.7.2022, G.W. Saunders & C. Brooks |  | PQ882875 |  | PQ888546 |  |  |
| *Boreolithothamnion astragaloi* | GWS046713 | Subtidal (7 m), rhodolith, Channel btw Murchison & Faraday I., Gwaii Haanas, British Columbia, Canada, 52.59703 -131.47345, 27.7.2022, G.W. Saunders & C. Brooks |  | PQ882938 |  | PQ888613 |  |  |
| *Boreolithothamnion astragaloi* | GWS046714 | Subtidal (7 m), rhodolith, Channel btw Murchison & Faraday I., Gwaii Haanas, British Columbia, Canada, 52.59703 –  131.47345, 27.7.2022, G.W. Saunders & C. Brooks |  | PQ882819 |  | PQ888477 |  |  |
| *Boreolithothamnion astragaloi* | GWS046715 | Subtidal (7 m), rhodolith, Channel btw Murchison & Faraday I., Gwaii Haanas, British Columbia, Canada, 52.59703 -131.47345, 27.7.2022, G.W. Saunders & C. Brooks |  |  |  | PQ888453 |  |  |
| *Boreolithothamnion astragaloi* | GWS046716 | Subtidal (7 m), rhodolith, Channel btw Murchison & Faraday I., Gwaii Haanas, British Columbia, Canada, 52.59703 -131.47345, 27.7.2022, G.W. Saunders & C. Brooks |  | PQ882945 |  | PQ888624 |  |  |
| *Boreolithothamnion astragaloi* | GWS046717 | Subtidal (7 m), rhodolith, Channel btw Murchison & Faraday I., Gwaii Haanas, British Columbia, Canada, 52.59703 -131.47345, 27.7.2022, G.W. Saunders & C. Brooks |  | PQ882814 |  | PQ888473 |  |  |
| *Boreolithothamnion astragaloi* | GWS046718 | Subtidal (7 m), rhodolith, Channel btw Murchison & Faraday I., Gwaii Haanas, British Columbia, Canada, 52.59703 –  131.47345, 27.7.2022, G.W. Saunders & C. Brooks |  | PQ882916 |  | PQ888586 |  |  |
| *Boreolithothamnion astragaloi* | GWS046719 | Subtidal (7 m), rhodolith, Channel btw Murchison & Faraday I., Gwaii Haanas, British Columbia, Canada, 52.59703 -131.47345, 27.7.2022, G.W. Saunders & C. Brooks |  | PQ882899 |  | PQ888575 |  |  |
| *Boreolithothamnion astragaloi* | GWS046720 | Subtidal (7 m), rhodolith, Channel btw Murchison & Faraday I., Gwaii Haanas, British Columbia, Canada, 52.59703 -131.47345, 27.7.2022, G.W. Saunders & C. Brooks |  | PQ882951 |  | PQ888631 |  |  |
| *Boreolithothamnion astragaloi* | GWS046723 | Subtidal (7 m), rhodolith, Channel btw Murchison & Faraday I., Gwaii Haanas, British Columbia, Canada, 52.59703 -131.47345, 27.7.2022, G.W. Saunders & C. Brooks |  | PQ882825 |  | PQ888483 |  |  |
| *Boreolithothamnion astragaloi* | GWS046724 | Subtidal (7 m), rhodolith, Channel btw Murchison & Faraday I., Gwaii Haanas, British Columbia, Canada, 52.59703 –  131.47345, 27.7.2022, G.W. Saunders & C. Brooks |  | PQ882799 |  | PQ888449 |  |  |
| *Boreolithothamnion astragaloi* | GWS046725 | Subtidal (7 m), rhodolith, Channel btw Murchison & Faraday I., Gwaii Haanas, British Columbia, Canada, 52.59703 -131.47345, 27.7.2022, G.W. Saunders & C. Brooks |  | PQ882810 |  | PQ888470 |  |  |
| *Boreolithothamnion astragaloi* | GWS046726 | Subtidal (7 m), rhodolith, Channel btw Murchison & Faraday I., Gwaii Haanas, British Columbia, Canada, 52.59703 -131.47345, 27.7.2022, G.W. Saunders & C. Brooks |  | PQ882870 |  | PQ888540 |  |  |
| *Boreolithothamnion astragaloi* | GWS046728 | Subtidal (7 m), rhodolith, Channel btw Murchison & Faraday I., Gwaii Haanas, British Columbia, Canada, 52.59703 -131.47345, 27.7.2022, G.W. Saunders & C. Brooks |  | PQ882973 |  | PQ888661 |  |  |
| *Boreolithothamnion astragaloi* | GWS046729 | Subtidal (7 m), rhodolith, Channel btw Murchison & Faraday I., Gwaii Haanas, British Columbia, Canada, 52.59703 –  131.47345, 27.7.2022, G.W. Saunders & C. Brooks |  | PQ882817 |  |  |  |  |
| *Boreolithothamnion astragaloi* | GWS046730 | Subtidal (7 m), rhodolith, Channel btw Murchison & Faraday I., Gwaii Haanas, British Columbia, Canada, 52.59703 -131.47345, 27.7.2022, G.W. Saunders & C. Brooks |  | PQ882988 |  |  |  |  |
| *Boreolithothamnion astragaloi* | GWS046731 | Subtidal (7 m), rhodolith, Channel btw Murchison & Faraday I., Gwaii Haanas, British Columbia, Canada, 52.59703 -131.47345, 27.7.2022, G.W. Saunders & C. Brooks |  | PQ882843 |  |  |  |  |
| *Boreolithothamnion astragaloi* | GWS046733 | Subtidal (7 m), rhodolith, Channel btw Murchison & Faraday I., Gwaii Haanas, British Columbia, Canada, 52.59703 -131.47345, 27.7.2022, G.W. Saunders & C. Brooks |  | PQ882942 |  | PQ888618 |  |  |
| *Boreolithothamnion astragaloi* | GWS046734 | Subtidal (7 m), rhodolith, Channel btw Murchison & Faraday I., Gwaii Haanas, British Columbia, Canada, 52.59703 –  131.47345, 27.7.2022, G.W. Saunders & C. Brooks |  | PQ882797 |  |  |  |  |
| *Boreolithothamnion astragaloi* | GWS046735 | Subtidal (7 m), rhodolith, Channel btw Murchison & Faraday I., Gwaii Haanas, British Columbia, Canada, 52.59703 -131.47345, 27.7.2022, G.W. Saunders & C. Brooks |  |  |  | PQ888666 |  |  |
| *Boreolithothamnion astragaloi* | GWS046736 | Subtidal (7 m), rhodolith, Channel btw Murchison & Faraday I., Gwaii Haanas, British Columbia, Canada, 52.59703 -131.47345, 27.7.2022, G.W. Saunders & C. Brooks |  |  |  | PQ888708 |  |  |
| *Boreolithothamnion astragaloi* | GWS046737 | Subtidal (7 m), rhodolith, Channel btw Murchison & Faraday I., Gwaii Haanas, British Columbia, Canada, 52.59703 -131.47345, 27.7.2022, G.W. Saunders & C. Brooks |  |  |  | PQ888680 |  |  |
| *Boreolithothamnion astragaloi* | GWS046739 | Subtidal (7 m), rhodolith, Channel btw Murchison & Faraday I., Gwaii Haanas, British Columbia, Canada, 52.59703 –  131.47345, 27.7.2022, G.W. Saunders & C. Brooks |  |  |  | PQ888469 |  |  |
| *Boreolithothamnion astragaloi* | GWS046740 | Subtidal (7 m), rhodolith, Channel btw Murchison & Faraday I., Gwaii Haanas, British Columbia, Canada, 52.59703 -131.47345, 27.7.2022, G.W. Saunders & C. Brooks |  |  |  | PQ888633 |  |  |
| *Boreolithothamnion astragaloi* | GWS046741 | Subtidal (7 m), rhodolith, Channel btw Murchison & Faraday I., Gwaii Haanas, British Columbia, Canada, 52.59703 -131.47345, 27.7.2022, G.W. Saunders & C. Brooks |  |  |  | PQ888530 |  |  |
| *Boreolithothamnion astragaloi* | GWS046742 | Subtidal (7 m), rhodolith, Channel btw Murchison & Faraday I., Gwaii Haanas, British Columbia, Canada, 52.59703 -131.47345, 27.7.2022, G.W. Saunders & C. Brooks |  |  |  | PQ888655 |  |  |
| *Boreolithothamnion astragaloi* | GWS046745 | Subtidal (7 m), rhodolith, Channel btw Murchison & Faraday I., Gwaii Haanas, British Columbia, Canada, 52.59703 –  131.47345, 27.7.2022, G.W. Saunders & C. Brooks |  |  |  | PQ888508 |  |  |
| *Boreolithothamnion astragaloi* | GWS046746 | Subtidal (7 m), rhodolith, Channel btw Murchison & Faraday I., Gwaii Haanas, British Columbia, Canada, 52.59703 -131.47345, 27.7.2022, G.W. Saunders & C. Brooks |  |  |  | PQ888654 |  |  |
| *Boreolithothamnion astragaloi* | GWS046747 | Subtidal (7 m), rhodolith, Channel btw Murchison & Faraday I., Gwaii Haanas, British Columbia, Canada, 52.59703 -131.47345, 27.7.2022, G.W. Saunders & C. Brooks |  |  |  | PQ888583 |  |  |
| *Boreolithothamnion astragaloi* | GWS046748 | Subtidal (7 m), rhodolith, Channel btw Murchison & Faraday I., Gwaii Haanas, British Columbia, Canada, 52.59703 -131.47345, 27.7.2022, G.W. Saunders & C. Brooks |  |  |  | PQ888431 |  |  |
| *Boreolithothamnion astragaloi* | GWS046749 | Subtidal (7 m), rhodolith, Channel btw Murchison & Faraday I., Gwaii Haanas, British Columbia, Canada, 52.59703 –  131.47345, 27.7.2022, G.W. Saunders & C. Brooks |  |  |  | PQ888558 |  |  |
| *Boreolithothamnion astragaloi* | GWS046904 | Subtidal (6 m), rhodolith, Murchison I. anchorage east entrance, Gwaii Haanas, British Columbia, Canada, 52.595183 -131.464537, 28.7.2022, G.W. Saunders & C. Brooks |  | PQ882963 |  | PQ888646 |  |  |
| *Boreolithothamnion astragaloi* | GWS046906 | Subtidal (6 m), rhodolith, Murchison I. anchorage east entrance, Gwaii Haanas, British Columbia, Canada, 52.595183 -131.464537, 28.7.2022, G.W. Saunders & C. Brooks |  | PQ882959 |  |  |  |  |
| *Boreolithothamnion astragaloi* | GWS046908 | Subtidal (6 m), rhodolith, Murchison I. anchorage east entrance, Gwaii Haanas, British Columbia, Canada, 52.595183 -131.464537, 28.7.2022, G.W. Saunders & C. Brooks |  | PQ883005 |  | PQ888711 |  |  |
| *Boreolithothamnion astragaloi* | GWS046909 | Subtidal (6 m), rhodolith, Murchison I. anchorage east  entrance, Gwaii Haanas, British Columbia, Canada, 52.595183 -131.464537, 28.7.2022, G.W. Saunders & C. Brooks |  | PQ882854 |  | PQ888513 |  |  |
| *Boreolithothamnion astragaloi* | GWS046910 | Subtidal (6 m), rhodolith, Murchison I. anchorage east entrance, Gwaii Haanas, British Columbia, Canada, 52.595183 -131.464537, 28.7.2022, G.W. Saunders & C. Brooks |  | PQ882948 |  | PQ888626 |  |  |
| *Boreolithothamnion astragaloi* | GWS046917 | Subtidal (20 m), rhodolith, Nereo Pinnacle, Faraday Pass east side, Gwaii Haanas, British Columbia, Canada, 52.6199 -131.4601, 29.7.2022, G.W. Saunders & C. Brooks |  |  |  | PQ888629 |  |  |
| *Boreolithothamnion astragaloi* | GWS046918 | Subtidal (20 m), rhodolith, Nereo Pinnacle, Faraday Pass east side, Gwaii Haanas, British Columbia, Canada, 52.6199 -131.4601, 29.7.2022, G.W. Saunders & C. Brooks |  | PQ882970 |  |  |  |  |
| *Boreolithothamnion astragaloi* | GWS046919 | Subtidal (20 m), rhodolith, Nereo Pinnacle, Faraday Pass east side, Gwaii Haanas, British Columbia, Canada, 52.6199 -131.4601, 29.7.2022, G.W. Saunders & C. Brooks |  | PQ882958 |  | PQ888641 |  |  |
| *Boreolithothamnion astragaloi* | GWS046920 | Subtidal (20 m), rhodolith, Nereo Pinnacle, Faraday Pass east side, Gwaii Haanas, British Columbia, Canada, 52.6199 -131.4601, 29.7.2022, G.W. Saunders & C. Brooks |  |  |  | PQ888441 |  |  |
| *Boreolithothamnion astragaloi* | GWS046921 | Subtidal (20 m), rhodolith, Nereo Pinnacle, Faraday Pass east side, Gwaii Haanas, British Columbia, Canada, 52.6199 -131.4601, 29.7.2022, G.W. Saunders & C. Brooks |  |  |  | PQ888714 |  |  |
| *Boreolithothamnion astragaloi* | GWS046922 | Subtidal (20 m), rhodolith, Nereo Pinnacle, Faraday Pass east side, Gwaii Haanas, British Columbia, Canada, 52.6199 -131.4601, 29.7.2022, G.W. Saunders & C. Brooks |  |  |  | PQ888567 |  |  |
| *Boreolithothamnion astragaloi* | GWS046923 | ubtidal (20 m), rhodolith, Nereo Pinnacle, Faraday Pass east side, Gwaii Haanas, British Columbia, Canada, 52.6199 -131.4601, 29.7.2022, G.W. Saunders & C. Brooks |  |  |  | PQ888526 |  |  |
| *Boreolithothamnion astragaloi* | GWS046924 | Subtidal (20 m), rhodolith, Nereo Pinnacle, Faraday Pass east side, Gwaii Haanas, British Columbia, Canada, 52.6199 -131.4601, 29.7.2022, G.W. Saunders & C. Brooks |  | PQ882978 |  |  |  |  |
| *Boreolithothamnion astragaloi* | GWS046925 | Subtidal (20 m), rhodolith, Nereo Pinnacle, Faraday Pass east side, Gwaii Haanas, British Columbia, Canada, 52.6199 -131.4601, 29.7.2022, G.W. Saunders & C. Brooks |  |  |  | PQ888674 |  |  |
| *Boreolithothamnion astragaloi* | GWS046926 | Subtidal (20 m), rhodolith, Nereo Pinnacle, Faraday Pass east side, Gwaii Haanas, British Columbia, Canada, 52.6199 -131.4601, 29.7.2022, G.W. Saunders & C. Brooks |  | PQ882798 |  | PQ888448 |  |  |
| *Boreolithothamnion astragaloi* | GWS047963 | Subtidal (10-30 ft) on turbin snail, crust, Faraday I., N Site, Plot 1 (10-30 ft), Gwaii Haanas, British Columbia, Canada, 52.61523 -131.46466, 6.9.2021, G.W. Saunders & C. Brooks |  | PQ882915 |  |  |  |  |
| *Boreolithothamnion astragaloi* | GWS047999 (Isotype) | Subtidal (10 m) on bottom, rhodolith, Channel btw Murchison & Faraday I., Gwaii Haanas, British Columbia, Canada, 52.59687 -131.47513, 7.9.2021, G.W. Saunders & C. Brooks |  |  |  |  |  |  |
| *Boreolithothamnion astragaloi* | GWS048562 (Isotype) | Subtidal (5 m), rhodolith, Channel btw Murchison & Faraday I., Gwaii Haanas, British Columbia, Canada, 52.59687 -131.47513, 2.8.2022, G.W. Saunders & C. Brooks |  |  |  | PQ888723 |  |  |
| *Boreolithothamnion astragaloi* | GWS048563 (Isotype) | Subtidal (5 m), rhodolith, Channel btw Murchison & Faraday I., Gwaii Haanas, British Columbia, Canada, 52.59687 -131.47513, 2.8.2022, G.W. Saunders & C. Brooks |  |  |  | PQ888710 |  |  |
| *Boreolithothamnion astragaloi* | GWS048564 (Isotype) | Subtidal (5 m), rhodolith, Channel btw Murchison & Faraday I., Gwaii Haanas, British Columbia, Canada, 52.59687 -131.47513, 2.8.2022, G.W. Saunders & C. Brooks |  | PQ882919 |  | PQ888588 |  |  |
| *Boreolithothamnion astragaloi* | GWS048566 (Isotype) | Subtidal (5 m), rhodolith, Channel btw Murchison & Faraday I., Gwaii Haanas, British Columbia, Canada, 52.59687 -131.47513, 2.8.2022, G.W. Saunders & C. Brooks |  |  |  | PQ888606 |  |  |
| *Boreolithothamnion astragaloi* | GWS048567 (Isotype) | Subtidal (5 m), rhodolith, Channel btw Murchison & Faraday I., Gwaii Haanas, British Columbia, Canada, 52.59687 -131.47513, 2.8.2022, G.W. Saunders & C. Brooks |  | PQ882813 |  |  |  |  |
| *Boreolithothamnion astragaloi* | GWS048568 (Isotype) | Subtidal (5 m), rhodolith, Channel btw Murchison & Faraday I., Gwaii Haanas, British Columbia, Canada, 52.59687 -131.47513, 2.8.2022, G.W. Saunders & C. Brooks |  | PQ882929 |  |  |  |  |
| *Boreolithothamnion astragaloi* | GWS048569 (Isotype) | Subtidal (5 m), rhodolith, Channel btw Murchison & Faraday I., Gwaii Haanas, British Columbia, Canada, 52.59687 -131.47513, 2.8.2022, G.W. Saunders & C. Brooks |  | PQ882839 |  |  |  |  |
| *Boreolithothamnion astragaloi* | GWS048570 (Isotype) | Subtidal (5 m), rhodolith, Channel btw Murchison & Faraday I., Gwaii Haanas, British Columbia, Canada, 52.59687 -131.47513, 2.8.2022, G.W. Saunders & C. Brooks |  |  |  | PQ888565 |  |  |
| *Boreolithothamnion astragaloi* | GWS048571 (Isotype) | Subtidal (5 m), rhodolith, Channel btw Murchison & Faraday I., Gwaii Haanas, British Columbia, Canada, 52.59687 -131.47513, 2.8.2022, G.W. Saunders & C. Brooks |  | PQ882923 |  |  |  |  |
| *Boreolithothamnion astragaloi* | GWS048572 (Isotype) | Subtidal (5 m), rhodolith, Channel btw Murchison & Faraday I., Gwaii Haanas, British Columbia, Canada, 52.59687 -131.47513, 2.8.2022, G.W. Saunders & C. Brooks |  |  |  | PQ888699 |  |  |
| *Boreolithothamnion astragaloi* | GWS048573 (Isotype) | Subtidal (5 m), rhodolith, Channel btw Murchison & Faraday I., Gwaii Haanas, British Columbia, Canada, 52.59687 -131.47513, 2.8.2022, G.W. Saunders & C. Brooks |  |  |  | PQ888465 |  |  |
| *Boreolithothamnion astragaloi* | GWS048577 (Isotype) | Subtidal (5 m), rhodolith, Channel btw Murchison & Faraday I., Gwaii Haanas, British Columbia, Canada, 52.59687 -131.47513, 2.8.2022, G.W. Saunders & C. Brooks |  |  |  | PQ888608 |  |  |
| *Boreolithothamnion astragaloi* | GWS048579 (Isotype) | Subtidal (5 m), rhodolith, Channel btw Murchison & Faraday I., Gwaii Haanas, British Columbia, Canada, 52.59687 -131.47513, 2.8.2022, G.W. Saunders & C. Brooks |  | PQ882964 |  |  |  |  |
| *Boreolithothamnion astragaloi* | GWS048580 (Isotype) | Subtidal (5 m), rhodolith, Channel btw Murchison & Faraday I., Gwaii Haanas, British Columbia, Canada, 52.59687 -131.47513, 2.8.2022, G.W. Saunders & C. Brooks |  | PQ882906 |  |  |  |  |
| *Boreolithothamnion astragaloi* | GWS048581 (Isotype) | Subtidal (5 m), rhodolith, Channel btw Murchison & Faraday I., Gwaii Haanas, British Columbia, Canada, 52.59687 -131.47513, 2.8.2022, G.W. Saunders & C. Brooks |  | PQ882802 |  |  |  |  |
| *Boreolithothamnion astragaloi* | GWS048582 (Isotype) | Subtidal (5 m), rhodolith, Channel btw Murchison & Faraday I., Gwaii Haanas, British Columbia, Canada, 52.59687 -131.47513, 2.8.2022, G.W. Saunders & C. Brooks |  |  |  | PQ888657 |  |  |
| *Boreolithothamnion astragaloi* | GWS048583 (Isotype) | Subtidal (5 m), rhodolith, Channel btw Murchison & Faraday I., Gwaii Haanas, British Columbia, Canada, 52.59687 -131.47513, 2.8.2022, G.W. Saunders & C. Brooks |  | PQ882821 |  |  |  |  |
| *Boreolithothamnion astragaloi* | GWS048584 (Isotype) | Subtidal (5 m), rhodolith, Channel btw Murchison & Faraday I., Gwaii Haanas, British Columbia, Canada, 52.59687 -131.47513, 2.8.2022, G.W. Saunders & C. Brooks |  | PQ882803 |  |  |  |  |
| *Boreolithothamnion astragaloi* | GWS048585 (Isotype) | Subtidal (5 m), rhodolith, Channel btw Murchison & Faraday I., Gwaii Haanas, British Columbia, Canada, 52.59687 -131.47513, 2.8.2022, G.W. Saunders & C. Brooks |  | PQ882898 |  |  |  |  |
| *Boreolithothamnion astragaloi* | GWS048586 (Isotype) | Subtidal (5 m), rhodolith, Channel btw Murchison & Faraday I., Gwaii Haanas, British Columbia, Canada, 52.59687 -131.47513, 2.8.2022, G.W. Saunders & C. Brooks |  | PQ882939 |  |  |  |  |
| *Boreolithothamnion astragaloi* | GWS048588 (Isotype) | Subtidal (5 m), rhodolith, Channel btw Murchison & Faraday I., Gwaii Haanas, British Columbia, Canada, 52.59687 -131.47513, 2.8.2022, G.W. Saunders & C. Brooks |  | PQ882829 |  |  |  |  |
| *Boreolithothamnion astragaloi* | GWS048591 (Isotype) | Subtidal (5 m), rhodolith, Channel btw Murchison & Faraday I., Gwaii Haanas, British Columbia, Canada, 52.59687 -131.47513, 2.8.2022, G.W. Saunders & C. Brooks |  | PQ882927 |  |  |  |  |
| *Boreolithothamnion astragaloi* | GWS048592 (Isotype) | Subtidal (5 m), rhodolith, Channel btw Murchison & Faraday I., Gwaii Haanas, British Columbia, Canada, 52.59687 -131.47513, 2.8.2022, G.W. Saunders & C. Brooks |  | PQ883014 |  |  |  |  |
| *Boreolithothamnion astragaloi* | GWS048596 (Isotype) | Subtidal (5 m), rhodolith, Channel btw Murchison & Faraday I., Gwaii Haanas, British Columbia, Canada, 52.59687 -131.47513, 2.8.2022, G.W. Saunders & C. Brooks |  | PQ882925 |  |  |  |  |
| *Boreolithothamnion astragaloi* | GWS048597 (Isotype) | Subtidal (5 m), rhodolith, Channel btw Murchison & Faraday I., Gwaii Haanas, British Columbia, Canada, 52.59687 -131.47513, 2.8.2022, G.W. Saunders & C. Brooks |  | PQ882850 |  |  |  |  |
| *Boreolithothamnion astragaloi* | GWS048598 (Isotype) | Subtidal (5 m), rhodolith, Channel btw Murchison & Faraday I., Gwaii Haanas, British Columbia, Canada, 52.59687 -131.47513, 2.8.2022, G.W. Saunders & C. Brooks |  | PQ882975 |  |  |  |  |
| *Boreolithothamnion astragaloi* | GWS048599 (Isotype) | Subtidal (5 m), rhodolith, Channel btw Murchison & Faraday I., Gwaii Haanas, British Columbia, Canada, 52.59687 -131.47513, 2.8.2022, G.W. Saunders & C. Brooks |  | PQ882981 |  |  |  |  |
| *Boreolithothamnion astragaloi* | GWS048600 (Holotype) | Subtidal (5 m), rhodolith, Channel btw Murchison & Faraday I., Gwaii Haanas, British Columbia, Canada, 52.59687 -131.47513, 2.8.2022, G.W. Saunders & C. Brooks |  | PQ882800 |  |  |  |  |
| *Boreolithothamnion astragaloi* | GWS048601 (Isotype) | Subtidal (5 m), rhodolith, Channel btw Murchison & Faraday I., Gwaii Haanas, British Columbia, Canada, 52.59687 -131.47513, 2.8.2022, G.W. Saunders & C. Brooks |  |  |  | PQ888491 |  |  |
| *Boreolithothamnion astragaloi* | GWS048606 (Isotype) | Subtidal (5 m), rhodolith, Channel btw Murchison & Faraday I., Gwaii Haanas, British Columbia, Canada, 52.59687 -131.47513, 2.8.2022, G.W. Saunders & C. Brooks |  | PQ882823 |  |  |  |  |
| *Boreolithothamnion astragaloi* | GWS048607 (Isotype) | Subtidal (5 m), rhodolith, Channel btw Murchison & Faraday I., Gwaii Haanas, British Columbia, Canada, 52.59687 -131.47513, 2.8.2022, G.W. Saunders & C. Brooks |  | PQ883019 |  |  |  |  |
| *Boreolithothamnion astragaloi* | GWS048609 (Isotype) | Subtidal (5 m), rhodolith, Channel btw Murchison & Faraday I., Gwaii Haanas, British Columbia, Canada, 52.59687 -131.47513, 2.8.2022, G.W. Saunders & C. Brooks |  | PQ882816 |  |  |  |  |
| *Boreolithothamnion astragaloi* | GWS048610 (Isotype) | Subtidal (5 m), rhodolith, Channel btw Murchison & Faraday I., Gwaii Haanas, British Columbia, Canada, 52.59687 -131.47513, 2.8.2022, G.W. Saunders & C. Brooks |  | PQ882984 |  |  |  |  |
| *Boreolithothamnion astragaloi* | GWS048611 (Isotype) | Subtidal (5 m), rhodolith, Channel btw Murchison & Faraday I., Gwaii Haanas, British Columbia, Canada, 52.59687 -131.47513, 2.8.2022, G.W. Saunders & C. Brooks |  | PQ882905 |  |  |  |  |
| *Boreolithothamnion astragaloi* | GWS048613 (Isotype) | Subtidal (5 m), rhodolith, Channel btw Murchison & Faraday I., Gwaii Haanas, British Columbia, Canada, 52.59687 -131.47513, 2.8.2022, G.W. Saunders & C. Brooks |  | PQ882841 |  |  |  |  |
| *Boreolithothamnion astragaloi* | GWS048614 (Isotype) | Subtidal (5 m), rhodolith, Channel btw Murchison & Faraday I., Gwaii Haanas, British Columbia, Canada, 52.59687 -131.47513, 2.8.2022, G.W. Saunders & C. Brooks |  | PQ882952 |  |  |  |  |
| *Boreolithothamnion astragaloi* | GWS048615 (Isotype) | Subtidal (5 m), rhodolith, Channel btw Murchison & Faraday I., Gwaii Haanas, British Columbia, Canada, 52.59687 -131.47513, 2.8.2022, G.W. Saunders & C. Brooks |  | PQ882864 |  |  |  |  |
| *Boreolithothamnion astragaloi* | GWS048617 (Isotype) | Subtidal (5 m), rhodolith, Channel btw Murchison & Faraday I., Gwaii Haanas, British Columbia, Canada, 52.59687 -131.47513, 2.8.2022, G.W. Saunders & C. Brooks |  |  |  | PQ888504 |  |  |
| *Boreolithothamnion astragaloi* | GWS048621 (Isotype) | Subtidal (5 m), rhodolith, Channel btw Murchison & Faraday I., Gwaii Haanas, British Columbia, Canada, 52.59687 -131.47513, 2.8.2022, G.W. Saunders & C. Brooks |  | PQ882834 |  |  |  |  |
| *Boreolithothamnion astragaloi* | GWS048623 (Isotype) | Subtidal (5 m), rhodolith, Channel btw Murchison & Faraday I., Gwaii Haanas, British Columbia, Canada, 52.59687 -131.47513, 2.8.2022, G.W. Saunders & C. Brooks |  |  |  | PQ888671 |  |  |
| *Boreolithothamnion astragaloi* | GWS048624 (Isotype) | Subtidal (5 m), rhodolith, Channel btw Murchison & Faraday I., Gwaii Haanas, British Columbia, Canada, 52.59687 -131.47513, 2.8.2022, G.W. Saunders & C. Brooks |  | PQ882855 |  |  |  |  |
| *Boreolithothamnion astragaloi* | GWS048625 (Isotype) | Subtidal (5 m), rhodolith, Channel btw Murchison & Faraday I., Gwaii Haanas, British Columbia, Canada, 52.59687 -131.47513, 2.8.2022, G.W. Saunders & C. Brooks |  | PQ882876 |  |  |  |  |
| *Boreolithothamnion astragaloi* | GWS049703 | Subtidal (6.5 m), rhodolith, Hotspring I., British Columbia, Canada, 52.57847 -131.44319, 15.7.2023, L. Lee, D. Okamoto, L. Vigneault & A. Galloway |  | PQ882947 |  | PQ888625 |  |  |
| *Boreolithothamnion astragaloi* | GWS049800 | Subtidal (11 m), rhodolith, Murchison NW (restoration site near Plot 1), British Columbia, Canada, 22.7.2023, L. Lee, D. Okamoto, L. Vigneault & A. Galloway |  |  |  | PQ888502 |  |  |
| *Boreolithothamnion astragaloi* | GWS049801 | Subtidal (11 m), rhodolith, Murchison NW (restoration site near Plot 1), British Columbia, Canada, 22.7.2023, L. Lee, D. Okamoto, L. Vigneault & A. Galloway |  |  |  | PQ888693 |  |  |
| *Boreolithothamnion astragaloi* | GWS049814 | Subtidal (10.4 m), rhodolith, Tanu I., British Columbia, Canada, 52.76233 -131.61822, 23.7.2023, L. Lee, D. Okamoto, L. Vigneault & A. Galloway |  |  |  | PQ888652 |  |  |
| *Boreolithothamnion astragaloi* | GWS049816 | Subtidal (10.4 m), rhodolith, Tanu I., British Columbia, Canada, 52.76233 -131.61822, 23.7.2023, L. Lee, D. Okamoto, L. Vigneault & A. Galloway |  | PQ883016 |  | PQ888730 |  |  |
| *Boreolithothamnion astragaloi* | GWS049817 | Subtidal (10.4 m), rhodolith, Tanu I., British Columbia, Canada, 52.76233 -131.61822, 23.7.2023, L. Lee, D. Okamoto, L. Vigneault & A. Galloway |  |  |  | PQ888523 |  |  |
| *Boreolithothamnion astragaloi* | GWS049818 | Subtidal (10.4 m), rhodolith, Tanu I., British Columbia, Canada, 52.76233 -131.61822, 23.7.2023, L. Lee, D. Okamoto, L. Vigneault & A. Galloway |  |  |  | PQ888533 |  |  |
| *Boreolithothamnion astragaloi* | GWS049821 | Subtidal (10.4 m), rhodolith, Tanu I., British Columbia, Canada, 52.76233 -131.61822, 23.7.2023, L. Lee, D. Okamoto, L. Vigneault & A. Galloway |  |  |  | PQ888602 |  |  |
| *Boreolithothamnion astragaloi* | GWS049822 | Subtidal (10.4 m), rhodolith, Tanu I., British Columbia, Canada, 52.76233 -131.61822, 23.7.2023, L. Lee, D. Okamoto, L. Vigneault & A. Galloway |  |  |  | PQ888726 |  |  |
| *Boreolithothamnion astragaloi* | GWS049823 | Subtidal (10.4 m), rhodolith, Tanu I., British Columbia, Canada, 52.76233 -131.61822, 23.7.2023, L. Lee, D. Okamoto, L. Vigneault & A. Galloway |  | PQ882886 |  | PQ888562 |  |  |
| *Boreolithothamnion astragaloi* | GWS049825 | Subtidal (10.4 m), rhodolith, Tanu I., British Columbia, Canada, 52.76233 -131.61822, 23.7.2023, L. Lee, D. Okamoto, L. Vigneault & A. Galloway |  |  |  | PQ888554 |  |  |
| *Boreolithothamnion astragaloi* | GWS049826 | Subtidal (10.4 m), rhodolith, Tanu I., British Columbia, Canada, 52.76233 -131.61822, 23.7.2023, L. Lee, D. Okamoto, L. Vigneault & A. Galloway |  |  |  | PQ888720 |  |  |
| *Boreolithothamnion astragaloi* | GWS049827 | Subtidal (10.4 m), rhodolith, Tanu I., British Columbia, Canada, 52.76233 -131.61822, 23.7.2023, L. Lee, D. Okamoto, L. Vigneault & A. Galloway |  |  |  | PQ888450 |  |  |
| *Boreolithothamnion astragaloi* | GWS049828 | Subtidal (10.4 m), rhodolith, Tanu I., British Columbia, Canada, 52.76233 -131.61822, 23.7.2023, L. Lee, D. Okamoto, L. Vigneault & A. Galloway |  |  |  | PQ888596 |  |  |
| *Boreolithothamnion astragaloi* | GWS049829 | Subtidal (10.4 m), rhodolith, Tanu I., British Columbia, Canada, 52.76233 -131.61822, 23.7.2023, L. Lee, D. Okamoto, L. Vigneault & A. Galloway |  | PQ882928 |  | PQ888595 |  |  |
| *Boreolithothamnion astragaloi* | GWS049830 | Subtidal (10.4 m), rhodolith, Tanu I., British Columbia, Canada, 52.76233 -131.61822, 23.7.2023, L. Lee, D. Okamoto, L. Vigneault & A. Galloway |  | PQ882953 |  | PQ888634 |  |  |
| *Boreolithothamnion astragaloi* | GWS049831 | Subtidal (10.4 m), rhodolith, Tanu I., British Columbia, Canada, 52.76233 -131.61822, 23.7.2023, L. Lee, D. Okamoto, L. Vigneault & A. Galloway |  | PQ882804 |  | PQ888455 |  |  |
| *Boreolithothamnion astragaloi* | GWS049832 | Subtidal (10.4 m), rhodolith, Tanu I., British Columbia, Canada, 52.76233 -131.61822, 23.7.2023, L. Lee, D. Okamoto, L. Vigneault & A. Galloway |  | PQ882980 |  | PQ888668 |  |  |
| *Boreolithothamnion astragaloi* | GWS049833 | Subtidal (10.4 m), rhodolith, Tanu I., British Columbia, Canada, 52.76233 -131.61822, 23.7.2023, L. Lee, D. Okamoto, L. Vigneault & A. Galloway |  |  |  | PQ888637 |  |  |
| *Boreolithothamnion astragaloi* | GWS049834 | Subtidal (10.4 m), rhodolith, Tanu I., British Columbia, Canada, 52.76233 -131.61822, 23.7.2023, L. Lee, D. Okamoto, L. Vigneault & A. Galloway |  |  |  | PQ888520 |  |  |
| *Boreolithothamnion astragaloi* | GWS049835 | Subtidal (10.4 m), rhodolith, Tanu I., British Columbia, Canada, 52.76233 -131.61822, 23.7.2023, L. Lee, D. Okamoto, L. Vigneault & A. Galloway |  |  |  | PQ888632 |  |  |
| *Boreolithothamnion astragaloi* | GWS049837 | Subtidal (10.4 m), rhodolith, Tanu I., British Columbia, Canada, 52.76233 -131.61822, 23.7.2023, L. Lee, D. Okamoto, L. Vigneault & A. Galloway |  |  |  | PQ888534 |  |  |
| *Boreolithothamnion astragaloi* | GWS049838 | Subtidal (10.4 m), rhodolith, Tanu I., British Columbia, Canada, 52.76233 -131.61822, 23.7.2023, L. Lee, D. Okamoto, L. Vigneault & A. Galloway |  | PQ882786 |  | PQ888436 |  |  |
| *Boreolithothamnion astragaloi* | GWS049840 | Subtidal (10.4 m), rhodolith, Tanu I., British Columbia, Canada, 52.76233 -131.61822, 23.7.2023, L. Lee, D. Okamoto, L. Vigneault & A. Galloway |  |  |  | PQ888642 |  |  |
| *Boreolithothamnion astragaloi* | GWS049841 | Subtidal (10.4 m), rhodolith, Tanu I., British Columbia, Canada, 52.76233 -131.61822, 23.7.2023, L. Lee, D. Okamoto, L. Vigneault & A. Galloway |  |  |  | PQ888639 |  |  |
| *Boreolithothamnion astragaloi* | GWS049843 | Subtidal (10.4 m), rhodolith, Tanu I., British Columbia, Canada, 52.76233 -131.61822, 23.7.2023, L. Lee, D. Okamoto, L. Vigneault & A. Galloway |  |  |  | PQ888559 |  |  |
| *Boreolithothamnion astragaloi* | GWS049844 | Subtidal (10.4 m), rhodolith, Tanu I., British Columbia, Canada, 52.76233 -131.61822, 23.7.2023, L. Lee, D. Okamoto, L. Vigneault & A. Galloway |  |  |  | PQ888496 |  |  |
| *Boreolithothamnion astragaloi* | GWS049847 | Subtidal (10.4 m), rhodolith, Tanu I., British Columbia, Canada, 52.76233 -131.61822, 23.7.2023, L. Lee, D. Okamoto, L. Vigneault & A. Galloway |  |  |  | PQ888486 |  |  |
| *Boreolithothamnion astragaloi* | GWS049848 | Subtidal (10.4 m), rhodolith, Tanu I., British Columbia, Canada, 52.76233 -131.61822, 23.7.2023, L. Lee, D. Okamoto, L. Vigneault & A. Galloway |  |  |  | PQ888500 |  |  |
| *Boreolithothamnion astragaloi* | GWS049849 | Subtidal (10.4 m), rhodolith, Tanu I., British Columbia, Canada, 52.76233 -131.61822, 23.7.2023, L. Lee, D. Okamoto, L. Vigneault & A. Galloway |  |  |  | PQ888461 |  |  |
| *Boreolithothamnion astragaloi* | GWS049850 | Subtidal (10.4 m), rhodolith, Tanu I., British Columbia, Canada, 52.76233 -131.61822, 23.7.2023, L. Lee, D. Okamoto, L. Vigneault & A. Galloway |  |  |  | PQ888601 |  |  |
| *Boreolithothamnion astragaloi* | GWS049851 | Subtidal (10.4 m), rhodolith, Tanu I., British Columbia, Canada, 52.76233 -131.61822, 23.7.2023, L. Lee, D. Okamoto, L. Vigneault & A. Galloway |  |  |  | PQ888527 |  |  |
| *Boreolithothamnion astragaloi* | GWS049852 | Subtidal (10.4 m), rhodolith, Tanu I., British Columbia, Canada, 52.76233 -131.61822, 23.7.2023, L. Lee, D. Okamoto, L. Vigneault & A. Galloway |  |  |  | PQ888538 |  |  |
| *Boreolithothamnion astragaloi* | GWS049855 | Subtidal (6.5 m), rhodolith, Stansung I., British Columbia, Canada, 52.73877 -131.61853, 23.7.2023, L. Lee, D. Okamoto, L. Vigneault & A. Galloway |  | PQ882853 |  | PQ888512 |  |  |
| *Boreolithothamnion astragaloi* | GWS049858 | Subtidal (6.5 m), rhodolith, Stansung I., British Columbia, Canada, 52.73877 -131.61853, 23.7.2023, L. Lee, D. Okamoto, L. Vigneault & A. Galloway |  | PQ883015 |  | PQ888729 |  |  |
| *Boreolithothamnion glaciale* | GWS002312 | Subtidal (10 m) surrounding rock, crust, inside Guyan I., Cape Breton, Nova Scotia, Canada, 45.7703 -60.1119, 30.8.2004, G.W. Saunders |  |  |  |  |  |  |
| *Boreolithothamnion glaciale* | GWS003728 | Subtidal (5 m) on rock, crust, Pettes Cove, Grand Manan, New Brunswick, Canada, 44.7633 -66.7388, 26.5.2006, G.W. Saunders, B. Clarkston & D. McDevit | HM918602 |  |  |  |  |  |
| *Boreolithothamnion glaciale* | GWS003729 | Subtidal (5 m) on rock, crust, Pettes Cove, Grand Manan, New Brunswick, Canada, 44.7633 -66.7388, 26.5.2006, G.W. Saunders, B. Clarkston & D. McDevit | MH309085 |  |  |  |  |  |
| *Boreolithothamnion glaciale* | GWS005558 | Low intertidal pool on rock, crust, Wreck of the Ithaca, east of Churchill, Manitoba, Canada, 58.7678 -93.8897, 11.7.2007, G.W. Saunders, B. Clarkston, D. McDevit & K. Roy | HM918759 |  |  |  |  |  |
| *Boreolithothamnion glaciale* | GWS005773 | Subtidal (10 m) on cobble, crust, Simpson I., Bay of Fundy, New Brunswick, Canada, 45.0034 -66.9129, 14.8.2007, NAGISA crew 2007 |  | PQ882868 |  |  |  |  |
| *Boreolithothamnion glaciale* | GWS007033 | , crust, Norris Point, Bonne Bay Marine Station, Newfoundland and Labrador, Canada, 49.5178 -57.8739, 11.7.2006, L. Le Gall & J. Utge |  | MW536926 |  |  |  |  |
| *Boreolithothamnion glaciale* | GWS007036 | , crust, Norris Point, Bonne Bay Marine Station, Newfoundland and Labrador, Canada, 49.5178 -57.8739, 11.7.2006, L. Le Gall & J. Utge |  | PQ882943 |  |  |  |  |
| *Boreolithothamnion glaciale* | GWS007037 | , crust, Norris Point, Bonne Bay Marine Station, Newfoundland and Labrador, Canada, 49.5178 -57.8739, 11.7.2006, L. Le Gall & J. Utge |  | MW536877 |  |  |  |  |
| *Boreolithothamnion glaciale* | GWS007311 | Subtidal (18 m) on rock, rhodolith, Maerl bed (btw Deer Arm & East Arm), Bonne Bay, Newfoundland and Labrador, Canada, 49.525278 -57.825556, 13.7.2006, L. Le Gall, B. Hooper & J. Utge |  | MW536863 |  |  |  |  |
| *Boreolithothamnion glaciale* | GWS007312 | Subtidal (18 m) on rock, rhodolith, Maerl bed (btw Deer Arm & East Arm), Bonne Bay, Newfoundland and Labrador, Canada, 49.525278 -57.825556, 13.7.2006, L. Le Gall, B. Hooper & J. Utge | HM918805 |  | KC134336 | KP224290 | KC157593 | PQ137890 |
| *Boreolithothamnion glaciale* | GWS007313 | Subtidal (18 m) on rock, rhodolith, Maerl bed (btw Deer Arm & East Arm), Bonne Bay, Newfoundland and Labrador, Canada, 49.525278 -57.825556, 13.7.2006, L. Le Gall, B. Hooper & J. Utge |  |  |  |  |  |  |
| *Boreolithothamnion glaciale* | GWS007316 | Subtidal (16 m) on rock, crust, Maerl bed (btw Deer Arm & East Arm), Bonne Bay, Newfoundland and Labrador, Canada, 49.525278 -57.825556, 13.7.2006, L. Le Gall, B. Hooper & J. Utge |  | MW536908 |  |  |  |  |
| *Boreolithothamnion glaciale* | GWS007317 | Subtidal (16 m) on rock, crust, Maerl bed (btw Deer Arm & East Arm), Bonne Bay, Newfoundland and Labrador, Canada, 49.525278 -57.825556, 13.7.2006, L. Le Gall, B. Hooper & J. Utge | HM918806 |  |  |  |  |  |
| *Boreolithothamnion glaciale* | GWS007321 | Subtidal (12 m) on rock, crust, Maerl bed (btw Deer Arm & East Arm), Bonne Bay, Newfoundland and Labrador, Canada, 49.525278 -57.825556, 13.7.2006, L. Le Gall, B. Hooper & J. Utge |  | MW536928 |  |  |  |  |
| *Boreolithothamnion glaciale* | GWS007322 | Subtidal (12 m) on rock, rhodolith, Maerl bed (btw Deer Arm & East Arm), Bonne Bay, Newfoundland and Labrador, Canada, 49.525278 -57.825556, 13.7.2006, L. Le Gall, B. Hooper & J. Utge | HM918807 |  |  |  |  |  |
| *Boreolithothamnion glaciale* | GWS007323 | Subtidal (12 m) on rock, crust, Maerl bed (btw Deer Arm & East Arm), Bonne Bay, Newfoundland and Labrador, Canada, 49.525278 -57.825556, 13.7.2006, L. Le Gall, B. Hooper & J. Utge | HM918808 |  |  |  |  |  |
| *Boreolithothamnion glaciale* | GWS007324 | Subtidal (12 m) on rock, crust, Maerl bed (btw Deer Arm & East Arm), Bonne Bay, Newfoundland and Labrador, Canada, 49.525278 -57.825556, 13.7.2006, L. Le Gall, B. Hooper & J. Utge |  | MW536876 |  |  |  |  |
| *Boreolithothamnion glaciale* | GWS007325 | Subtidal (12 m) on shell, crust, Maerl bed (btw Deer Arm & East Arm), Bonne Bay, Newfoundland and Labrador, Canada, 49.525278 -57.825556, 13.7.2006, L. Le Gall, B. Hooper & J. Utge | MH309483 |  |  |  |  |  |
| *Boreolithothamnion glaciale* | GWS007326 | Subtidal (28 m) on rock, crust, Maerl bed (btw Deer Arm & East Arm), Bonne Bay, Newfoundland and Labrador, Canada, 49.525278 -57.825556, 13.7.2006, L. Le Gall, B. Hooper & J. Utge |  | MW536890 |  |  |  |  |
| *Boreolithothamnion glaciale* | GWS007357 | Subtidal on rock, crust, St. Davids, Newfoundland and Labrador, Canada, 48.21857 58.86377, 16.7.2006, L. Le Gall & J. Utge |  | MW536853 |  |  |  |  |
| *Boreolithothamnion glaciale* | GWS007471 | Subtidal on rock, rhodolith, Salvage at Public Oceanview, Newfoundland and Labrador, Canada, 48.6875 -53.6481, 19.7.2006, D. McDevit & J. Utge |  | MW536904 |  |  |  |  |
| *Boreolithothamnion glaciale* | GWS007473 | Subtidal on rock, rhodolith, Salvage at Public Oceanview, Newfoundland and Labrador, Canada, 48.6875 -53.6481, 19.7.2006, D. McDevit & J. Utge |  | MW536903 |  |  |  |  |
| *Boreolithothamnion glaciale* | GWS007474 | Subtidal on rock, crust, Salvage at Public Oceanview, Newfoundland and Labrador, Canada, 48.6875 -53.6481, 19.7.2006, D. McDevit & J. Utge |  | MW536883 |  |  |  |  |
| *Boreolithothamnion glaciale* | GWS007542 | Subtidal (7 m) on rock, crust, English Harbour Eastern Cove, Newfoundland and Labrador, Canada, 47.6331 -54.87, 20.7.2006, L. Le Gall, D. Mc Devit & J. Utge | HM918812 |  |  | JQ422235 |  |  |
| *Boreolithothamnion glaciale* | GWS007668 | Subtidal (40 m) on rock, rhodolith, Dildo, Newfoundland and Labrador, Canada, 47.5708 -53.5556, 25.7.2006, D. McDevit | MH309744 |  |  |  |  |  |
| *Boreolithothamnion glaciale* | GWS007670 | Subtidal (40 m) on rock, rhodolith, Dildo, Newfoundland and Labrador, Canada, 47.5708 -53.5556, 25.7.2006, D. McDevit | MH309967 |  |  |  |  |  |
| *Boreolithothamnion glaciale* | GWS007960 | Subtidal (8 m) on mussels, crust, L’Anse Bleue Breakwater, Northumberland Strait, New Brunswick, Canada, 47.8313 -66.0828, 15.8.2006, L. Le Gall, H. Kucera & J. Utge | HM918813 |  |  |  |  |  |
| *Boreolithothamnion glaciale* | GWS008860 | Subtidal (8 m) on cobble, crust, SE of Beaver Harbour in SCUBA Bay, Bay of Fundy, New Brunswick, Canada, 45.0563 -66.7358, 12.9.2007, G.W. Saunders, K. Dixon & D. McDevit | HM918829 |  |  |  |  |  |
| *Boreolithothamnion glaciale* | GWS008862 | Subtidal (8 m) on cobble, crust, SE of Beaver Harbour in SCUBA Bay, Bay of Fundy, New Brunswick, Canada, 45.0563 -66.7358, 12.9.2007, G.W. Saunders, K. Dixon & D. McDevit | HM918830 |  |  |  |  |  |
| *Boreolithothamnion glaciale* | GWS008867 | Subtidal (8 m) on cobble, crust, SE of Beaver Harbour in SCUBA Bay, Bay of Fundy, New Brunswick, Canada, 45.0563 -66.7358, 12.9.2007, G.W. Saunders, K. Dixon & D. McDevit | HM918831 |  |  |  |  |  |
| *Boreolithothamnion glaciale* | GWS008884 | Lowest intertidal on rock, crust, Letete exposed biodiversity site, Bay of Fundy, New Brunswick, Canada, 45.0382 -66.8912, 25.10.2007, S. Clayden | HM918833 |  |  |  |  |  |
| *Boreolithothamnion glaciale* | GWS008885 | Lowest intertidal on rock, crust, Letete exposed biodiversity site, Bay of Fundy, New Brunswick, Canada, 45.0382 -66.8912, 25.10.2007, S. Clayden | HM918834 |  |  |  |  |  |
| *Boreolithothamnion glaciale* | GWS008887 | Lowest intertidal on rock, crust, Letete exposed biodiversity site, Bay of Fundy, New Brunswick, Canada, 45.0382 -66.8912, 25.10.2007, S. Clayden | HM918835 |  |  |  |  |  |
| *Boreolithothamnion glaciale* | GWS008888 | Lowest intertidal on rock, crust, Letete exposed biodiversity site, Bay of Fundy, New Brunswick, Canada, 45.0382 -66.8912, 25.10.2007, S. Clayden | HM918836 |  |  |  |  |  |
| *Boreolithothamnion glaciale* | GWS008911 | mid intertidal on mussel, crust, Meadow Cove exposed biodiversity site, Bay of Fundy, New Brunswick, Canada, 45.0381 -66.8913, 6.9.2007, K. Hind |  | PQ882894 |  |  |  |  |
| *Boreolithothamnion glaciale* | GWS010989 | Subtidal (10 m) on mussel shell, crust, Eastern Wolf I., New Brunswick, Canada, 44.96953 -66.70811, 21.8.2008, D. McDevit, K. Hind, B. Clarkston, M. Bruce |  | MW536852 |  |  |  |  |
| *Boreolithothamnion glaciale* | GWS010991 | Subtidal (15 m) on mussel shell, crust, Eastern Wolf I., New Brunswick, Canada, 44.96953 -66.70811, 21.8.2008, D. McDevit, K. Hind, B. Clarkston, M. Bruce |  |  |  |  |  |  |
| *Boreolithothamnion glaciale* | GWS010994 | Subtidal (12 m) on rock, crust, Eastern Wolf I., New Brunswick, Canada, 44.96953 -66.70811, 21.8.2008, D. McDevit, K. Hind, B. Clarkston, M. Bruce | HM918954 |  |  |  |  |  |
| *Boreolithothamnion glaciale* | GWS010995 | Subtidal (12 m) on mussel shell, crust, Eastern Wolf I., New Brunswick, Canada, 44.96953 -66.70811, 21.8.2008, D. McDevit, K. Hind, B. Clarkston, M. Bruce | HM918955 |  |  |  |  |  |
| *Boreolithothamnion glaciale* | GWS010996 | Subtidal (12 m) on mussel shell, crust, Eastern Wolf I., New Brunswick, Canada, 44.96953 -66.70811, 21.8.2008, D. McDevit, K. Hind, B. Clarkston, M. Bruce | HM918956 |  |  |  |  |  |
| *Boreolithothamnion glaciale* | GWS010997 | Subtidal (12 m) on rock, crust, Eastern Wolf I., New Brunswick, Canada, 44.96953 -66.70811, 21.8.2008, D. McDevit, K. Hind, B. Clarkston, M. Bruce | HM918957 |  |  |  |  |  |
| *Boreolithothamnion glaciale* | GWS011621 | Subtidal (4 m) on periwinkle, crust, Orby Head, near North Rustico, Prince Edward I., Canada, 46.49058 -63.30533, 28.7.2008, G.W. Saunders & M. Bruce | HM918958 |  |  |  |  |  |
| *Boreolithothamnion glaciale* | GWS011670 | Subtidal (8 m) on rock, crust, Gala Point, East of Charlottetown, Prince Edward I., Canada, 46.11409 -63.00569, 29.7.2008, G.W. Saunders, D. McDevit, S. Hamsher & M. Bruce |  | MW536866 |  |  |  |  |
| *Boreolithothamnion glaciale* | GWS011671 | Subtidal (8 m) on rock, crust, Gala Point, East of Charlottetown, Prince Edward I., Canada, 46.11409 -63.00569, 29.7.2008, G.W. Saunders, D. McDevit, S. Hamsher & M. Bruce | HM918959 |  |  |  |  |  |
| *Boreolithothamnion glaciale* | GWS011710 | Subtidal (10 m) on cobble, crust, Peggys Cove, Nova Scotia, Canada, 44.4905 -63.9166, 31.7.2008, G.W. Saunders, D. McDevit, S. Hamsher & M. Bruce | MH309454 |  |  |  |  |  |
| *Boreolithothamnion glaciale* | GWS011750 | Subtidal (12 m) on rock, crust, Eastern Wolf I., New Brunswick, Canada, 44.96953 -66.70811, 21.8.2008, D. McDevit, K. Hind, B. Clarkston, M. Bruce |  |  |  |  |  |  |
| *Boreolithothamnion glaciale* | GWS011751 | Subtidal (12 m) on rock, crust, Eastern Wolf I., New Brunswick, Canada, 44.96953 -66.70811, 21.8.2008, D. McDevit, K. Hind, B. Clarkston, M. Bruce | HM918961 |  |  |  |  |  |
| *Boreolithothamnion glaciale* | GWS011755 | Subtidal (12 m) on rock, crust, Eastern Wolf I., New Brunswick, Canada, 44.96953 -66.70811, 21.8.2008, D. McDevit, K. Hind, B. Clarkston, M. Bruce |  |  |  |  |  |  |
| *Boreolithothamnion glaciale* | GWS011759 | Subtidal (12 m) on glass, crust, Eastern Wolf I., New Brunswick, Canada, 44.96953 -66.70811, 21.8.2008, D. McDevit, K. Hind, B. Clarkston, M. Bruce | HM918964 |  |  |  |  |  |
| *Boreolithothamnion glaciale* | GWS011765 | Subtidal (10 m) on rock, crust, Flat Wolf I., New Brunswick, Canada, 44.95447 -66.73139, 21.8.2008, K. Hind & D. McDevit |  | MW536862 |  |  |  |  |
| *Boreolithothamnion glaciale* | GWS011766 | Subtidal (10 m) on mussel shell, crust, Flat Wolf I., New Brunswick, Canada, 44.95447 -66.73139, 21.8.2008, K. Hind & D. McDevit | HM918970 |  |  |  |  |  |
| *Boreolithothamnion glaciale* | GWS011767 | Subtidal (10 m) on rock, crust, Flat Wolf I., New Brunswick, Canada, 44.95447 -66.73139, 21.8.2008, K. Hind & D. McDevit |  | PQ882869 |  |  |  |  |
| *Boreolithothamnion glaciale* | GWS011768 | Subtidal (10 m) on rock, crust, Flat Wolf I., New Brunswick, Canada, 44.95447 -66.73139, 21.8.2008, K. Hind & D. McDevit | HM918971 |  |  |  |  |  |
| *Boreolithothamnion glaciale* | GWS011769 | Subtidal (10 m) on rock, crust, Flat Wolf I., New Brunswick, Canada, 44.95447 -66.73139, 21.8.2008, K. Hind & D. McDevit |  |  |  |  |  |  |
| *Boreolithothamnion glaciale* | GWS011773 | Subtidal (8 m) on rock, crust, Flat Wolf I., New Brunswick, Canada, 44.95447 -66.73139, 21.8.2008, B. Clarkston & M. Bruce | HM918973 |  |  |  |  |  |
| *Boreolithothamnion glaciale* | GWS011774 | Subtidal (8 m) on rock, crust, Flat Wolf I., New Brunswick, Canada, 44.95447 -66.73139, 21.8.2008, B. Clarkston & M. Bruce |  |  |  |  |  |  |
| *Boreolithothamnion glaciale* | GWS011835 | Subtidal (4 m) on rock, crust, Paddys Head, Nova Scotia, Canada, 44.5261 -63.947383, 18.5.2009, G.W. Saunders & D. McDevit | HM918976 |  |  |  |  |  |
| *Boreolithothamnion glaciale* | GWS011847 | Subtidal (3 m) on mussel shell, crust, Paddys Head, Nova Scotia, Canada, 44.5261 -63.947383, 18.5.2009, G.W. Saunders & D. McDevit | HM918977 | PQ882808 |  | PQ888468 |  | PQ883097 |
| *Boreolithothamnion glaciale* | GWS017808 | Subtidal (8 m) on rock, crust, Folly Cove, Gloucester left side, Massachusetts, United States of America, 42.68502 -70.64148, 13.4.2010, D. McDevit, M. Bruce |  | MW536873 |  |  |  |  |
| *Boreolithothamnion glaciale* | GWS017813 | Subtidal (5 m) on mussel, crust, Folly Cove, Gloucester, right side, Massachusetts, United States of America, 42.68502 -70.64148, 13.4.2010, B. Clarkston, A. Savoie | HM915082 |  |  |  |  |  |
| *Boreolithothamnion glaciale* | GWS017815 | Subtidal (5 m) on mussel, crust, Folly Cove, Gloucester, right side, Massachusetts, United States of America, 42.68502 -70.64148, 13.4.2010, B. Clarkston, A. Savoie | HM915084 |  |  |  |  |  |
| *Boreolithothamnion glaciale* | GWS017827 | Subtidal (5 m) on mussel, crust, Folly Cove, Gloucester, right side, Massachusetts, United States of America, 42.68502 -70.64148, 13.4.2010, B. Clarkston, A. Savoie | HM915092 |  |  |  |  |  |
| *Boreolithothamnion glaciale* | GWS018163 | Subtidal (3 m) on mussel, crust, Two Lights, Cape Elizabeth, Maine, United States of America, 43.56481 -70.19865, 19.4.2010, B. Clarkston, D. McDevit, M. Bruce, A. Savoie, C. Longtin | HM915216 |  |  |  |  |  |
| *Boreolithothamnion glaciale* | GWS018166 | Subtidal (3 m) on rock, crust, Two Lights, Cape Elizabeth, Maine, United States of America, 43.56481 -70.19865, 19.4.2010, B. Clarkston, D. McDevit, M. Bruce, A. Savoie, C. Longtin | MH309044 |  |  |  |  |  |
| *Boreolithothamnion glaciale* | GWS024058 | Subtidal (5 m) on cobble, crust, Sargeant Point, Somesville, Maine, United States of America, 44.33623 -68.30515, 30.9.2011, G.W. Saunders, A. Savoie & M. Bruce |  | MW536869 |  |  |  |  |
| *Boreolithothamnion glaciale* | GWS024081 | Subtidal (4 m) on mussel, crust, Jasper Beach, Maine, United States of America, 44.62761 -67.38789, 2.10.2011, G.W. Saunders |  | MW536894 |  |  |  |  |
| *Boreolithothamnion glaciale* | GWS029946 | Mid intertidal on rock, crust, Letete exposed biodiversity site, Bay of Fundy, New Brunswick, Canada, 45.0382 -66.8912, 14.9.2011, A. Foorgie | MH309086 |  |  |  |  |  |
| *Boreolithothamnion glaciale* | GWS032015 | Subtidal (3 m) on pebble, crust, Covey Head, Mahone Bay, Nova Scotia, Canada, 44.50839 -64.12634, 13.8.2012, G.W. Saunders & A. Savoie | MH308938 |  |  |  |  |  |
| *Boreolithothamnion glaciale* | GWS039570 | Subtidal (6 m) on rock, crust, New River Beach, Bay of Fundy, New Brunswick, Canada, 45.120012 -66.524708, 28.6.2015, M. Bruce |  | PQ882820 |  |  |  |  |
| *Boreolithothamnion glaciale* | GWS040334 | Subtidal (2 m), rhodolith, Turnagain I., outside village Black Harbour, Labrador, Newfoundland and Labrador, Canada, 56.76594 -61.31059, 9.9.2014, G.W. Saunders, K. Dixon & C. Lane |  | MW536842 |  |  |  |  |
| *Boreolithothamnion glaciale* | GWS040341 | Subtidal (10 m) on bottom, rhodolith, Turnagain I., outside village Black Harbour, Labrador, Newfoundland and Labrador, Canada, 56.76594 -61.31059, 9.9.2014, G.W. Saunders, K. Dixon & C. Lane |  | MW536915 |  |  |  |  |
| *Boreolithothamnion glaciale* | GWS040342 | Subtidal (10 m) on bottom, rhodolith, Turnagain I., outside village Black Harbour, Labrador, Newfoundland and Labrador, Canada, 56.76594 -61.31059, 9.9.2014, G.W. Saunders, K. Dixon & C. Lane |  | MW536867 |  |  |  |  |
| *Boreolithothamnion glaciale* | GWS040345 | Subtidal (10 m) on shell, crust, Turnagain I., outside village Black Harbour, Labrador, Newfoundland and Labrador, Canada, 56.76594 -61.31059, 9.9.2014, G.W. Saunders, K. Dixon & C. Lane |  | MW536889 |  |  |  |  |
| *Boreolithothamnion glaciale* | GWS040347 | Subtidal (10 m) on cobble, crust, Turnagain I., outside village Black Harbour, Labrador, Newfoundland and Labrador, Canada, 56.76594 -61.31059, 9.9.2014, G.W. Saunders, K. Dixon & C. Lane |  | MW536864 |  |  |  |  |
| *Boreolithothamnion glaciale* | GWS040356 | Subtidal (12 m) on barnacle test, crust, Makkovic Harbour, Labrador, Newfoundland and Labrador, Canada, 55.08429 -59.16566, 10.9.2014, G.W. Saunders & K. Dixon |  | MW536850 |  |  |  |  |
| *Boreolithothamnion glaciale* | GWS040359 | Subtidal (6 m) on rock, crust, Point NW of Lighthouse, Makkovic, Labrador, Newfoundland and Labrador, Canada, 55.10165 -59.18001, 11.9.2014, G.W. Saunders, K. Dixon & C. Lane |  | PQ882867 |  |  |  |  |
| *Boreolithothamnion glaciale* | GWS040423 | Subtidal (6 m) on rock, rhodolith, Point NW of Lighthouse, Makkovic, Labrador, Newfoundland and Labrador, Canada, 55.10165 -59.18001, 11.9.2014, G.W. Saunders, K. Dixon & C. Lane |  | MW536841 |  |  |  |  |
| *Boreolithothamnion glaciale* | GWS040816 | Subtidal (3 m) on rock, crust, Stora Lauvøyna (at intersection with Fv219), Norway, 60.44247 5.06462, 11.6.2016, G.W. Saunders & T. Bringloe |  |  |  |  |  |  |
| *Boreolithothamnion glaciale* | GWS040840 | Subtidal (14 m) on rock, crust, Stora Lauvøyna (at intersection with Fv219), Norway, 60.44247 5.06462, 11.6.2016, G.W. Saunders & T. Bringloe |  |  |  |  |  |  |
| *Boreolithothamnion glaciale* | GWS040872 | Subtidal (10 m) on shell, crust, Stora Lauvøyna (at intersection with Fv219), Norway, 60.44247 5.06462, 11.6.2016, G.W. Saunders & T. Bringloe |  | PQ882911 |  |  |  |  |
| *Boreolithothamnion glaciale* | GWS041357 | Subtidal (14 m) on cobble, crust, Cable Head, Prince Edward I., Canada, 46.47733 -62.62954, 10.8.2016, G.W. Saunders, T. Bringloe, C. Brooks & A. Savoie | MH309064 |  |  |  |  |  |
| *Boreolithothamnion glaciale* | GWS041463 | Subtidal (22 m) on rock, crust, Naufrage (deep), Prince Edward I., Canada, 46.47441 -62.54773, 11.8.2016, G.W. Saunders, T. Bringloe, C. Brooks & A. Savoie | MH308892 |  |  |  |  |  |
| *Boreolithothamnion glaciale* | GWS041712 | Subtidal (15 m) on cobble, crust, Hospital I., Bay of Fundy, New Brunswick, Canada, 45.11816 -67.01185, 5.6.2017, G.W. Saunders & T. Bringloe |  | PQ882998 |  |  |  |  |
| *Boreolithothamnion glaciale* | GWS041716 | Subtidal (15 m) on cobble, crust, Hospital I., Bay of Fundy, New Brunswick, Canada, 45.11816 -67.01185, 5.6.2017, G.W. Saunders & T. Bringloe |  | PQ882933 |  |  |  |  |
| *Boreolithothamnion glaciale* | GWS041783 | Subtidal (6 m) on cobble, crust, SE of Beaver Harbour in SCUBA Bay, Bay of Fundy, New Brunswick, Canada, 45.0563 -66.7358, 12.7.2017, G.W. Saunders, T. Bringloe, K. Cripps & A. Savoie | MW536941 |  |  |  |  |  |
| *Boreolithothamnion glaciale* | GWS041787 | Subtidal (8 m) on cobble, crust, SE of Beaver Harbour in SCUBA Bay, Bay of Fundy, New Brunswick, Canada, 45.0563 -66.7358, 12.7.2017, G.W. Saunders, T. Bringloe, K. Cripps & A. Savoie |  | PQ882888 |  |  |  |  |
| *Boreolithothamnion glaciale* | GWS041788 | Subtidal (8 m) on cobble, crust, SE of Beaver Harbour in SCUBA Bay, Bay of Fundy, New Brunswick, Canada, 45.0563 -66.7358, 12.7.2017, G.W. Saunders, T. Bringloe, K. Cripps & A. Savoie |  | PQ882837 |  |  |  |  |
| *Boreolithothamnion glaciale* | GWS041920 | Subtidal (8 m) on cobble, crust, Nome, Sledge I. East#1, Alaska, United States of America, 64.48148 -166.19455, 15.8.2017, G.W. Saunders & T. Bringloe | MH309050 |  |  |  |  |  |
| *Boreolithothamnion glaciale* | GWS041921 | Subtidal (8 m) on cobble, crust, Nome, Sledge I. East#1, Alaska, United States of America, 64.48148 -166.19455, 15.8.2017, G.W. Saunders & T. Bringloe | MH308797 |  |  |  |  |  |
| *Boreolithothamnion glaciale* | GWS041922 | Subtidal (8 m) on cobble, crust, Nome, Sledge I. East#1, Alaska, United States of America, 64.48148 -166.19455, 15.8.2017, G.W. Saunders & T. Bringloe | MH309499 |  |  |  |  |  |
| *Boreolithothamnion glaciale* | GWS041987 | Subtidal (5 m) on cobble, crust, Nome, Sledge I. East#2, Alaska, United States of America, 64.48737 -166.19298, 15.8.2017, G.W. Saunders & T. Bringloe | MH309402 |  |  |  |  |  |
| *Boreolithothamnion glaciale* | GWS042690 | Subtidal (35.69 m) on rock, crust, Cruise HUD2018021, Nova Scotia, Canada, 44.71382 -62.660743, 5.7.2018, H. Vandermeulin |  | PQ882847 |  |  |  |  |
| *Boreolithothamnion glaciale* | GWS042691 | Subtidal (35.69 m) on rock, crust, Cruise HUD2018021, Nova Scotia, Canada, 44.71382 -62.660743, 5.7.2018, H. Vandermeulin |  | PQ882822 |  |  |  |  |
| *Boreolithothamnion glaciale* | GWS042693 | Subtidal (35.99 m) on rock, crust, Cruise HUD2018021, Nova Scotia, Canada, 44.713573 -62.660682, 5.7.2018, H. Vandermeulin |  | PQ882832 |  |  |  |  |
| *Boreolithothamnion glaciale* | GWS043057 | Mid intertidal pool on rock, crust, Mispec Beach, Saint John Harbour monitoring site, New Brunswick, Canada, 45.2175 -65.95274, 17.9.2020, G.W. Saunders & C. Brooks |  | PQ882793 |  |  |  |  |
| *Boreolithothamnion glaciale* | GWS044336 | Subtidal (10 m) on cobble, crust, Deep Cove Point, New River Beach, Bay of Fundy, New Brunswick, Canada, 45.116645 -66.525, 25.8.2020, G.W. Saunders & C. Brooks |  | PQ882957 |  |  |  |  |
| *Boreolithothamnion glaciale* | GWS044337 | Subtidal (10 m) on cobble, crust, Deep Cove Point, New River Beach, Bay of Fundy, New Brunswick, Canada, 45.116645 -66.525, 25.8.2020, G.W. Saunders & C. Brooks |  | PQ882860 |  |  |  |  |
| *Boreolithothamnion glaciale* | GWS044338 | Subtidal (10 m) on cobble, crust, Deep Cove Point, New River Beach, Bay of Fundy, New Brunswick, Canada, 45.116645 -66.525, 25.8.2020, G.W. Saunders & C. Brooks |  | PQ882918 |  |  |  |  |
| *Boreolithothamnion glaciale* | GWS044339 | Subtidal (10 m) on cobble, crust, Deep Cove Point, New River Beach, Bay of Fundy, New Brunswick, Canada, 45.116645 -66.525, 25.8.2020, G.W. Saunders & C. Brooks |  | PQ883009 |  |  |  |  |
| *Boreolithothamnion glaciale* | GWS044352 | Subtidal (5 m) on mussel shell, crust, Deep Cove Point, New River Beach, Bay of Fundy, New Brunswick, Canada, 45.116645 -66.525, 25.8.2020, G.W. Saunders & C. Brooks |  | PQ882946 |  |  |  |  |
| *Boreolithothamnion glaciale* | GWS044357 | Subtidal (2 m) on rock, crust, Deep Cove Point, New River Beach, Bay of Fundy, New Brunswick, Canada, 45.116645 -66.525, 25.8.2020, G.W. Saunders & C. Brooks |  | PQ882985 |  |  |  |  |
| *Boreolithothamnion glaciale* | GWS044368 | Subtidal (8 m) on rock, crust, Seeleys Cove, Bay of Fundy, New Brunswick, Canada, 45.088733 -66.642949, 25.8.2020, G.W. Saunders & C. Brooks |  | PQ882913 |  |  |  |  |
| *Boreolithothamnion glaciale* | GWS044373 | Subtidal (2 m) on cobble, crust, Seeleys Cove, Bay of Fundy, New Brunswick, Canada, 45.088733 -66.642949, 25.8.2020, G.W. Saunders & C. Brooks |  | PQ883002 |  |  |  |  |
| *Boreolithothamnion glaciale* | GWS044406 | Subtidal (2 m) on rock, crust, Duck Pond Beach (rocks west), New Brunswick, Canada, 45.28011 -65.6924, 7.8.2020, G.W. Saunders & C. Brooks |  |  |  |  |  |  |
| *Boreolithothamnion glaciale* | GWS045033 | Subtidal (9 m) on mussel, crust, Murphy’s Ledge, Bliss I., Bay of Fundy, New Brunswick, Canada, 45.031113 -66.82223, 26.8.2020, G.W. Saunders & C. Brooks |  | PQ882993 |  |  |  |  |
| *Boreolithothamnion glaciale* | GWS045034 | Subtidal (9 m) on mussel, crust, Murphy’s Ledge, Bliss I., Bay of Fundy, New Brunswick, Canada, 45.031113 -66.82223, 26.8.2020, G.W. Saunders & C. Brooks |  | PQ882955 |  |  |  |  |
| *Boreolithothamnion glaciale* | GWS045044 | Subtidal (5 m) on mussel, crust, Murphy’s Ledge, Bliss I., Bay of Fundy, New Brunswick, Canada, 45.031113 -66.82223, 26.8.2020, G.W. Saunders & C. Brooks |  | PQ882987 |  |  |  |  |
| *Boreolithothamnion glaciale* | GWS045850 | Mid intertidal pool on rock, crust, Lepreau exposed biodiversity site, Bay of Fundy, New Brunswick, Canada, 45.0722 -66.469, 7.7.2020, G.W. Saunders, C. Brooks & M. Dankworth |  | PQ882924 |  |  |  |  |
| *Boreolithothamnion glaciale* | GWS045879 | Subtidal (5 m) on rock, crust, Pork Ledge, Saint John Harbour monitoring site, New Brunswick, Canada, 45.130054 -66.306438, 29.10.2020, G.W. Saunders & C. Brooks |  | PQ883013 |  |  |  |  |
| *Boreolithothamnion glaciale* | GWS046585 | Subtidal (2 m) on mussel, crust, Pork Ledge, Saint John Harbour monitoring site, New Brunswick, Canada, 45.130054 -66.306438, 29.10.2020, G.W. Saunders & C. Brooks |  | PQ882836 |  |  |  |  |
| *Boreolithothamnion glaciale* | GWS047121 | Low intertidal pool on rock, crust, Anthony Cove Rd., Saint John Harbour monitoring site, New Brunswick, Canada, 45.22533 -65.01115, 24.5.2021, G.W. Saunders & C. Brooks |  |  |  |  |  |  |
| *Boreolithothamnion glaciale* | GWS047216 | Upper intertidal pool on rock, crust, Letete exposed biodiversity site, Bay of Fundy, New Brunswick, Canada, 45.0382 -66.8912, 26.6.2021, G.W. Saunders & J. Crichton |  |  |  |  |  |  |
| *Boreolithothamnion glaciale* | GWS047217 | Upper intertidal pool on rock, crust, Letete exposed biodiversity site, Bay of Fundy, New Brunswick, Canada, 45.0382 -66.8912, 26.6.2021, G.W. Saunders & J. Crichton |  | PQ882788 |  |  |  |  |
| *Boreolithothamnion lemoineae* | GWS040223 | Subtidal (5 m) on shell, crust, Point in Durban Harbour, Nunavut, Canada, 67.03925 -62.22638, 3.9.2014, G.W. Saunders, K. Dixon & C. Lane | MH309918 |  |  |  | PQ883079 |  |
| *Boreolithothamnion lemoineae* | GWS040335 | Subtidal (10 m) on bottom, crust, Turnagain I., outside village Black Harbour, Labrador, Newfoundland and Labrador, Canada, 56.76594 -61.31059, 9.9.2014, G.W. Saunders, K. Dixon & C. Lane | MH308969 |  | MH277192 | MW536564 |  |  |
| *Boreolithothamnion phymatodeum* | GWS009940 | Low intertidal pool on rock, crust, Tahsis, I. #40 on Esperenza Inlet Chart, British Columbia, Canada, 49.8125 -126.98733, 20.5.2008, K. Hind & D. McDevit | HM918922 |  |  |  |  |  |
| *Boreolithothamnion phymatodeum* | GWS010031 | Mid intertidal on rock, sheltered, crust, Tahsis, I. #40 on Esperenza Inlet Chart, British Columbia, Canada, 49.8125 -126.98733, 21.5.2008, K. Hind & D. McDevit | HM918924 |  |  |  |  |  |
| *Boreolithothamnion phymatodeum* | GWS010543 | Subtidal (6 m) on rock, crust, Bamfield, Dixon I. (up-channel bay), British Columbia, Canada, 48.85428 -125.13667, 2.6.2008, K. Hind & D. McDevit | MW536965 |  |  |  |  |  |
| *Boreolithothamnion phymatodeum* | GWS013482 | Low intertidal in channel on cobble, crust, Burnaby (Dolomite) Narrows, Gwaii Haanas, British Columbia, Canada, 52.35993 -131.35201, 23.6.2009, G.W. Saunders & D. McDevit | HM916690 |  |  |  |  |  |
| *Boreolithothamnion phymatodeum* | GWS019231 | Low intertidal on cobble, crust, Bamfield, Wizard I., British Columbia, Canada, 48.8583 -125.1588, 28.5.2010, G.W. Saunders & K. Dixon | MW536989 |  |  |  |  |  |
| *Boreolithothamnion phymatodeum* | GWS019298 | Low intertidal on rock, crust, Bamfield, Blowhole at Brady’s Beach, British Columbia, Canada, 48.8241 -125.162, 29.5.2010, G.W. Saunders & K. Dixon | HQ544314 |  |  |  |  |  |
| *Boreolithothamnion phymatodeum* | GWS019930 | Low intertidal pool on rock, semi-exposed, crust, Koga Islet, Gwaii Haanas, British Columbia, Canada, 52.42831 -131.3786, 12.6.2010, G.W. Saunders & K. Dixon | MW536960 |  |  |  |  |  |
| *Boreolithothamnion phymatodeum* | GWS020428 | Low intertidal on pebble sheltered, crust, Hotspring I. (east 'back' side), Gwaii Haanas, British Columbia, Canada, 52.5779 -131.43768, 15.6.2010, G.W. Saunders & K. Dixon | HQ544911 |  |  |  |  |  |
| *Boreolithothamnion phymatodeum* | GWS020565 | Low intertidal on cobble sheltered, crust, Tanu I. (Watchmen Station), Haida Gwaii, British Columbia, Canada, 52.76248 -131.61214, 16.6.2010, G.W. Saunders & K. Dixon | MW536972 |  |  |  |  |  |
| *Boreolithothamnion phymatodeum* | GWS021030 | Subtidal (6 m) on rock, crust, Between Wiah Point & Cape Edenshaw (#2), NW of Masset, Haida Gwaii, British Columbia, Canada, 54.10699 -132.36641, 8.6.2010, G.W. Saunders & K. Dixon | MW536939 |  |  |  |  |  |
| *Boreolithothamnion phymatodeum* | GWS021409 | Low intertidal on rock, crust, Pigeon Point Lighthouse, California, United States of America, 37.18316 -122.38873, 15.5.2010, B. Clarkston & K. Hind | KM254263 |  |  |  |  |  |
| *Boreolithothamnion phymatodeum* | GWS028193 | Subtidal (5 m) on shell, crust, Murchison I., Northwest Beach, Gwaii Haanas, British Columbia, Canada, 52.60787 -131.4482, 7.7.2011, G.W. Saunders & K. Dixon | MW536963 | PQ882896 |  | PQ888572 |  | PQ883115 |
| *Boreolithothamnion phymatodeum* | GWS031009 | Subtidal (5 m) on cobble, crust, Tanu I., Gwaii Haanas, British Columbia, Canada, 52.76458 -131.61066, 14.6.2012, G.W. Saunders & K. Dixon | MW536946 |  | PQ882904 |  |  |  |
| *Boreolithothamnion phymatodeum* | GWS046790 | Subtidal (5 m) on rock, crust, Faraday I., S Site, Plot 2 (5-10 ft), Gwaii Haanas, British Columbia, Canada, 52.60913 -131.46582, 27.7.2022, G.W. Saunders & C. Brooks |  | PQ882908 |  | PQ888581 |  | PQ883118 |
| *Boreolithothamnion phymatodeum* | GWS047938 | Subtidal (5-10 ft) on limpet, crust, Faraday I., N Site, Plot 1 (5-10 ft), Gwaii Haanas, British Columbia, Canada, 52.61523 -131.46466, 6.9.2021, G.W. Saunders & C. Brooks |  | PQ882874 |  |  |  |  |
| *Boreolithothamnion phymatodeum* | GWS048021 | Subtidal (2.5 m) on limpet, crust, Faraday I., S Site, Plot 2 (5-10 ft), Gwaii Haanas, British Columbia, Canada, 52.60913 -131.46582, 8.9.2021, G.W. Saunders & C. Brooks |  | PQ882840 |  |  |  |  |
| *Boreolithothamnion phymatodeum* | GWS048024 | Subtidal (2.5 m) on cobble, crust, Faraday I., S Site, Plot 2 (5-10 ft), Gwaii Haanas, British Columbia, Canada, 52.60913 -131.46582, 8.9.2021, G.W. Saunders & C. Brooks |  | PQ882967 |  |  |  |  |
| *Boreolithothamnion phymatodeum* | GWS048041 | Subtidal (1.5 m) on cobble, crust, Faraday I., S Site, Plot 2 (2-5 ft), Gwaii Haanas, British Columbia, Canada, 52.60913 -131.46582, 8.9.2021, G.W. Saunders & C. Brooks |  | PQ882883 |  |  |  |  |
| *Boreolithothamnion phymatodeum* | GWS048273 | Subtidal (6 m) on limpet shell, crust, Gordon I.s (East; 6 m), Gwaii Haanas, British Columbia, Canada, 52.099867 -131.146133, 12.9.2021, G.W. Saunders & C. Brooks |  | PQ882954 |  | PQ888635 |  |  |
| *Boreolithothamnion phymatodeum* | GWS048604 | Subtidal (5 m), rhodolith, Channel btw Murchison & Faraday I., Gwaii Haanas, British Columbia, Canada, 52.59687 -131.47513, 2.8.2022, G.W. Saunders & C. Brooks |  | PQ882851 |  |  |  | PQ883102 |
| *Boreolithothamnion phymatodeum* | GWS048618 | Subtidal (5 m), rhodolith, Channel btw Murchison & Faraday I., Gwaii Haanas, British Columbia, Canada, 52.59687 -131.47513, 2.8.2022, G.W. Saunders & C. Brooks |  | PQ882912 |  |  |  | PQ883119 |
| *Boreolithothamnion phymatodeum* | GWS048619 | Subtidal (5 m), rhodolith, Channel btw Murchison & Faraday I., Gwaii Haanas, British Columbia, Canada, 52.59687 -131.47513, 2.8.2022, G.W. Saunders & C. Brooks |  | PQ882920 |  |  |  |  |
| *Boreolithothamnion phymatodeum* | GWS048620 | Subtidal (5 m), rhodolith, Channel btw Murchison & Faraday I., Gwaii Haanas, British Columbia, Canada, 52.59687 -131.47513, 2.8.2022, G.W. Saunders & C. Brooks |  | PQ882884 |  |  |  | PQ883114 |
| *Boreolithothamnion phymatodeum* | GWS048632 | Subtidal (10 m) on worm tube, crust, Fuller Pt. (Windy Bay), Gwaii Haanas, British Columbia, Canada, 52.69953 -131.44072, 3.8.2022, G.W. Saunders & C. Brooks |  |  |  | PQ888446 | PQ883059 |  |
| *Boreolithothamnion phymatodeum* | GWS049700 | Subtidal (6.5 m), rhodolith, Hotspring I., British Columbia, Canada, 52.57847 -131.44319, 15.7.2023, L. Lee, D. Okamoto, L. Vigneault & A. Galloway |  | PQ882801 |  | PQ888452 |  |  |
| *Boreolithothamnion phymatodeum* | GWS049701 | Subtidal (6.5 m), rhodolith, Hotspring I., British Columbia, Canada, 52.57847 -131.44319, 15.7.2023, L. Lee, D. Okamoto, L. Vigneault & A. Galloway |  | PQ882871 |  | PQ888543 |  |  |
| *Boreolithothamnion phymatodeum* | GWS049702 | Subtidal (6.5 m), rhodolith, Hotspring I., British Columbia, Canada, 52.57847 -131.44319, 15.7.2023, L. Lee, D. Okamoto, L. Vigneault & A. Galloway |  | PQ882790 |  |  |  |  |
| *Boreolithothamnion phymatodeum* | GWS049707 | Subtidal (6.5 m), rhodolith, Hotspring I., British Columbia, Canada, 52.57847 -131.44319, 15.7.2023, L. Lee, D. Okamoto, L. Vigneault & A. Galloway |  |  |  | PQ888688 |  |  |
| *Boreolithothamnion phymatodeum* | GWS049708 | Subtidal (6.5 m), rhodolith, Hotspring I., British Columbia, Canada, 52.57847 -131.44319, 15.7.2023, L. Lee, D. Okamoto, L. Vigneault & A. Galloway |  | PQ882789 |  | PQ888438 |  |  |
| *Boreolithothamnion phymatodeum* | GWS049709 | Subtidal (6.5 m), rhodolith, Hotspring I., British Columbia, Canada, 52.57847 -131.44319, 15.7.2023, L. Lee, D. Okamoto, L. Vigneault & A. Galloway |  |  |  | PQ888683 |  |  |
| *Boreolithothamnion phymatodeum* | GWS049712 | Subtidal (6.5 m), rhodolith, Hotspring I., British Columbia, Canada, 52.57847 -131.44319, 15.7.2023, L. Lee, D. Okamoto, L. Vigneault & A. Galloway |  |  |  | PQ888621 |  |  |
| *Boreolithothamnion phymatodeum* | GWS049714 | Subtidal (6.5 m), rhodolith, Hotspring I., British Columbia, Canada, 52.57847 -131.44319, 15.7.2023, L. Lee, D. Okamoto, L. Vigneault & A. Galloway |  |  |  | PQ888630 |  |  |
| *Boreolithothamnion phymatodeum* | GWS049720 | Subtidal (6.5 m), rhodolith, Hotspring I., British Columbia, Canada, 52.57847 -131.44319, 15.7.2023, L. Lee, D. Okamoto, L. Vigneault & A. Galloway |  |  |  | PQ888516 |  |  |
| *Boreolithothamnion phymatodeum* | GWS049724 | Subtidal (6.5 m), rhodolith, Hotspring I., British Columbia, Canada, 52.57847 -131.44319, 15.7.2023, L. Lee, D. Okamoto, L. Vigneault & A. Galloway |  |  |  | PQ888692 |  |  |
| *Boreolithothamnion phymatodeum* | GWS049726 | Subtidal (6.5 m), rhodolith, Hotspring I., British Columbia, Canada, 52.57847 -131.44319, 15.7.2023, L. Lee, D. Okamoto, L. Vigneault & A. Galloway |  |  |  | PQ888612 |  |  |
| *Boreolithothamnion phymatodeum* | GWS049729 | Subtidal (6.5 m), rhodolith, Hotspring I., British Columbia, Canada, 52.57847 -131.44319, 15.7.2023, L. Lee, D. Okamoto, L. Vigneault & A. Galloway |  | PQ882835 |  | PQ888498 |  |  |
| *Boreolithothamnion phymatodeum* | GWS049732 | Subtidal (6.5 m), rhodolith, Hotspring I., British Columbia, Canada, 52.57847 -131.44319, 15.7.2023, L. Lee, D. Okamoto, L. Vigneault & A. Galloway |  |  |  | PQ888672 |  |  |
| *Boreolithothamnion phymatodeum* | GWS049733 | Subtidal (6.5 m), rhodolith, Hotspring I., British Columbia, Canada, 52.57847 -131.44319, 15.7.2023, L. Lee, D. Okamoto, L. Vigneault & A. Galloway |  |  |  | PQ888603 |  |  |
| *Boreolithothamnion phymatodeum* | GWS049734 | Subtidal (6.5 m), rhodolith, Hotspring I., British Columbia, Canada, 52.57847 -131.44319, 15.7.2023, L. Lee, D. Okamoto, L. Vigneault & A. Galloway |  |  |  | PQ888707 |  |  |
| *Boreolithothamnion phymatodeum* | GWS049736 | Subtidal (6.5 m) on cobble, crust, Hotspring I., British Columbia, Canada, 52.57847 -131.44319, 15.7.2023, L. Lee, D. Okamoto, L. Vigneault & A. Galloway |  |  |  | PQ888478 |  |  |
| *Boreolithothamnion phymatodeum* | GWS049739 | Subtidal (6.5 m), rhodolith, Hotspring I., British Columbia, Canada, 52.57847 -131.44319, 15.7.2023, L. Lee, D. Okamoto, L. Vigneault & A. Galloway |  |  |  | PQ888598 |  |  |
| *Boreolithothamnion phymatodeum* | GWS049741 | Subtidal (6.5 m), rhodolith, Hotspring I., British Columbia, Canada, 52.57847 -131.44319, 15.7.2023, L. Lee, D. Okamoto, L. Vigneault & A. Galloway |  |  |  | PQ888432 |  |  |
| *Boreolithothamnion phymatodeum* | GWS049742 | Subtidal (6.5 m), rhodolith, Hotspring I., British Columbia, Canada, 52.57847 -131.44319, 15.7.2023, L. Lee, D. Okamoto, L. Vigneault & A. Galloway |  |  |  | PQ888701 |  |  |
| *Boreolithothamnion phymatodeum* | GWS049744 | Subtidal (6.5 m), rhodolith, Hotspring I., British Columbia, Canada, 52.57847 -131.44319, 15.7.2023, L. Lee, D. Okamoto, L. Vigneault & A. Galloway |  |  |  | PQ888549 |  |  |
| *Boreolithothamnion phymatodeum* | GWS049746 | Subtidal (6.5 m), rhodolith, Hotspring I., British Columbia, Canada, 52.57847 -131.44319, 15.7.2023, L. Lee, D. Okamoto, L. Vigneault & A. Galloway |  |  |  | PQ888620 |  |  |
| *Boreolithothamnion phymatodeum* | GWS049749 | Subtidal (6.5 m), rhodolith, Hotspring I., British Columbia, Canada, 52.57847 -131.44319, 15.7.2023, L. Lee, D. Okamoto, L. Vigneault & A. Galloway |  |  |  | PQ888663 |  |  |
| *Boreolithothamnion phymatodeum* | GWS049752 | Subtidal (4.6 m), rhodolith, Hotspring I., British Columbia, Canada, 52.57847 -131.44319, 15.7.2023, L. Lee, D. Okamoto, L. Vigneault & A. Galloway |  |  |  | PQ888727 |  |  |
| *Boreolithothamnion phymatodeum* | GWS049756 | Subtidal (4.6 m), rhodolith, Hotspring I., British Columbia, Canada, 52.57847 -131.44319, 15.7.2023, L. Lee, D. Okamoto, L. Vigneault & A. Galloway |  | PQ882887 |  | PQ888564 |  |  |
| *Boreolithothamnion phymatodeum* | GWS049758 | Subtidal (4.6 m), rhodolith, Hotspring I., British Columbia, Canada, 52.57847 -131.44319, 15.7.2023, L. Lee, D. Okamoto, L. Vigneault & A. Galloway |  |  |  | PQ888670 |  |  |
| *Boreolithothamnion phymatodeum* | GWS049759 | Subtidal (4.6 m), rhodolith, Hotspring I., British Columbia, Canada, 52.57847 -131.44319, 15.7.2023, L. Lee, D. Okamoto, L. Vigneault & A. Galloway |  |  |  | PQ888532 |  |  |
| *Boreolithothamnion phymatodeum* | GWS049760 | Subtidal (4.6 m), rhodolith, Hotspring I., British Columbia, Canada, 52.57847 -131.44319, 15.7.2023, L. Lee, D. Okamoto, L. Vigneault & A. Galloway |  |  |  | PQ888718 |  |  |
| *Boreolithothamnion phymatodeum* | GWS049761 | Subtidal (4.6 m), rhodolith, Hotspring I., British Columbia, Canada, 52.57847 -131.44319, 15.7.2023, L. Lee, D. Okamoto, L. Vigneault & A. Galloway |  |  |  | PQ888561 |  |  |
| *Boreolithothamnion phymatodeum* | GWS049762 | Subtidal (4.6 m), rhodolith, Hotspring I., British Columbia, Canada, 52.57847 -131.44319, 15.7.2023, L. Lee, D. Okamoto, L. Vigneault & A. Galloway |  |  |  | PQ888616 |  |  |
| *Boreolithothamnion phymatodeum* | GWS049763 | Subtidal (4.6 m), rhodolith, Hotspring I., British Columbia, Canada, 52.57847 -131.44319, 15.7.2023, L. Lee, D. Okamoto, L. Vigneault & A. Galloway |  |  |  | PQ888650 |  |  |
| *Boreolithothamnion phymatodeum* | GWS049764 | Subtidal (4.6 m), rhodolith, Hotspring I., British Columbia, Canada, 52.57847 -131.44319, 15.7.2023, L. Lee, D. Okamoto, L. Vigneault & A. Galloway |  |  |  | PQ888531 |  |  |
| *Boreolithothamnion phymatodeum* | GWS049765 | Subtidal (4.6 m), rhodolith, Hotspring I., British Columbia, Canada, 52.57847 -131.44319, 15.7.2023, L. Lee, D. Okamoto, L. Vigneault & A. Galloway |  |  |  | PQ888609 |  |  |
| *Boreolithothamnion phymatodeum* | GWS049768 | Subtidal (4.6 m), rhodolith, Hotspring I., British Columbia, Canada, 52.57847 -131.44319, 15.7.2023, L. Lee, D. Okamoto, L. Vigneault & A. Galloway |  |  |  | PQ888725 |  |  |
| *Boreolithothamnion phymatodeum* | GWS049769 | Subtidal (4.6 m), rhodolith, Hotspring I., British Columbia, Canada, 52.57847 -131.44319, 15.7.2023, L. Lee, D. Okamoto, L. Vigneault & A. Galloway |  |  |  | PQ888591 |  |  |
| *Boreolithothamnion phymatodeum* | GWS049770 | Subtidal (4.6 m), rhodolith, Hotspring I., British Columbia, Canada, 52.57847 -131.44319, 15.7.2023, L. Lee, D. Okamoto, L. Vigneault & A. Galloway |  |  |  | PQ888487 |  |  |
| *Boreolithothamnion phymatodeum* | GWS049771 | Subtidal (4.6 m), rhodolith, Hotspring I., British Columbia, Canada, 52.57847 -131.44319, 15.7.2023, L. Lee, D. Okamoto, L. Vigneault & A. Galloway |  |  |  | PQ888584 |  |  |
| *Boreolithothamnion phymatodeum* | GWS049772 | Subtidal (4.6 m), rhodolith, Hotspring I., British Columbia, Canada, 52.57847 -131.44319, 15.7.2023, L. Lee, D. Okamoto, L. Vigneault & A. Galloway |  |  |  | PQ888647 |  |  |
| *Boreolithothamnion phymatodeum* | GWS049781 | Subtidal (4.6 m), rhodolith, Hotspring I., British Columbia, Canada, 52.57847 -131.44319, 15.7.2023, L. Lee, D. Okamoto, L. Vigneault & A. Galloway |  |  |  | PQ888518 |  |  |
| *Boreolithothamnion phymatodeum* | GWS049782 | Subtidal (4.6 m), rhodolith, Hotspring I., British Columbia, Canada, 52.57847 -131.44319, 15.7.2023, L. Lee, D. Okamoto, L. Vigneault & A. Galloway |  |  |  | PQ888545 |  |  |
| *Boreolithothamnion phymatodeum* | GWS049783 | Subtidal (4.6 m), rhodolith, Hotspring I., British Columbia, Canada, 52.57847 -131.44319, 15.7.2023, L. Lee, D. Okamoto, L. Vigneault & A. Galloway |  |  |  | PQ888732 |  |  |
| *Boreolithothamnion phymatodeum* | GWS049784 | Subtidal (4.6 m), rhodolith, Hotspring I., British Columbia, Canada, 52.57847 -131.44319, 15.7.2023, L. Lee, D. Okamoto, L. Vigneault & A. Galloway |  |  |  | PQ888464 |  |  |
| *Boreolithothamnion phymatodeum* | GWS049785 | Subtidal (4.6 m), rhodolith, Hotspring I., British Columbia, Canada, 52.57847 -131.44319, 15.7.2023, L. Lee, D. Okamoto, L. Vigneault & A. Galloway |  |  |  | PQ888599 |  |  |
| *Boreolithothamnion phymatodeum* | GWS049788 | Subtidal (4.6 m), rhodolith, Hotspring I., British Columbia, Canada, 52.57847 -131.44319, 15.7.2023, L. Lee, D. Okamoto, L. Vigneault & A. Galloway |  |  |  | PQ888709 |  |  |
| *Boreolithothamnion phymatodeum* | GWS049792 | Subtidal (4.6 m), rhodolith, Hotspring I., British Columbia, Canada, 52.57847 -131.44319, 15.7.2023, L. Lee, D. Okamoto, L. Vigneault & A. Galloway |  |  |  | PQ888619 |  |  |
| *Boreolithothamnion phymatodeum* | GWS049794 | Subtidal (4.6 m), rhodolith, Hotspring I., British Columbia, Canada, 52.57847 -131.44319, 15.7.2023, L. Lee, D. Okamoto, L. Vigneault & A. Galloway |  |  |  | PQ888509 |  |  |
| *Boreolithothamnion phymatodeum* | GWS049795 | Subtidal (4.6 m), rhodolith, Hotspring I., British Columbia, Canada, 52.57847 -131.44319, 15.7.2023, L. Lee, D. Okamoto, L. Vigneault & A. Galloway |  |  |  | PQ888722 |  |  |
| *Boreolithothamnion phymatodeum* | GWS049797 | Subtidal (4.6 m), rhodolith, Hotspring I., British Columbia, Canada, 52.57847 -131.44319, 15.7.2023, L. Lee, D. Okamoto, L. Vigneault & A. Galloway |  |  |  | PQ888705 |  |  |
| *Boreolithothamnion phymatodeum* | GWS049811 | Subtidal (20 m) on cobble, crust, Tanu I., British Columbia, Canada, 52.76233 -131.61822, 23.7.2023, L. Lee, D. Okamoto, L. Vigneault & A. Galloway |  | PQ882846 |  | PQ888505 |  |  |
| *Boreolithothamnion phymatodeum* | GWS049812 | Subtidal (20 m) on shell, crust, Tanu I., British Columbia, Canada, 52.76233 -131.61822, 23.7.2023, L. Lee, D. Okamoto, L. Vigneault & A. Galloway |  |  |  | PQ888521 |  |  |
| *Boreolithothamnion phymatodeum* | GWS049873 | Subtidal (4 m), rhodolith, Stansung I., British Columbia, Canada, 52.73877 -131.61853, 23.7.2023, L. Lee, D. Okamoto, L. Vigneault & A. Galloway |  | PQ882806 |  | PQ888459 |  |  |
| *Boreolithothamnion phymatodeum* | GWS049874 | Subtidal (4 m), rhodolith, Stansung I., British Columbia, Canada, 52.73877 -131.61853, 23.7.2023, L. Lee, D. Okamoto, L. Vigneault & A. Galloway |  |  |  | PQ888640 |  |  |
| *Boreolithothamnion phymatodeum* | GWS049875 | Subtidal (4 m) on cobble, crust, Stansung I., British Columbia, Canada, 52.73877 -131.61853, 23.7.2023, L. Lee, D. Okamoto, L. Vigneault & A. Galloway |  |  |  | PQ888539 |  |  |
| *Boreolithothamnion phymatodeum* | GWS049876 | Subtidal (4 m) on cobble, crust, Stansung I., British Columbia, Canada, 52.73877 -131.61853, 23.7.2023, L. Lee, D. Okamoto, L. Vigneault & A. Galloway |  |  |  | PQ888587 |  |  |
| *Boreolithothamnion phymatodeum* | GWS049877 | Subtidal (4 m) on cobble, crust, Stansung I., British Columbia, Canada, 52.73877 -131.61853, 23.7.2023, L. Lee, D. Okamoto, L. Vigneault & A. Galloway |  | PQ882910 |  | PQ888582 |  |  |
| *Boreolithothamnion phymatodeum* | GWS049878 | Subtidal (4 m), rhodolith, Stansung I., British Columbia, Canada, 52.73877 -131.61853, 23.7.2023, L. Lee, D. Okamoto, L. Vigneault & A. Galloway |  |  |  | PQ888430 |  |  |
| *Boreolithothamnion phymatodeum* | GWS049879 | Subtidal (4 m), rhodolith, Stansung I., British Columbia, Canada, 52.73877 -131.61853, 23.7.2023, L. Lee, D. Okamoto, L. Vigneault & A. Galloway |  | PQ882901 |  | PQ888577 |  |  |
| *Boreolithothamnion phymatodeum* | GWS049881 | Subtidal (4 m) on cobble, crust, Stansung I., British Columbia, Canada, 52.73877 -131.61853, 23.7.2023, L. Lee, D. Okamoto, L. Vigneault & A. Galloway |  | PQ882818 |  | PQ888476 |  |  |
| *Boreolithothamnion phymatodeum* | GWS049882 | Subtidal (4 m) on cobble, crust, Stansung I., British Columbia, Canada, 52.73877 -131.61853, 23.7.2023, L. Lee, D. Okamoto, L. Vigneault & A. Galloway |  | PQ882895 |  | PQ888571 |  |  |
| *Boreolithothamnion phymatodeum* | GWS049883 | Subtidal (4 m), rhodolith, Stansung I., British Columbia, Canada, 52.73877 -131.61853, 23.7.2023, L. Lee, D. Okamoto, L. Vigneault & A. Galloway |  |  |  | PQ888678 |  |  |
| *Boreolithothamnion phymatodeum* | GWS049884 | Subtidal (4 m), rhodolith, Stansung I., British Columbia, Canada, 52.73877 -131.61853, 23.7.2023, L. Lee, D. Okamoto, L. Vigneault & A. Galloway |  |  |  | PQ888463 |  |  |
| *Boreolithothamnion phymatodeum* | GWS049885 | Subtidal (4 m) on cobble, crust, Stansung I., British Columbia, Canada, 52.73877 -131.61853, 23.7.2023, L. Lee, D. Okamoto, L. Vigneault & A. Galloway |  | PQ882907 |  | PQ888580 |  |  |
| *Boreolithothamnion phymatodeum* | GWS049886 | Subtidal (4 m) on cobble, crust, Stansung I., British Columbia, Canada, 52.73877 -131.61853, 23.7.2023, L. Lee, D. Okamoto, L. Vigneault & A. Galloway |  | PQ882892 |  | PQ888569 |  |  |
| *Boreolithothamnion phymatodeum* | GWS049887 | Subtidal (4 m), rhodolith, Stansung I., British Columbia, Canada, 52.73877 -131.61853, 23.7.2023, L. Lee, D. Okamoto, L. Vigneault & A. Galloway |  | PQ882996 |  | PQ888691 |  |  |
| *Boreolithothamnion phymatodeum* | GWS049888 | Subtidal (4 m), rhodolith, Stansung I., British Columbia, Canada, 52.73877 -131.61853, 23.7.2023, L. Lee, D. Okamoto, L. Vigneault & A. Galloway |  |  |  | PQ888681 |  |  |
| *Boreolithothamnion phymatodeum* | GWS049889 | Subtidal (4 m), rhodolith, Stansung I., British Columbia, Canada, 52.73877 -131.61853, 23.7.2023, L. Lee, D. Okamoto, L. Vigneault & A. Galloway |  | PQ882972 |  | PQ888660 |  |  |
| *Boreolithothamnion phymatodeum* | GWS049890 | Subtidal (4 m) on cobble, crust, Stansung I., British Columbia, Canada, 52.73877 -131.61853, 23.7.2023, L. Lee, D. Okamoto, L. Vigneault & A. Galloway |  |  |  | PQ888553 |  |  |
| *Boreolithothamnion phymatodeum* | GWS049892 | Subtidal (4 m), rhodolith, Stansung I., British Columbia, Canada, 52.73877 -131.61853, 23.7.2023, L. Lee, D. Okamoto, L. Vigneault & A. Galloway |  |  |  | PQ888684 |  |  |
| *Boreolithothamnion phymatodeum* | GWS049893 | Subtidal (4 m), rhodolith, Stansung I., British Columbia, Canada, 52.73877 -131.61853, 23.7.2023, L. Lee, D. Okamoto, L. Vigneault & A. Galloway |  | PQ882965 |  | PQ888648 |  |  |
| *Boreolithothamnion phymatodeum* | GWS049894 | Subtidal (4 m) on cobble, crust, Stansung I., British Columbia, Canada, 52.73877 -131.61853, 23.7.2023, L. Lee, D. Okamoto, L. Vigneault & A. Galloway |  |  |  | PQ888454 |  |  |
| *Boreolithothamnion phymatodeum* | GWS049895 | Subtidal (4 m), rhodolith, Stansung I., British Columbia, Canada, 52.73877 -131.61853, 23.7.2023, L. Lee, D. Okamoto, L. Vigneault & A. Galloway |  |  |  | PQ888507 |  |  |
| *Boreolithothamnion phymatodeum* | GWS049896 | Subtidal (4 m), rhodolith, Stansung I., British Columbia, Canada, 52.73877 -131.61853, 23.7.2023, L. Lee, D. Okamoto, L. Vigneault & A. Galloway |  | PQ882903 |  | PQ888579 |  |  |
| *Boreolithothamnion phymatodeum* | GWS049897 | Subtidal (4 m) on cobble, crust, Stansung I., British Columbia, Canada, 52.73877 -131.61853, 23.7.2023, L. Lee, D. Okamoto, L. Vigneault & A. Galloway |  |  |  | PQ888590 |  |  |
| *Boreolithothamnion soriferum* | GWS020720 | Subtidal (10 m) on worm tube, rhodolith, Kwuna I., Haida Gwaii, British Columbia, Canada, 53.21722 -131.98589, 6.6.2010, G.W. Saunders & K. Dixon | HQ545039 |  |  | PQ888490 |  |  |
| *Boreolithothamnion soriferum* | GWS028057 | Subtidal (8 m), rhodolith, Channel btw Murchison & Faraday I., Gwaii Haanas, British Columbia, Canada, 52.59687 -131.47513, 7.7.2011, G.W. Saunders & K. Dixon | MW536938 |  | PQ882982 | PQ888673 |  |  |
| *Boreolithothamnion soriferum* | GWS048576 | Subtidal (5 m), rhodolith, Channel btw Murchison & Faraday I., Gwaii Haanas, British Columbia, Canada, 52.59687 -131.47513, 2.8.2022, G.W. Saunders & C. Brooks |  | PQ882934 |  | PQ888604 | PQ883078 |  |
| *Boreolithothamnion soriferum* | GWS049710 | Subtidal (6.5 m), rhodolith, Hotspring I., British Columbia, Canada, 52.57847 -131.44319, 15.7.2023, L. Lee, D. Okamoto, L. Vigneault & A. Galloway |  |  |  | PQ888610 |  |  |
| *Boreolithothamnion soriferum* | GWS049711 | Subtidal (6.5 m), rhodolith, Hotspring I., British Columbia, Canada, 52.57847 -131.44319, 15.7.2023, L. Lee, D. Okamoto, L. Vigneault & A. Galloway |  |  |  | PQ888447 |  |  |
| *Boreolithothamnion soriferum* | GWS049723 | Subtidal (6.5 m), rhodolith, Hotspring I., British Columbia, Canada, 52.57847 -131.44319, 15.7.2023, L. Lee, D. Okamoto, L. Vigneault & A. Galloway |  |  |  | PQ888485 |  |  |
| *Boreolithothamnion soriferum* | GWS049753 | Subtidal (4.6 m), rhodolith, Hotspring I., British Columbia, Canada, 52.57847 -131.44319, 15.7.2023, L. Lee, D. Okamoto, L. Vigneault & A. Galloway |  |  |  | PQ888687 |  |  |
| *Boreolithothamnion soriferum* | GWS049755 | Subtidal (4.6 m), rhodolith, Hotspring I., British Columbia, Canada, 52.57847 -131.44319, 15.7.2023, L. Lee, D. Okamoto, L. Vigneault & A. Galloway |  | PQ882805 |  | PQ888457 |  |  |
| *Boreolithothamnion soriferum* | GWS049791 | Subtidal (4.6 m), rhodolith, Hotspring I., British Columbia, Canada, 52.57847 -131.44319, 15.7.2023, L. Lee, D. Okamoto, L. Vigneault & A. Galloway |  |  |  | PQ888676 |  |  |
| *Boreolithothamnion soriferum* | GWS049819 | Subtidal (10.4 m), rhodolith, Tanu I., British Columbia, Canada, 52.76233 -131.61822, 23.7.2023, L. Lee, D. Okamoto, L. Vigneault & A. Galloway |  |  |  | PQ888434 |  |  |
| *Boreolithothamnion soriferum* | GWS049820 | Subtidal (10.4 m), rhodolith, Tanu I., British Columbia, Canada, 52.76233 -131.61822, 23.7.2023, L. Lee, D. Okamoto, L. Vigneault & A. Galloway |  |  |  | PQ888700 |  |  |
| *Boreolithothamnion soriferum* | GWS049859 | Subtidal (6.5 m), rhodolith, Stansung I., British Columbia, Canada, 52.73877 -131.61853, 23.7.2023, L. Lee, D. Okamoto, L. Vigneault & A. Galloway |  | PQ882944 |  | PQ888623 |  |  |
| *Boreolithothamnion* sp. 1cocos | GWS037289 | Subtidal (5 m), on cabbage coral rubble, crust, Blue Holes (inner lagoon east), Cocos (Keeling), Australia, -12.15365 96.08007, 9.12.2013, G.W. Saunders & K. Dixon | PQ883043 |  | PQ882921 |  |  |  |
| *Boreolithothamnion* sp. 1glaciale | GWS014320 | Subtidal (8 m) on shell, crust, Bamfield, Scotts Bay, British Columbia, Canada, 48.8346 -125.1463, 25.6.2011, G.W. Saunders & K. Dixon | MW536950 | PQ882986 |  | PQ888679 | PQ883087 | PQ883128 |
| *Boreolithothamnion* sp. 1glaciale | GWS019375 | Subtidal (10 m) on inverts, crust, Bamfield, Seapool Rock, British Columbia, Canada, 48.8155 -125.2086, 31.5.2010, G.W. Saunders & K. Dixon | HQ544362 |  | PQ882828 | PQ888489 |  |  |
| *Boreolithothamnion* sp. 1glaciale | GWS046263 | Subtidal (1.5 m) on limpet, crust, Murchison I. NE site, Plot 1 (2-5 ft), Gwaii Haanas, British Columbia, Canada, 52.61563 -131.43755, 16.7.2019, G.W. Saunders & C. Brooks |  |  |  |  |  | PQ883104 |
| *Boreolithothamnion* sp. 1heterocladum | GWS009195 | Subtidal (11 m) on rock, crust, Bamfield, Wizard I., British Columbia, Canada, 48.8583 -125.1588, 23.9.2007, G.W. Saunders & B. Clarkston |  | PQ882844 |  |  |  |  |
| *Boreolithothamnion* sp. 1heterocladum | GWS012665 | Subtidal (10 m) on rock, crust, Mazarredo I.s, NW of Masset, Haida Gwaii, British Columbia, Canada, 54.09051 -132.55092, 17.6.2009, G.W. Saunders & D. McDevit | HM918903 |  |  |  |  |  |
| *Boreolithothamnion* sp. 1heterocladum | GWS014308 | Subtidal (10 m) on shell, crust, Bamfield, Scotts Bay, British Columbia, Canada, 48.8346 -125.1463, 25.6.2011, G.W. Saunders & K. Dixon | HM916165 |  |  |  |  | PQ883103 |
| *Boreolithothamnion* sp. 1heterocladum | GWS019912 | Low intertidal on snail, semi-exposed, crust, Koga Islet, Gwaii Haanas, British Columbia, Canada, 52.42831 -131.3786, 12.6.2010, G.W. Saunders & K. Dixon | MW536978 |  |  |  |  |  |
| *Boreolithothamnion* sp. 1heterocladum | GWS020771 | Subtidal (10 m) on invert, crust, Kwuna I., Haida Gwaii, British Columbia, Canada, 53.21722 -131.98589, 6.6.2010, G.W. Saunders & K. Dixon | MW536974 |  |  |  |  | PQ883093 |
| *Boreolithothamnion* sp. 1heterocladum | GWS020784 | Subtidal (10 m) on bottle, crust, Kwuna I., Haida Gwaii, British Columbia, Canada, 53.21722 -131.98589, 6.6.2010, G.W. Saunders & K. Dixon | MW536979 |  |  |  |  |  |
| *Boreolithothamnion* sp. 1heterocladum | GWS028036 | Subtidal (5 m) on snail, crust, Lost I.s, NE Bay, Gwaii Haanas, British Columbia, Canada, 52.80365 -131.4838, 6.7.2011, G.W. Saunders & K. Dixon | MW536949 | PQ882932 |  |  |  | PQ883121 |
| *Boreolithothamnion* sp. 1heterocladum | GWS045433 | Subtidal (8 m) on cobble, crust, Faraday I., N Site, Plot 1 (10-30 ft), Gwaii Haanas, British Columbia, Canada, 52.61523 -131.46466, 18.7.2019, G.W. Saunders & C. Brooks | MW536980 |  |  |  |  | PQ883126 |
| *Boreolithothamnion* sp. 1heterocladum | GWS045449 | Subtidal (2.5 m) on limpet, crust, Faraday I., N Site, Plot 1 (5-10 ft), Gwaii Haanas, British Columbia, Canada, 52.61523 -131.46466, 18.7.2019, G.W. Saunders & C. Brooks |  |  | PQ882859 |  |  |  |
| *Boreolithothamnion* sp. 1heterocladum | GWS045632 | Subtidal (6 m) on shell, crust, Faraday I., S Site, Plot 2 (10-30 ft), Gwaii Haanas, British Columbia, Canada, 52.60913 -131.46582, 19.7.2019, G.W. Saunders & C. Brooks | MW536955 |  |  |  |  | PQ883133 |
| *Boreolithothamnion* sp. 1heterocladum | GWS046278 | Subtidal (10 m) on shell, crust, Murchison I. NW site, Plot 1 (10-30 ft), Gwaii Haanas, British Columbia, Canada, 52.61456 -131.44443, 16.7.2019, G.W. Saunders & C. Brooks |  |  |  |  |  |  |
| *Boreolithothamnion* sp. 1heterocladum | GWS046323 | Subtidal (1.5 m) on limpet, crust, Murchison I. NW site, Plot 1 (2-5 ft), Gwaii Haanas, British Columbia, Canada, 52.61456 -131.44443, 16.7.2019, G.W. Saunders & C. Brooks | PQ883045 |  |  |  |  | PQ883122 |
| *Boreolithothamnion* sp. 1heterocladum | GWS046324 | Subtidal (1.5 m) on limpet, crust, Murchison I. NW site, Plot 1 (2-5 ft), Gwaii Haanas, British Columbia, Canada, 52.61456 -131.44443, 16.7.2019, G.W. Saunders & C. Brooks |  | PQ882966 |  |  |  |  |
| *Boreolithothamnion* sp. 1heterocladum | GWS046378 | Subtidal (5 m) on shell, crust, Murchison I. NW site, Plot 1 (10-30 ft), Gwaii Haanas, British Columbia, Canada, 52.61456 -131.44443, 17.7.2019, G.W. Saunders & C. Brooks | PQ883023 |  |  | PQ888456 |  | PQ883094 |
| *Boreolithothamnion* sp. 1heterocladum | GWS046701 | Subtidal (7 m), rhodolith, Channel btw Murchison & Faraday I., Gwaii Haanas, British Columbia, Canada, 52.59703 -131.47345, 27.7.2022, G.W. Saunders & C. Brooks |  | PQ882960 |  | PQ888643 | PQ883083 | PQ883124 |
| *Boreolithothamnion* sp. 1heterocladum | GWS046764 | Subtidal (10 m) on rock, crust, Faraday I., S Site, Plot 2 (10-30 ft), Gwaii Haanas, British Columbia, Canada, 52.60913 -131.46582, 27.7.2022, G.W. Saunders & C. Brooks |  | PQ882845 |  |  |  |  |
| *Boreolithothamnion* sp. 1heterocladum | GWS046769 | Subtidal (10 m) on shell, crust, Faraday I., S Site, Plot 2 (10-30 ft), Gwaii Haanas, British Columbia, Canada, 52.60913 -131.46582, 27.7.2022, G.W. Saunders & C. Brooks |  | PQ882889 |  |  |  |  |
| *Boreolithothamnion* sp. 1heterocladum | GWS046835 | Subtidal (10 m) on limpet, crust, Murchison I. NW site, Plot 2 (10-30 ft) Gwaii Haanas, British Columbia, Canada, 52.60913 -131.46582, 28.7.2022, G.W. Saunders & C. Brooks |  | PQ882862 |  |  |  |  |
| *Boreolithothamnion* sp. 1heterocladum | GWS046975 | Subtidal (10 m) on rock, crust, Arnold Pt. (east side, left transects), Gwaii Haanas, British Columbia, Canada, 52.09895 -131.13159, 30.7.2022, G.W. Saunders & C. Brooks |  | PQ882937 |  | PQ888611 |  |  |
| *Boreolithothamnion* sp. 1heterocladum | GWS047633 | Subtidal (10 m) on rock, crust, Bischof I.s (north shore), Gwaii Haanas, British Columbia, Canada, 52.58118 -131.562, 4.9.2021, G.W. Saunders & C. Brooks |  | PQ882891 |  |  |  |  |
| *Boreolithothamnion* sp. 1heterocladum | GWS047647 | Subtidal (5 m) on limpet, crust, Bischof I.s (north shore), Gwaii Haanas, British Columbia, Canada, 52.58118 -131.562, 4.9.2021, G.W. Saunders & C. Brooks |  | PQ883017 |  |  |  |  |
| *Boreolithothamnion* sp. 1heterocladum | GWS047774 | Subtidal (10 m) on limpet, crust, Murchison I. NW site, Plot 2 (10-30 ft) Gwaii Haanas, British Columbia, Canada, 52.61456 -131.44443, 5.9.2021, G.W. Saunders & C. Brooks | PQ883021 | PQ882783 |  |  |  |  |
| *Boreolithothamnion* sp. 1heterocladum | GWS047925 | Subtidal (10 m) on turbin snail, crust, Murchison I. NW, east of Plot 1, Gwaii Haanas, British Columbia, Canada, 52.61517 -131.44183, 6.9.2021, G.W. Saunders & C. Brooks |  | PQ882997 |  |  |  |  |
| *Boreolithothamnion* sp. 1heterocladum | GWS048007 | Subtidal (7.5 m) on cobble, crust, Faraday I., S Site, Plot 2 (10-30 ft), Gwaii Haanas, British Columbia, Canada, 52.60913 -131.46582, 8.9.2021, G.W. Saunders & C. Brooks |  |  |  |  |  |  |
| *Boreolithothamnion* sp. 1heterocladum | GWS048009 | Subtidal (7.5 m) on cobble, crust, Faraday I., S Site, Plot 2 (10-30 ft), Gwaii Haanas, British Columbia, Canada, 52.60913 -131.46582, 8.9.2021, G.W. Saunders & C. Brooks |  |  |  |  |  |  |
| *Boreolithothamnion* sp. 1heterocladum | GWS048111 | Subtidal (7.5 m) on limpet shell, crust, Murchison I. NW site, Plot 1 (10-30 ft), Gwaii Haanas, British Columbia, Canada, 52.61456 -131.44443, 8.9.2021, G.W. Saunders & C. Brooks |  | PQ882931 |  |  |  |  |
| *Boreolithothamnion* sp. 1heterocladum | GWS048274 | Subtidal (6 m) on limpet shell, crust, Gordon I.s (East; 6 m), Gwaii Haanas, British Columbia, Canada, 52.099867 -131.146133, 12.9.2021, G.W. Saunders & C. Brooks |  | PQ882824 |  | PQ888480 |  | PQ883099 |
| *Boreolithothamnion* sp. 1heterocladum | GWS048401 | Subtidal (5 m) on shell, crust, Arnold Pt. (east side, left transects), Gwaii Haanas, British Columbia, Canada, 52.09895 -131.13159, 30.7.2022, G.W. Saunders & C. Brooks |  | PQ882940 |  |  |  |  |
| *Boreolithothamnion* sp. 1heterocladum | GWS048421 | Subtidal (2 m) on limpet, crust, Arnold Pt. (east side, left transects), Gwaii Haanas, British Columbia, Canada, 52.09895 -131.13159, 30.7.2022, G.W. Saunders & C. Brooks |  | PQ882962 |  | PQ888645 |  |  |
| *Boreolithothamnion* sp. 1heterocladum | GWS048557 | Subtidal (10 m) on rock, crust, Murchison I. NE site, Plot 1 (2-5 ft), Gwaii Haanas, British Columbia, Canada, 52.61563 -131.43754, 1.8.2022, G.W. Saunders & C. Brooks |  |  |  | PQ888568 |  |  |
| *Boreolithothamnion* sp. 1heterocladum | GWS048635 | Subtidal (10 m) on limpet, crust, Fuller Pt. (Windy Bay), Gwaii Haanas, British Columbia, Canada, 52.69953 -131.44072, 3.8.2022, G.W. Saunders & C. Brooks |  |  |  | PQ888614 |  |  |
| *Boreolithothamnion* sp. 1heterocladum | GWS049705 (Holotype) | Subtidal (6.5 m), rhodolith, Hotspring I., British Columbia, Canada, 52.57847 -131.44319, 15.7.2023, L. Lee, D. Okamoto, L. Vigneault & A. Galloway |  | PQ882992 |  | PQ888686 |  | PQ883129 |
| *Boreolithothamnion* sp. 1heterocladum | GWS049706 (Isotype) | Subtidal (6.5 m), rhodolith, Hotspring I., British Columbia, Canada, 52.57847 -131.44319, 15.7.2023, L. Lee, D. Okamoto, L. Vigneault & A. Galloway |  | PQ882861 |  |  |  | PQ883106 |
| *Boreolithothamnion* sp. 1heterocladum | GWS049715 (Isotype) | Subtidal (6.5 m), rhodolith, Hotspring I., British Columbia, Canada, 52.57847 -131.44319, 15.7.2023, L. Lee, D. Okamoto, L. Vigneault & A. Galloway | PQ883046 |  |  | PQ888649 |  |  |
| *Boreolithothamnion* sp. 1heterocladum | GWS049716 (Isotype) | Subtidal (6.5 m), rhodolith, Hotspring I., British Columbia, Canada, 52.57847 -131.44319, 15.7.2023, L. Lee, D. Okamoto, L. Vigneault & A. Galloway |  |  |  | PQ888594 |  |  |
| *Boreolithothamnion* sp. 1heterocladum | GWS049719 (Isotype) | Subtidal (6.5 m), rhodolith, Hotspring I., British Columbia, Canada, 52.57847 -131.44319, 15.7.2023, L. Lee, D. Okamoto, L. Vigneault & A. Galloway |  |  |  | PQ888651 |  |  |
| *Boreolithothamnion* sp. 1heterocladum | GWS049721 (Isotype) | Subtidal (6.5 m) on shell, crust, Hotspring I., British Columbia, Canada, 52.57847 -131.44319, 15.7.2023, L. Lee, D. Okamoto, L. Vigneault & A. Galloway |  | PQ882795 |  | PQ888443 |  |  |
| *Boreolithothamnion* sp. 1heterocladum | GWS049722 (Isotype) | Subtidal (6.5 m), rhodolith, Hotspring I., British Columbia, Canada, 52.57847 -131.44319, 15.7.2023, L. Lee, D. Okamoto, L. Vigneault & A. Galloway |  |  |  | PQ888664 |  |  |
| *Boreolithothamnion* sp. 1heterocladum | GWS049727 (Isotype) | Subtidal (6.5 m), rhodolith, Hotspring I., British Columbia, Canada, 52.57847 -131.44319, 15.7.2023, L. Lee, D. Okamoto, L. Vigneault & A. Galloway |  |  |  | PQ888517 |  |  |
| *Boreolithothamnion* sp. 1heterocladum | GWS049728 (Isotype) | Subtidal (6.5 m) on cobble, crust, Hotspring I., British Columbia, Canada, 52.57847 -131.44319, 15.7.2023, L. Lee, D. Okamoto, L. Vigneault & A. Galloway |  | PQ882969 |  | PQ888653 |  |  |
| *Boreolithothamnion* sp. 1heterocladum | GWS049730 (Isotype) | Subtidal (6.5 m) on cobble, crust, Hotspring I., British Columbia, Canada, 52.57847 -131.44319, 15.7.2023, L. Lee, D. Okamoto, L. Vigneault & A. Galloway |  |  |  | PQ888437 |  |  |
| *Boreolithothamnion* sp. 1heterocladum | GWS049731 (Isotype) | Subtidal (6.5 m), rhodolith, Hotspring I., British Columbia, Canada, 52.57847 -131.44319, 15.7.2023, L. Lee, D. Okamoto, L. Vigneault & A. Galloway |  |  |  | PQ888717 |  |  |
| *Boreolithothamnion* sp. 1heterocladum | GWS049737 (Isotype) | Subtidal (6.5 m) on cobble, crust, Hotspring I., British Columbia, Canada, 52.57847 -131.44319, 15.7.2023, L. Lee, D. Okamoto, L. Vigneault & A. Galloway |  |  |  | PQ888547 |  |  |
| *Boreolithothamnion* sp. 1heterocladum | GWS049738 (Isotype) | Subtidal (6.5 m) on cobble, crust, Hotspring I., British Columbia, Canada, 52.57847 -131.44319, 15.7.2023, L. Lee, D. Okamoto, L. Vigneault & A. Galloway |  | PQ882833 |  | PQ888495 |  |  |
| *Boreolithothamnion* sp. 1heterocladum | GWS049740 (Isotype) | Subtidal (6.5 m) on cobble, crust, Hotspring I., British Columbia, Canada, 52.57847 -131.44319, 15.7.2023, L. Lee, D. Okamoto, L. Vigneault & A. Galloway | PQ883029 |  |  | PQ888511 |  |  |
| *Boreolithothamnion* sp. 1heterocladum | GWS049743 (Isotype) | Subtidal (6.5 m), rhodolith, Hotspring I., British Columbia, Canada, 52.57847 -131.44319, 15.7.2023, L. Lee, D. Okamoto, L. Vigneault & A. Galloway |  |  |  | PQ888638 |  |  |
| *Boreolithothamnion* sp. 1heterocladum | GWS049745 (Isotype) | Subtidal (6.5 m) on cobble, crust, Hotspring I., British Columbia, Canada, 52.57847 -131.44319, 15.7.2023, L. Lee, D. Okamoto, L. Vigneault & A. Galloway |  | PQ882936 |  | PQ888607 |  |  |
| *Boreolithothamnion* sp. 1heterocladum | GWS049747 (Isotype) | Subtidal (6.5 m) on cobble, crust, Hotspring I., British Columbia, Canada, 52.57847 -131.44319, 15.7.2023, L. Lee, D. Okamoto, L. Vigneault & A. Galloway |  | PQ882796 |  | PQ888445 |  |  |
| *Boreolithothamnion* sp. 1heterocladum | GWS049750 (Isotype) | Subtidal (6.5 m) on cobble, crust, Hotspring I., British Columbia, Canada, 52.57847 -131.44319, 15.7.2023, L. Lee, D. Okamoto, L. Vigneault & A. Galloway |  |  |  | PQ888462 |  |  |
| *Boreolithothamnion* sp. 1heterocladum | GWS049751 (Isotype) | Subtidal (4.6 m), rhodolith, Hotspring I., British Columbia, Canada, 52.57847 -131.44319, 15.7.2023, L. Lee, D. Okamoto, L. Vigneault & A. Galloway |  |  |  | PQ888628 |  |  |
| *Boreolithothamnion* sp. 1heterocladum | GWS049754 (Isotype) | Subtidal (4.6 m), rhodolith, Hotspring I., British Columbia, Canada, 52.57847 -131.44319, 15.7.2023, L. Lee, D. Okamoto, L. Vigneault & A. Galloway |  | PQ882827 |  | PQ888488 |  |  |
| *Boreolithothamnion* sp. 1heterocladum | GWS049757 (Isotype) | Subtidal (4.6 m), rhodolith, Hotspring I., British Columbia, Canada, 52.57847 -131.44319, 15.7.2023, L. Lee, D. Okamoto, L. Vigneault & A. Galloway |  |  |  | PQ888525 |  |  |
| *Boreolithothamnion* sp. 1heterocladum | GWS049766 (Isotype) | Subtidal (4.6 m), rhodolith, Hotspring I., British Columbia, Canada, 52.57847 -131.44319, 15.7.2023, L. Lee, D. Okamoto, L. Vigneault & A. Galloway |  | PQ883006 |  | PQ888712 |  |  |
| *Boreolithothamnion* sp. 1heterocladum | GWS049767 (Isotype) | Subtidal (4.6 m), rhodolith, Hotspring I., British Columbia, Canada, 52.57847 -131.44319, 15.7.2023, L. Lee, D. Okamoto, L. Vigneault & A. Galloway |  | PQ882893 |  | PQ888570 |  |  |
| *Boreolithothamnion* sp. 1heterocladum | GWS049773 (Isotype) | Subtidal (4.6 m), rhodolith, Hotspring I., British Columbia, Canada, 52.57847 -131.44319, 15.7.2023, L. Lee, D. Okamoto, L. Vigneault & A. Galloway |  | PQ882995 |  | PQ888690 |  |  |
| *Boreolithothamnion* sp. 1heterocladum | GWS049774 (Isotype) | Subtidal (4.6 m), rhodolith, Hotspring I., British Columbia, Canada, 52.57847 -131.44319, 15.7.2023, L. Lee, D. Okamoto, L. Vigneault & A. Galloway |  | PQ882807 |  | PQ888466 |  |  |
| *Boreolithothamnion* sp. 1heterocladum | GWS049777 (Isotype) | Subtidal (4.6 m), rhodolith, Hotspring I., British Columbia, Canada, 52.57847 -131.44319, 15.7.2023, L. Lee, D. Okamoto, L. Vigneault & A. Galloway |  | PQ882983 |  | PQ888675 |  |  |
| *Boreolithothamnion* sp. 1heterocladum | GWS049779 (Isotype) | Subtidal (4.6 m), rhodolith, Hotspring I., British Columbia, Canada, 52.57847 -131.44319, 15.7.2023, L. Lee, D. Okamoto, L. Vigneault & A. Galloway |  |  |  | PQ888552 |  |  |
| *Boreolithothamnion* sp. 1heterocladum | GWS049780 (Isotype) | Subtidal (4.6 m), rhodolith, Hotspring I., British Columbia, Canada, 52.57847 -131.44319, 15.7.2023, L. Lee, D. Okamoto, L. Vigneault & A. Galloway |  | PQ883010 |  | PQ888721 |  |  |
| *Boreolithothamnion* sp. 1heterocladum | GWS049786 (Isotype) | Subtidal (4.6 m), rhodolith, Hotspring I., British Columbia, Canada, 52.57847 -131.44319, 15.7.2023, L. Lee, D. Okamoto, L. Vigneault & A. Galloway |  | PQ882971 |  | PQ888658 |  |  |
| *Boreolithothamnion* sp. 1heterocladum | GWS049787 (Isotype) | Subtidal (4.6 m), rhodolith, Hotspring I., British Columbia, Canada, 52.57847 -131.44319, 15.7.2023, L. Lee, D. Okamoto, L. Vigneault & A. Galloway |  | PQ882865 |  | PQ888536 |  |  |
| *Boreolithothamnion* sp. 1heterocladum | GWS049789 (Isotype) | Subtidal (4.6 m), rhodolith, Hotspring I., British Columbia, Canada, 52.57847 -131.44319, 15.7.2023, L. Lee, D. Okamoto, L. Vigneault & A. Galloway |  | PQ882856 |  | PQ888519 |  |  |
| *Boreolithothamnion* sp. 1heterocladum | GWS049790 | Subtidal (4.6 m), rhodolith, Hotspring I., British Columbia, Canada, 52.57847 -131.44319, 15.7.2023, L. Lee, D. Okamoto, L. Vigneault & A. Galloway |  | PQ882950 |  | PQ888627 |  | PQ883123 |
| *Boreolithothamnion* sp. 1heterocladum | GWS049796 | Subtidal (4.6 m) on cobble, crust, Hotspring I., British Columbia, Canada, 52.57847 -131.44319, 15.7.2023, L. Lee, D. Okamoto, L. Vigneault & A. Galloway |  |  |  | PQ888482 |  |  |
| *Boreolithothamnion* sp. 1heterocladum | GWS049798 | Subtidal (4.6 m), rhodolith, Hotspring I., British Columbia, Canada, 52.57847 -131.44319, 15.7.2023, L. Lee, D. Okamoto, L. Vigneault & A. Galloway |  | PQ882990 |  | PQ888685 |  |  |
| *Boreolithothamnion* sp. 1heterocladum | GWS049799 | Subtidal (4.6 m), rhodolith, Hotspring I., British Columbia, Canada, 52.57847 -131.44319, 15.7.2023, L. Lee, D. Okamoto, L. Vigneault & A. Galloway |  | PQ882830 |  | PQ888492 |  |  |
| *Boreolithothamnion* sp. 1heterocladum | GWS049804 | Subtidal (11 m) on cobble, crust, Murchison NW (restoration site near Plot 1), British Columbia, Canada, 22.7.2023, L. Lee, D. Okamoto, L. Vigneault & A. Galloway |  |  |  | PQ888600 |  |  |
| *Boreolithothamnion* sp. 1heterocladum | GWS049808 | Subtidal (11 m) on shell, crust, Murchison NW (restoration site near Plot 1), British Columbia, Canada, 22.7.2023, L. Lee, D. Okamoto, L. Vigneault & A. Galloway |  | PQ882812 |  | PQ888472 |  |  |
| *Boreolithothamnion* sp. 1heterocladum | GWS049809 | Subtidal (11 m) on shell, crust, Murchison NW (restoration site near Plot 1), British Columbia, Canada, 22.7.2023, L. Lee, D. Okamoto, L. Vigneault & A. Galloway |  | PQ882935 |  | PQ888605 |  |  |
| *Boreolithothamnion* sp. 1heterocladum | GWS049810 | Subtidal (11 m) on shell, crust, Murchison NW (restoration site near Plot 1), British Columbia, Canada, 22.7.2023, L. Lee, D. Okamoto, L. Vigneault & A. Galloway |  | PQ882866 |  | PQ888537 |  |  |
| *Boreolithothamnion* sp. 1heterocladum | GWS049813 | Subtidal (10.4 m), rhodolith, Tanu I., British Columbia, Canada, 52.76233 -131.61822, 23.7.2023, L. Lee, D. Okamoto, L. Vigneault & A. Galloway |  |  |  | PQ888444 |  |  |
| *Boreolithothamnion* sp. 1heterocladum | GWS049856 | Subtidal (6.5 m), rhodolith, Stansung I., British Columbia, Canada, 52.73877 -131.61853, 23.7.2023, L. Lee, D. Okamoto, L. Vigneault & A. Galloway |  | PQ882791 |  | PQ888439 |  | PQ883092 |
| *Boreolithothamnion* sp. 1heterocladum | GWS049857 | Subtidal (6.5 m), rhodolith, Stansung I., British Columbia, Canada, 52.73877 -131.61853, 23.7.2023, L. Lee, D. Okamoto, L. Vigneault & A. Galloway |  | PQ882792 |  | PQ888440 |  |  |
| *Boreolithothamnion* sp. 1heterocladum | GWS049861 | Subtidal (6.5 m) on cobble, crust, Stansung I., British Columbia, Canada, 52.73877 -131.61853, 23.7.2023, L. Lee, D. Okamoto, L. Vigneault & A. Galloway |  | PQ882831 |  | PQ888494 |  |  |
| *Boreolithothamnion* sp. 1heterocladum | GWS049862 | Subtidal (6.5 m) on cobble, crust, Stansung I., British Columbia, Canada, 52.73877 -131.61853, 23.7.2023, L. Lee, D. Okamoto, L. Vigneault & A. Galloway |  | PQ882877 |  | PQ888550 |  |  |
| *Boreolithothamnion* sp. 1heterocladum | GWS049863 | Subtidal (6.5 m), rhodolith, Stansung I., British Columbia, Canada, 52.73877 -131.61853, 23.7.2023, L. Lee, D. Okamoto, L. Vigneault & A. Galloway |  |  |  | PQ888493 |  |  |
| *Boreolithothamnion* sp. 1heterocladum | GWS049864 | Subtidal (6.5 m), rhodolith, Stansung I., British Columbia, Canada, 52.73877 -131.61853, 23.7.2023, L. Lee, D. Okamoto, L. Vigneault & A. Galloway |  | PQ882878 |  | PQ888555 |  |  |
| *Boreolithothamnion* sp. 1heterocladum | GWS049865 | Subtidal (6.5 m) on cobble, crust, Stansung I., British Columbia, Canada, 52.73877 -131.61853, 23.7.2023, L. Lee, D. Okamoto, L. Vigneault & A. Galloway |  | PQ882999 |  | PQ888694 |  |  |
| *Boreolithothamnion* sp. 1heterocladum | GWS049866 | Subtidal (6.5 m), rhodolith, Stansung I., British Columbia, Canada, 52.73877 -131.61853, 23.7.2023, L. Lee, D. Okamoto, L. Vigneault & A. Galloway |  | PQ882784 |  | PQ888433 |  |  |
| *Boreolithothamnion* sp. 1heterocladum | GWS049867 | Subtidal (6.5 m) on cobble, crust, Stansung I., British Columbia, Canada, 52.73877 -131.61853, 23.7.2023, L. Lee, D. Okamoto, L. Vigneault & A. Galloway |  |  |  | PQ888677 |  |  |
| *Boreolithothamnion* sp. 1heterocladum | GWS049869 | Subtidal (6.5 m) on cobble, crust, Stansung I., British Columbia, Canada, 52.73877 -131.61853, 23.7.2023, L. Lee, D. Okamoto, L. Vigneault & A. Galloway |  | PQ883018 |  | PQ888731 |  |  |
| *Boreolithothamnion* sp. 1heterocladum | GWS049870 | Subtidal (6.5 m) on cobble, crust, Stansung I., British Columbia, Canada, 52.73877 -131.61853, 23.7.2023, L. Lee, D. Okamoto, L. Vigneault & A. Galloway |  | PQ882872 |  | PQ888544 |  |  |
| *Boreolithothamnion* sp. 1heterocladum | GWS049871 | Subtidal (6.5 m) on cobble, crust, Stansung I., British Columbia, Canada, 52.73877 -131.61853, 23.7.2023, L. Lee, D. Okamoto, L. Vigneault & A. Galloway |  | PQ882922 |  | PQ888592 |  |  |
| *Boreolithothamnion* sp. 1heterocladum | GWS049872 | Subtidal (6.5 m) on shell, crust, Stansung I., British Columbia, Canada, 52.73877 -131.61853, 23.7.2023, L. Lee, D. Okamoto, L. Vigneault & A. Galloway |  |  |  | PQ888479 |  |  |
| *Boreolithothamnion* sp. 1heterocladum | GWS049880 | Subtidal (4 m), rhodolith, Stansung I., British Columbia, Canada, 52.73877 -131.61853, 23.7.2023, L. Lee, D. Okamoto, L. Vigneault & A. Galloway |  | PQ882838 |  | PQ888499 |  |  |
| *Boreolithothamnion* sp. 1heterocladum | GWS049898 | Subtidal (4 m) on shell, crust, Stansung I., British Columbia, Canada, 52.73877 -131.61853, 23.7.2023, L. Lee, D. Okamoto, L. Vigneault & A. Galloway |  | PQ882880 |  | PQ888556 |  | PQ883113 |
| *Boreolithothamnion* sp. 1heterocladum | GWS049899 | Subtidal (4 m) on shell, crust, Stansung I., British Columbia, Canada, 52.73877 -131.61853, 23.7.2023, L. Lee, D. Okamoto, L. Vigneault & A. Galloway |  | PQ882882 |  | PQ888557 |  |  |
| *Boreolithothamnion* sp. 2Rhodolith | GWS018293 | Subtidal (10 m), rhodolith, Rocky Reef at Lighthouse 'Point' Piyangdo I., Jeju, South Korea, 33.4198 126.2244, 20.5.2010, G.W. Saunders & H-G. Choi | PQ883038 |  | PQ882879 |  | PQ883070 |  |
| *Boreolithothamnion tanuense* | GWS020939 | Subtidal (10 m) on rock, crust, Indian Head, Skidegate, Haida Gwaii, British Columbia, Canada, 53.24805 -131.98369, 7.6.2010, G.W. Saunders & K. Dixon | KM254875 |  | PQ882949 |  |  |  |
| *Boreolithothamnion tanuense* | GWS022118 | Subtidal (5 m) on rock, crust, Stillwater Cove, Pebble Beach, California, United States of America, 36.566674 -121.94289, 20.5.2010, B. Clarkston, K. Hind & S. Toews | KM254696 |  |  | MW536710 |  |  |
| *Boreolithothamnion tanuense* | GWS046279 | Subtidal (10 m) on turbin snail, crust, Murchison I. NW site, Plot 1 (10-30 ft), Gwaii Haanas, British Columbia, Canada, 52.61456 -131.44443, 16.7.2019, G.W. Saunders & C. Brooks |  | PQ882991 |  |  |  |  |
| *Boreolithothamnion tanuense* | GWS049704 | Subtidal (6.5 m), rhodolith, Hotspring I., British Columbia, Canada, 52.57847 -131.44319, 15.7.2023, L. Lee, D. Okamoto, L. Vigneault & A. Galloway |  | PQ882909 |  |  |  |  |
| *Boreolithothamnion tanuense* | GWS049735 | Subtidal (6.5 m) on cobble, crust, Hotspring I., British Columbia, Canada, 52.57847 -131.44319, 15.7.2023, L. Lee, D. Okamoto, L. Vigneault & A. Galloway |  |  |  | PQ888514 |  |  |
| *Boreolithothamnion tanuense* | GWS049748 | Subtidal (6.5 m), rhodolith, Hotspring I., British Columbia, Canada, 52.57847 -131.44319, 15.7.2023, L. Lee, D. Okamoto, L. Vigneault & A. Galloway |  |  |  | PQ888467 |  |  |
| *Boreolithothamnion tanuense* | GWS049775 | Subtidal (4.6 m), rhodolith, Hotspring I., British Columbia, Canada, 52.57847 -131.44319, 15.7.2023, L. Lee, D. Okamoto, L. Vigneault & A. Galloway |  |  |  | PQ888589 |  |  |
| *Boreolithothamnion tanuense* | GWS049805 | Subtidal (11 m) on shell, crust, Murchison NW (restoration site near Plot 1), British Columbia, Canada, 22.7.2023, L. Lee, D. Okamoto, L. Vigneault & A. Galloway |  |  |  | PQ888475 | PQ883062 |  |
| *Boreolithothamnion tanuense* | GWS049815 (Isotype) | Subtidal (10.4 m), rhodolith, Tanu I., British Columbia, Canada, 52.76233 -131.61822, 23.7.2023, L. Lee, D. Okamoto, L. Vigneault & A. Galloway |  |  |  | PQ888669 |  |  |
| *Boreolithothamnion tanuense* | GWS049824 (Isotype) | Subtidal (10.4 m), rhodolith, Tanu I., British Columbia, Canada, 52.76233 -131.61822, 23.7.2023, L. Lee, D. Okamoto, L. Vigneault & A. Galloway |  |  |  | PQ888704 |  |  |
| *Boreolithothamnion tanuense* | GWS049839 (Isotype) | Subtidal (10.4 m), rhodolith, Tanu I., British Columbia, Canada, 52.76233 -131.61822, 23.7.2023, L. Lee, D. Okamoto, L. Vigneault & A. Galloway |  |  |  | PQ888659 |  |  |
| *Boreolithothamnion tanuense* | GWS049842 (Isotype) | Subtidal (10.4 m), rhodolith, Tanu I., British Columbia, Canada, 52.76233 -131.61822, 23.7.2023, L. Lee, D. Okamoto, L. Vigneault & A. Galloway |  |  |  | PQ888503 |  |  |
| *Boreolithothamnion tanuense* | GWS049845 (Isotype) | Subtidal (10.4 m), rhodolith, Tanu I., British Columbia, Canada, 52.76233 -131.61822, 23.7.2023, L. Lee, D. Okamoto, L. Vigneault & A. Galloway |  | PQ883008 |  | PQ888719 |  |  |
| *Boreolithothamnion tanuense* | GWS049846 (Holotype) | Subtidal (10.4 m), rhodolith, Tanu I., British Columbia, Canada, 52.76233 -131.61822, 23.7.2023, L. Lee, D. Okamoto, L. Vigneault & A. Galloway |  | PQ883007 |  | PQ888715 |  |  |
| *Boreolithothamnion tanuense* | GWS049853 (Isotype) | Subtidal (10.4 m), rhodolith, Tanu I., British Columbia, Canada, 52.76233 -131.61822, 23.7.2023, L. Lee, D. Okamoto, L. Vigneault & A. Galloway |  |  |  | PQ888497 |  |  |
| *Boreolithothamnion tanuense* | GWS049860 | Subtidal (6.5 m), rhodolith, Stansung I., British Columbia, Canada, 52.73877 -131.61853, 23.7.2023, L. Lee, D. Okamoto, L. Vigneault & A. Galloway |  |  |  | PQ888703 |  |  |
| *Boreolithothamnion tophiforme* | GWS007314 | Subtidal (20 m) on rock, rhodolith, Maerl bed (btw Deer Arm & East Arm), Bonne Bay, Newfoundland and Labrador, Canada, 49.525278 -57.825556, 13.7.2006, L. Le Gall, B. Hooper & J. Utge | HQ919560 |  |  | PQ888698 |  |  |
| *Boreolithothamnion tophiforme* | GWS007315 | Subtidal (20 m) on rock, rhodolith, Maerl bed (btw Deer Arm & East Arm), Bonne Bay, Newfoundland and Labrador, Canada, 49.525278 -57.825556, 13.7.2006, L. Le Gall, B. Hooper & J. Utge | HM916900 |  | PQ882809 |  |  |  |
| *Boreolithothamnion tophiforme* | GWS007327 | Subtidal (20 m) on rock, crust, Maerl bed (btw Deer Arm & East Arm), Bonne Bay, Newfoundland and Labrador, Canada, 49.525278 -57.825556, 13.7.2006, L. Le Gall, B. Hooper & J. Utge |  | MW536875 |  |  | PQ883080 |  |
| *Bossiella frondifera* | GWS004365 | Low intertidal on rock, articulated, Botany Beach, near Port Renfrew, Vancouver I., British Columbia, Canada, 48.5304 -124.4535, 26.6.2006, G.W. Saunders, B. Clarkston & D. McDevit | KJ591909 |  | KJ592017 | KJ637849 | PQ137867 |  |
| *Bossiella heteroforma* | GWS022338 | Subtidal (16 m) on shale, Monterey Bay (shale bed), California, United States of America, 36.609 -121.879, 23.5.2010, B. Clarkston, K. Hind & S. Toews | HQ544243 |  | In progress |  |  |  |
| *Bossiella heteroforma* | GWS028231 | Subtidal (6 m) on rock, Haswell Bay (north point in bay), Gwaii Haanas, Haida Gwaii, British Columbia, Canada, 52.529 -131.607, 8.7.2011, G.W. Saunders & K. Dixon | KJ637745 | PQ883164 |  | PQ883178 | PQ883147 | PQ883172 |
| *Bossiella* sp. 1heteroforma | GWS046756 | Subtidal (7 m), articulated rhodolith, Channel btw Murchison & Faraday I., Gwaii Haanas, British Columbia, Canada, 52.59703 -131.47345, 27.7.2022, G.W. Saunders & C. Brooks |  | PQ883167 |  | PQ883180 | PQ883149 |  |
| *Bossiella* sp. 1heteroforma | GWS046829 | Subtidal (10 m) on limpet, articulated, Murchison I. NW site, Plot 2 (10-30 ft) Gwaii Haanas, British Columbia, Canada, 52.60913 -131.46582, 28.7.2022, G.W. Saunders & C. Brooks |  | PQ883160 |  |  |  |  |
| *Calliarthron tuberculosum* | GWS002904 | Low intertidal on rock pools in channel past Blowhole, articulated, Bamfield, Blowhole at Brady’s Beach, British Columbia, Canada, 48.8235 -125.1613, 8.6.2005, G.W. Saunders | PQ883022 |  | PQ883151 | PQ883173 | PQ883146 |  |
| *Chiharaea americana* f. *bodegensis* | GWS010828 | Low intertidal on rock, semi-exposed, articulated, Bamfield, Seppings I., British Columbia, Canada, 48.8391 -125.2075, 5.6.2008, K. Hind & D. McDevit | HM918942 |  | JQ677000 | PQ888728 | KC157588 | KC170016 |
| *Clathromorphum circumscriptum* | GWS018152 | Subtidal (2.5 m) on rock, crust, Two Lights, Cape Elizabeth, Maine, United States of America, 43.56481 -70.19865, 19.4.2010, B. Clarkston, D. McDevit, M. Bruce, A. Savoie, C. Longtin | HM915208 |  |  |  | PQ883074 |  |
| *Clathromorphum circumscriptum* | GWS032147 | Subtidal (2 m), crust, Gaulman Pt., Nova Scotia, Canada, 45.35468 -61.19365, 15.8.2012, G.W. Saunders & A. Savoie | MH308948 |  | MH277189 |  |  |  |
| *Clathromorphum circumscriptum* | GWS039518 | Low intertidal pool on rock, crust, Lepreau exposed biodiversity site, Bay of Fundy, New Brunswick, Canada, 45.0722 -66.469, 21.5.2015, G.W. Saunders & C. Brooks | PQ883047 |  |  | PQ888656 |  |  |
| *Clathromorphum* sp. 1compactum | GWS008855 | Subtidal (8 m) on quartz boulder, crust, SE of Beaver Harbour in SCUBA Bay, Bay of Fundy, New Brunswick, Canada, 45.0563 -66.7358, 12.9.2007, G.W. Saunders, K. Dixon & D. McDevit | MH309451 |  |  |  |  |  |
| *Clathromorphum* sp. 1compactum | GWS008857 | Subtidal (8 m) on quartz boulder, crust, SE of Beaver Harbour in SCUBA Bay, Bay of Fundy, New Brunswick, Canada, 45.0563 -66.7358, 12.9.2007, G.W. Saunders, K. Dixon & D. McDevit | MH309614 |  | MH277243 |  |  |  |
| *Clathromorphum* sp. 1compactum | GWS013861 | Subtidal (4 m) on rock, crust, Liberty Point, Campobello I., New Brunswick, Canada, 44.83088 -66.92767, 19.8.2009, G.W. Saunders, B. Clarkston & D. McDevit | HQ919251 |  |  | PQ888515 |  |  |
| *Clathromorphum* sp. 1compactum | GWS040286 | Subtidal (6 m) on cobble, crust, N Head of Evans Bight, Torngat, Labrador, Newfoundland and Labrador, Canada, 59.29849 -63.52443, 8.9.2014, G.W. Saunders, K. Dixon & C. Lane | MH309911 |  | In progress | PQ888716 | PQ883090 |  |
| *Corallina officinalis* | GWS000343 | Intertidal pool on rock, articulated, Maces Bay, Lepreau, Bay of Fundy, New Brunswick, Canada, 45.10276 -66.47863, 10.1.1998, G.W. Saunders | JQ615726 |  |  |  | AF419116 |  |
| *Corallina officinalis* | GWS002371 | Low intertidal on rock, articulated, Cape Saint Marys, Nova Scotia, Canada, 44.0871 -66.2034, 5.9.2004, G.W. Saunders | JQ615683 |  | KC134323 | KU501322 |  |  |
| *Crusticorallina painei* | GWS020983 | Subtidal (6 m) on rock, crust, Between Wiah Point & Cape Edenshaw (#2), NW of Masset, Haida Gwaii, British Columbia, Canada, 54.10699 -132.36641, 8.6.2010, G.W. Saunders & K. Dixon | HQ545224 |  | PQ882917 | JQ422241 | PQ883075 |  |
| *Crusticorallina painei* | GWS048513 | Subtidal (2 m) on limpet, crust, Murchison I. NE site, Plot 1 (2-5 ft), Gwaii Haanas, British Columbia, Canada, 52.61563 -131.43754, 1.8.2022, G.W. Saunders & C. Brooks |  | PQ882902 |  | PQ888578 |  |  |
| *Dawsoniolithon conicum* | GWS011968 | , crust, Imperieuse Reef east, Rowley Shoals, Western Australia, Australia, 5.12.2007, J. Huisman | PQ137863 |  | PQ149468 | PQ149491 | PQ137887 |  |
| Hapalidiales_unk1 sp. 1HG | GWS010040 | upper intertidal pool on rock, crust, Tahsis, I. #40 on Esperenza Inlet Chart, British Columbia, Canada, 49.8125 -126.98733, 21.5.2008, K. Hind & D. McDevit | PQ883041 |  |  |  |  |  |
| Hapalidiales_unk1 sp. 1HG | GWS045640 | Subtidal (6 m) on cobble, crust, Faraday I., S Site, Plot 2 (10-30 ft), Gwaii Haanas, British Columbia, Canada, 52.60913 -131.46582, 19.7.2019, G.W. Saunders & C. Brooks |  | PQ882852 |  |  | PQ883066 |  |
| Hapalidiales_unk1 sp. 1HG | GWS046976 | Subtidal (10 m) on rock, crust, Arnold Pt. (east side, left transects), Gwaii Haanas, British Columbia, Canada, 52.09895 -131.13159, 30.7.2022, G.W. Saunders & C. Brooks |  | PQ883001 |  | PQ888697 | PQ883088 |  |
| *Jania rosea* | GWS016456 | Subtidal (10 m) on inverts, articulated, Stanley Breakwater, Tasmania, Australia, -40.76731 145.305829, 29.1.2010, G.W. Saunders, L. Kraft & K. Dixon | HM918209 |  | MH777614 | MH777611 | PQ137878 |  |
| *Jania sagittata* | GWS016470 | Subtidal (10 m) on inverts, articulated, Stanley Breakwater, Tasmania, Australia, -40.76731 145.305829, 29.1.2010, G.W. Saunders, L. Kraft & K. Dixon | HM918212 |  | KC134331 | KP224288 | KC157591 |  |
| *Johansenia macmillanii* | GWS008123 | low intertidal, exposed, on rock, articulated, Lands End up the left side of Pachena Bay where it opens to the ocean, Bamfield, British Columbia, Canada, 48.7715 -125.1578, 2.6.2007, B. Clarkston, D. McDevit, K. Roy & H. Kucera | JQ615861 |  | MH777617 | MH777613 | PQ137876 |  |
| *Leptophytum foecundum* | GWS006292 | Subtidal (8 m) on mussel, crust, Meadow Cove exposed biodiversity site, Bay of Fundy, New Brunswick, Canada, 45.0381 -66.8913, 6.9.2007, G.W. Saunders | MH309848 |  | MH277264 | PQ888622 | PQ883081 |  |
| *Leptophytum laeve* | GWS008820 | Subtidal (30 m), crust, Bonne Bay, Newfoundland and Labrador, Canada, 49.3 57.52, 1.9.2007, B. Hooper | MH309473 |  | MH277233 | PQ888615 |  |  |
| Lithothamniac sp. 1JAcrust | GWS011959 | Low intertidal on reef flat rock, crust, Kikoni (site of old marine station), Japan, 41.70075 140.52399, 2.12.2008, G.W. Saunders, T. Abe & N. Yotsukura | HM918981 |  | PQ882897 | PQ888573 | PQ883072 |  |
| *Mastophora rosea* | PAGO0003B | Intertidal, crust, Pago Bay, in front of UOGML, Guam, United States of America, 5.4.2007, T. Schils | PQ137855 |  | PQ149458 | KP224287 | KP224296 |  |
| *Mastophoropsis canaliculata* | GWS015547 | Subtidal (1-2 m) on rock, non-geniculate frond, Nine Pin Point, Tasmania, Australia, -43.28649 147.16429, 22.1.2010, G.T. Kraft & L. Kraft | HM917843 |  | KC134335 | KU501331 | KC157592 |  |
| *Mesophyllum conchatum* | GWS019584 | Subtidal (8 m) on articulated coralline, crust, Ucluelet, Sargison Bank, British Columbia, Canada, 48.92205 -125.42069, 2.6.2010, G.W. Saunders & K. Dixon | HQ544509 |  | PQ882890 | PQ888566 | PQ883071 |  |
| *Mesophyllum conchatum* | GWS047735 | Subtidal (2 m) on articulated coralline, crust, Murchison I. NW site, Plot 2 (2-5 ft) Gwaii Haanas, British Columbia, Canada, 52.61456 -131.44443, 5.9.2021, G.W. Saunders & C. Brooks |  |  | PQ883012 |  | PQ883091 |  |
| *Mesophyllum conchatum* | GWS048049 | Subtidal (1.5 m) on articulated coralline (GWS048048), crust, Murchison I. NW site, Plot 1 (2-5 ft), Gwaii Haanas, British Columbia, Canada, 52.61456 -131.44443, 8.9.2021, G.W. Saunders & C. Brooks | PQ883036 |  |  |  |  |  |
| *Mesophyllum* sp. 1erubescens | GWS016134 | Subtidal (13 m) on shell, crust, Lucas Point (past Hells Gates) Strahan, Tasmania, Australia, -42.1997 145.2008, 26.1.2010, G.W. Saunders & K. Dixon | HM918061 |  | PQ882930 | PQ888597 | PQ883077 |  |
| *Mesophyllum* sp. 1TAScrust | GWS015501 | Subtidal (14 m) on invert, crust, Reef west of Verona Sands boat launch, in mouth of Huon River, Tasmania, Australia, -43.275 147.1257, 22.1.2010, G.W. Saunders & K. Dixon | HM917814 |  | PQ882815 |  | PQ883063 |  |
| *Mesophyllum* sp. 1TAScrust | GWS016548 | Subtidal (5 m) on rock, crust, Stanley Breakwater, Tasmania, Australia, -40.76731 145.30583, 29.1.2010, G.W. Saunders, L. Kraft & K. Dixon | PQ883031 |  | PQ882858 | PQ888524 | PQ883067 |  |
| *Mesophyllum* sp. 2erubescens | GWS015057 | Subtidal (23 m) on cobble, crust, Windmill Point, George Town, Tasmania, Australia, -41.1097 146.81699, 19.1.2010, G.W. Saunders & K. Dixon | HM917561 |  | PQ882956 | PQ888636 | PQ883082 |  |
| *Mesophyllum* sp. 2TAScrust | GWS015371 | Subtidal (13 m) on rock, crust, George III Reef, Tasmania, Australia, -43.50742 146.98469, 21.1.2010, F. Scott & C. Sanderson | HM917728 |  |  | PQ888451 | PQ883060 |  |
| *Mesophyllum vancouveriense* | GWS010090 | Subtidal (20 m) on rock, crust, Tahsis, I. south of Clotchman I., Spanish Pilot Group, Tahsis, British Columbia, Canada, 49.61454 -126.58255, 23.5.2008, K. Hind & D. McDevit | HM918925 |  | KC134326 | KP224289 | KC157589 |  |
| *Metagoniolithon stelliferum* | GWS016595 | Subtidal (5 m) on seagrass, articulated, The Springs, Point Lonsdale, Victoria, Australia, -38.27605 144.62029, 2.2.2010, G.W. Saunders, L. Kraft & K. Dixon | HM918262 |  | PQ149465 | PQ149488 | PQ137884 |  |
| *Neopolyporolithon reclinatum* | GWS008332 | mid intertidal pool on Corallina, crust, Ridley I. (south of coal terminal), Prince Rupert, British Columbia, Canada, 54.2212 -130.3293, 8.6.2007, G.W. Saunders, B. Clarkston, D. McDevit & K. Roy | KC130140 |  | KC134324 | KU501323 | KC157587 |  |
| *Neopolyporolithon* sp. 3GWS | GWS021621 | Low intertidal on 21620, crust, Soberanes Point, California, United States of America, 36.44786 -121.92879, 17.5.2010, B. Clarkston, K. Hind & S. Toews | KM254608 |  | PQ882785 | PQ888435 | PQ883056 |  |
| *Phymatolithon laevigatum* | GWS017972 | Subtidal (3 m) on glass, crust, East Point Beach, Groton, Connecticut, United States of America, 41.32002 -72.07483, 15.4.2010, B. Clarkston, D. McDevit, M. Bruce, A. Savoie, C. Longtin | HM915148 |  |  |  |  |  |
| *Phymatolithon laevigatum* | GWS017977 | Subtidal (3 m) on glass, crust, East Point Beach, Groton, Connecticut, United States of America, 41.32002 -72.07483, 15.4.2010, B. Clarkston, D. McDevit, M. Bruce, A. Savoie, C. Longtin | PQ883028 |  | PQ882849 | PQ888510 |  |  |
| *Phymatolithon laevigatum* | GWS017982 | Subtidal (3 m) on glass, crust, East Point Beach, Groton, Connecticut, United States of America, 41.32002 -72.07483, 15.4.2010, B. Clarkston, D. McDevit, M. Bruce, A. Savoie, C. Longtin |  | PQ882974 |  |  | PQ883085 |  |
| *Phymatolithon lenormandii* | GWS018082 | Subtidal (1.5 m) on rock, crust, Fort Wetherill, Rhode I., United States of America, 41.4791 -71.360656, 16.4.2010, B. Clarkston, D. McDevit, M. Bruce, A. Savoie, C. Longtin | HM915182 |  | MN184553 | PQ888474 | PQ883061 |  |
| *Phymatolithon masonianum* | GWS015345 | Subtidal (6 m) on rock, crust, Burying Ground Point, Tasmania, Australia, -43.44013 146.98883, 21.1.2010, G.W. Saunders & K. Dixon | HM917708 |  | PQ149454 | PQ149477 | PQ137872 |  |
| *Phymatolithon scabriusculum* | GWS011762 | Subtidal (10 m) on rock, crust, Flat Wolf I., New Brunswick, Canada, 44.95447 -66.73139, 21.8.2008, K. Hind & D. McDevit | HM918967 |  | PQ882885 | PQ888560 |  |  |
| *Phymatolithon scabriusculum* | GWS013995 | Subtidal (9 m) on rock, crust, Meadow Cove exposed biodiversity site, Bay of Fundy, New Brunswick, Canada, 45.0381 -66.8913, 25.8.2010, G.W. Saunders, M. Bruce, A. Savoie & K. Dixon | PQ883054 |  |  | PQ888713 | PQ883089 |  |
| *Phymatolithon* sp. 1Cal | GWS022120 | Subtidal (5 m) on rock, crust, Stillwater Cove, Pebble Beach, California, United States of America, 36.566674 -121.94289, 20.5.2010, B. Clarkston, K. Hind & S. Toews | HQ544198 |  | PQ882961 | PQ888644 | PQ883084 |  |
| *Phymatolithon* sp. 6ATcrust | GWS008908 | Subtidal (10 m) on boulder, crust, Meadow Cove exposed biodiversity site, Bay of Fundy, New Brunswick, Canada, 45.0381 -66.8913, 6.9.2007, K. Dixon | PQ883030 |  | PQ882857 | PQ888522 |  |  |
| *Phymatolithon* sp. 6ATcrust | GWS048714 | Subtidal (6 m) on rock, crust, Mispec Beach (Rocks north 2), Saint John Harbour monitoring site, New Brunswick, Canada, 45.21418 -65.97055, 12.8.2021, G.W. Saunders & C. Brooks |  | PQ882873 |  |  | PQ883069 |  |
| *Rhodolithia gracilis* var. *gracilis* | GWS009188 (Holotype) | Subtidal (10 m) on rock, crust, Gilbert I., Broken Group, British Columbia, Canada, 48.87852 -125.32713, 22.9.2007, G.W. Saunders & B. Clarkston | HM918901 |  | PQ882926 | PQ888593 | PQ883076 | PQ883120 |
| *Rhodolithia gracilis* var. *gracilis* | GWS009190 | Subtidal (11 m) on rock, crust, Bamfield, Wizard I., British Columbia, Canada, 48.8583 -125.1588, 23.9.2007, G.W. Saunders & B. Clarkston | HM918902 |  | PQ882900 | PQ888576 | PQ883073 | PQ883117 |
| *Rhodolithia gracilis* var. *gracilis* | GWS009505 | Subtidal (16 m) on rock. crust, Pipers Point, Sechelt, British Columbia, Canada, 49.54668 -123.79256, 16.5.2008, G.W. Saunders, B. Clarkston, D. McDevit & K. Hind | PQ883032 |  |  | PQ888528 |  | PQ883105 |
| *Rhodolithia gracilis* var. *gracilis* | GWS009559 | Subtidal (16 m) on rock. crust, Powerline dive site, near Sechelt, British Columbia, Canada, 49.648861 -123.837694, 17.5.2008, G.W. Saunders, B. Clarkston, D. McDevit & K. Hind | PQ883026 |  |  | PQ888481 |  |  |
| *Rhodolithia gracilis* var. *gracilis* | GWS019524 | Subtidal (14 m) on rock; dredge (site 1), crust, Bamfield, off Wizard, British Columbia, Canada, 48.8571 -125.1635, 1.6.2010, N. Jeffery & O. Pierossi | HQ544459 |  | PQ882842 | PQ888501 | PQ883065 | PQ883100 |
| *Rhodolithia gracilis* var. *gracilis* | GWS019647 | Subtidal (10 m) on rock, crust, Bamfield, opening of Grappler Inlet, British Columbia, Canada, 48.8377 -125.1339, 3.6.2010, G.W. Saunders & K. Dixon | PQ883035 |  |  | PQ888542 |  | PQ883110 |
| *Rhodolithia gracilis* var. *gracilis* | GWS019652 | Subtidal (10 m) on rock, crust, Bamfield, opening of Grappler Inlet, British Columbia, Canada, 48.8377 -125.1339, 3.6.2010, G.W. Saunders & K. Dixon | HQ919316 |  |  | PQ888548 |  | PQ883111 |
| *Rhodolithia gracilis* var. *gracilis* | GWS030831 | Subtidal (8 m) on cobble, crust, Macrocystis bed at entrance to George Bay, Gwaii Haanas, British Columbia, Canada, 52.30665 -131.33208, 11.6.2012, G.W. Saunders & K. Dixon | PQ883052 | PQ883003 |  | PQ888702 |  | PQ883131 |
| *Rhodolithia gracilis* var. *gracilis* | GWS035013 | Subtidal (15 m) on rock, crust, Worlcombe Isl. (east point), British Columbia, Canada, 49.34837 -123.45287, 17.5.2013, G.W. Saunders & K. Dixon | PQ883040 |  |  | PQ888574 |  | PQ883116 |
| *Rhodolithia gracilis* var. *gracilis* | GWS045566 | Subtidal (7 m) on rock, crust, Faraday I., S Site, Plot 1 (10-30 ft), Gwaii Haanas, British Columbia, Canada, 52.60913 -131.46582, 19.7.2019, G.W. Saunders & C. Brooks | PQ883055 | PQ883011 |  | PQ888724 |  | PQ883134 |
| *Rhodolithia gracilis* var. *gracilis* x *ramosa* | GWS028207 (Holotype) | Subtidal (22 m) (anchor dredge), rhodolith, Hotspring I. (back side in channel), Gwaii Haanas, British Columbia, Canada, 52.5779 -131.43768, 7.7.2011, G.W. Saunders & K. Dixon | PQ883049 | PQ882979 |  | PQ888667 |  | PQ883127 |
| *Rhodolithia gracilis* var. *gracilis* x *ramosa* | GWS031003 | Subtidal (10 m), rhodolith, Tanu I., Gwaii Haanas, British Columbia, Canada, 52.76458 -131.61066, 14.6.2012, G.W. Saunders & K. Dixon | PQ883027 | PQ882848 |  | PQ888506 |  | PQ883101 |
| *Rhodolithia gracilis* var. *gracilis* x *ramosa* | GWS031004 | Subtidal (10 m), rhodolith, Tanu I., Gwaii Haanas, British Columbia, Canada, 52.76458 -131.61066, 14.6.2012, G.W. Saunders & K. Dixon | PQ883033 |  | PQ882863 | PQ888535 | PQ883068 | PQ883108 |
| *Rhodolithia gracilis* var. *gracilis* x *ramosa* | GWS046927 | Subtidal (20 m), rhodolith, Nereo Pinnacle, Faraday Pass east side, Gwaii Haanas, British Columbia, Canada, 52.6199 -131.4601, 29.7.2022, G.W. Saunders & C. Brooks |  |  |  | PQ888529 |  | PQ883107 |
| *Rhodolithia gracilis* var. *gracilis* x *ramosa* | GWS046928 | Subtidal (20 m), rhodolith, Nereo Pinnacle, Faraday Pass east side, Gwaii Haanas, British Columbia, Canada, 52.6199 -131.4601, 29.7.2022, G.W. Saunders & C. Brooks | PQ883025 | PQ882811 |  | PQ888471 |  | PQ883098 |
| *Rhodolithia gracilis* var. *gracilis* x *ramosa* | GWS046929 | Subtidal (20 m), rhodolith, Nereo Pinnacle, Faraday Pass east side, Gwaii Haanas, British Columbia, Canada, 52.6199 -131.4601, 29.7.2022, G.W. Saunders & C. Brooks |  |  |  | PQ888460 |  | PQ883096 |
| *Rhodolithia gracilis* var. *gracilis* x *ramosa* | GWS046930 | Subtidal (20 m), rhodolith, Nereo Pinnacle, Faraday Pass east side, Gwaii Haanas, British Columbia, Canada, 52.6199 -131.4601, 29.7.2022, G.W. Saunders & C. Brooks | PQ883051 | PQ883000 |  | PQ888695 |  | PQ883130 |
| *Rhodolithia gracilis* var. *gracilis* x *ramosa* | GWS046931 | Subtidal (20 m), rhodolith, Nereo Pinnacle, Faraday Pass east side, Gwaii Haanas, British Columbia, Canada, 52.6199 -131.4601, 29.7.2022, G.W. Saunders & C. Brooks | PQ883034 |  |  | PQ888541 |  | PQ883109 |
| *Rhodolithia gracilis* var. *gracilis* x *ramosa* | GWS046932 | Subtidal (20 m), rhodolith, Nereo Pinnacle, Faraday Pass east side, Gwaii Haanas, British Columbia, Canada, 52.6199 -131.4601, 29.7.2022, G.W. Saunders & C. Brooks | PQ883048 |  | PQ882976 | PQ888662 | PQ883086 | PQ883125 |
| *Rhodolithia gracilis* var. *ramosa* | GWS010257 (Holotype) | Subtidal (13 m), rhodolith, Tahsis, Princesa Channel, British Columbia, Canada, 49.724722 -126.6425, 25.5.2008, G.W. Saunders & B. Clarkston | PQ883024 |  |  | PQ888458 |  | PQ883095 |
| *Rhodolithia gracilis* var. *ramosa* | GWS010269 (Isotype) | Subtidal (8 m), rhodolith, Tahsis, Princesa Channel, British Columbia, Canada, 49.724722 -126.6425, 25.5.2008, K. Hind & D. McDevit | PQ883053 | PQ883004 |  | PQ888706 |  | PQ883132 |
| *Rhodolithia gracilis* var. *ramosa* | GWS010270 (Isotype) | Subtidal (8 m), rhodolith, Tahsis, Princesa Channel, British Columbia, Canada, 49.724722 -126.6425, 25.5.2008, K. Hind & D. McDevit | PQ883037 |  |  | PQ888551 |  | PQ883112 |
| *Roseolithon* sp. 3Rhodolith | GWS018578 | Subtidal (10 m), rhodolith, Rocky Reef at Lighthouse 'Point' Piyangdo I., Jeju, South Korea, 33.4198 126.2244, 20.5.2010, G.W. Saunders & H-G. Choi | HQ544124 |  | PQ882826 | PQ888484 | PQ883064 |  |
| *Titanoderma macrocarpum* | GWS017809 | Subtidal (8 m) on Chondrus, crust, Folly Cove, Gloucester left side, Massachusetts, United States of America, 42.68502 -70.64148, 13.4.2010, D. McDevit, M. Bruce | HM91507 |  | PQ882787 |  | PQ883057 |  |
| *Titanoderma macrocarpum* | GWS018174 | Subtidal (5 m) on Chondrus, crust, Two Lights, Cape Elizabeth, Maine, United States of America, 43.56481 -70.19865, 19.4.2010, B. Clarkston, D. McDevit, M. Bruce, A. Savoie, C. Longtin | PQ883039 |  |  | PQ888563 |  |  |
| Rhodolith sp. | GWS009194 | Subtidal (11 m) on rock, rhodolith, Bamfield, Wizard I., British Columbia, Canada, 48.8583 -125.1588, 23.9.2007, G.W. Saunders & B. Clarkston |  |  |  |  |  |  |
| Rhodolith sp. | GWS010611 | Subtidal (13 m), rhodolith, Bamfield, Wizard I., British Columbia, Canada, 48.8583 -125.1588, 3.6.2008, B. Clarkston & S. Toews |  |  |  |  |  |  |
| Rhodolith sp. | GWS028058 | Subtidal (8 m), rhodolith, Channel btw Murchison & Faraday I., Gwaii Haanas, British Columbia, Canada, 52.59687 -131.47513, 7.7.2011, G.W. Saunders & K. Dixon |  |  |  |  |  |  |
| Rhodolith sp. | GWS028071 | Subtidal (5 m), rhodolith, Channel btw Murchison & Faraday I., Gwaii Haanas, British Columbia, Canada, 52.59687 -131.47513, 7.7.2011, G.W. Saunders & K. Dixon |  |  |  |  |  |  |
| Rhodolith sp. | GWS046700 | Subtidal (7 m), rhodolith, Channel btw Murchison & Faraday I., Gwaii Haanas, British Columbia, Canada, 52.59703 -131.47345, 27.7.2022, G.W. Saunders & C. Brooks |  |  |  |  |  |  |
| Rhodolith sp. | GWS046702 | Subtidal (7 m), rhodolith, Channel btw Murchison & Faraday I., Gwaii Haanas, British Columbia, Canada, 52.59703 -131.47345, 27.7.2022, G.W. Saunders & C. Brooks |  |  |  |  |  |  |
| Rhodolith sp. | GWS046704 | Subtidal (7 m), rhodolith, Channel btw Murchison & Faraday I., Gwaii Haanas, British Columbia, Canada, 52.59703 -131.47345, 27.7.2022, G.W. Saunders & C. Brooks |  |  |  |  |  |  |
| Rhodolith sp. | GWS046709 | Subtidal (7 m), rhodolith, Channel btw Murchison & Faraday I., Gwaii Haanas, British Columbia, Canada, 52.59703 -131.47345, 27.7.2022, G.W. Saunders & C. Brooks |  |  |  |  |  |  |
| Rhodolith sp. | GWS046711 | Subtidal (7 m), rhodolith, Channel btw Murchison & Faraday I., Gwaii Haanas, British Columbia, Canada, 52.59703 -131.47345, 27.7.2022, G.W. Saunders & C. Brooks |  |  |  |  |  |  |
| Rhodolith sp. | GWS046722 | Subtidal (7 m), rhodolith, Channel btw Murchison & Faraday I., Gwaii Haanas, British Columbia, Canada, 52.59703 -131.47345, 27.7.2022, G.W. Saunders & C. Brooks |  |  |  |  |  |  |
| Rhodolith sp. | GWS046727 | Subtidal (7 m), rhodolith, Channel btw Murchison & Faraday I., Gwaii Haanas, British Columbia, Canada, 52.59703 -131.47345, 27.7.2022, G.W. Saunders & C. Brooks |  |  |  |  |  |  |
| Rhodolith sp. | GWS046732 | Subtidal (7 m), rhodolith, Channel btw Murchison & Faraday I., Gwaii Haanas, British Columbia, Canada, 52.59703 -131.47345, 27.7.2022, G.W. Saunders & C. Brooks |  |  |  |  |  |  |
| Rhodolith sp. | GWS046738 | Subtidal (7 m), rhodolith, Channel btw Murchison & Faraday I., Gwaii Haanas, British Columbia, Canada, 52.59703 -131.47345, 27.7.2022, G.W. Saunders & C. Brooks |  |  |  |  |  |  |
| Rhodolith sp. | GWS046743 | Subtidal (7 m), rhodolith, Channel btw Murchison & Faraday I., Gwaii Haanas, British Columbia, Canada, 52.59703 -131.47345, 27.7.2022, G.W. Saunders & C. Brooks |  |  |  |  |  |  |
| Rhodolith sp. | GWS046744 | Subtidal (7 m), rhodolith, Channel btw Murchison & Faraday I., Gwaii Haanas, British Columbia, Canada, 52.59703 -131.47345, 27.7.2022, G.W. Saunders & C. Brooks |  |  |  |  |  |  |
| Rhodolith sp. | GWS046903 | Subtidal (6 m), rhodolith, Murchison I. anchorage east entrance, Gwaii Haanas, British Columbia, Canada, 52.595183 -131.464537, 28.7.2022, G.W. Saunders & C. Brooks |  |  |  |  |  |  |
| Rhodolith sp. | GWS046905 | Subtidal (6 m), rhodolith, Murchison I. anchorage east entrance, Gwaii Haanas, British Columbia, Canada, 52.595183 -131.464537, 28.7.2022, G.W. Saunders & C. Brooks |  |  |  |  |  |  |
| Rhodolith sp. | GWS046907 | Subtidal (6 m), rhodolith, Murchison I. anchorage east entrance, Gwaii Haanas, British Columbia, Canada, 52.595183 -131.464537, 28.7.2022, G.W. Saunders & C. Brooks |  |  |  |  |  |  |
| Rhodolith sp. | GWS048565 | Subtidal (5 m), rhodolith, Channel btw Murchison & Faraday I., Gwaii Haanas, British Columbia, Canada, 52.59687 -131.47513, 2.8.2022, G.W. Saunders & C. Brooks |  |  |  |  |  |  |
| Rhodolith sp. | GWS048574 | Subtidal (5 m), rhodolith, Channel btw Murchison & Faraday I., Gwaii Haanas, British Columbia, Canada, 52.59687 -131.47513, 2.8.2022, G.W. Saunders & C. Brooks |  |  |  |  |  |  |
| Rhodolith sp. | GWS048578 | Subtidal (5 m), rhodolith, Channel btw Murchison & Faraday I., Gwaii Haanas, British Columbia, Canada, 52.59687 -131.47513, 2.8.2022, G.W. Saunders & C. Brooks |  |  |  |  |  |  |
| Rhodolith sp. | GWS048587 | Subtidal (5 m), rhodolith, Channel btw Murchison & Faraday I., Gwaii Haanas, British Columbia, Canada, 52.59687 -131.47513, 2.8.2022, G.W. Saunders & C. Brooks |  |  |  |  |  |  |
| Rhodolith sp. | GWS048595 | Subtidal (5 m), rhodolith, Channel btw Murchison & Faraday I., Gwaii Haanas, British Columbia, Canada, 52.59687 -131.47513, 2.8.2022, G.W. Saunders & C. Brooks |  |  |  |  |  |  |
| Rhodolith sp. | GWS048605 | Subtidal (5 m), rhodolith, Channel btw Murchison & Faraday I., Gwaii Haanas, British Columbia, Canada, 52.59687 -131.47513, 2.8.2022, G.W. Saunders & C. Brooks |  |  |  |  |  |  |
| Rhodolith sp. | GWS048612 | Subtidal (5 m), rhodolith, Channel btw Murchison & Faraday I., Gwaii Haanas, British Columbia, Canada, 52.59687 -131.47513, 2.8.2022, G.W. Saunders & C. Brooks |  |  |  |  |  |  |
| Rhodolith sp. | GWS048622 | Subtidal (5 m), rhodolith, Channel btw Murchison & Faraday I., Gwaii Haanas, British Columbia, Canada, 52.59687 -131.47513, 2.8.2022, G.W. Saunders & C. Brooks |  |  |  |  |  |  |
| Rhodolith sp. | GWS049713 | Subtidal (6.5 m), rhodolith, Hotspring I., British Columbia, Canada, 52.57847 -131.44319, 15.7.2023, L. Lee, D. Okamoto, L. Vigneault & A. Galloway |  |  |  |  |  |  |
| Rhodolith sp. | GWS049717 | Subtidal (6.5 m), rhodolith, Hotspring I., British Columbia, Canada, 52.57847 -131.44319, 15.7.2023, L. Lee, D. Okamoto, L. Vigneault & A. Galloway |  |  |  |  |  |  |
| Rhodolith sp. | GWS049718 | Subtidal (6.5 m), rhodolith, Hotspring I., British Columbia, Canada, 52.57847 -131.44319, 15.7.2023, L. Lee, D. Okamoto, L. Vigneault & A. Galloway |  |  |  |  |  |  |
| Rhodolith sp. | GWS049778 | Subtidal (4.6 m), rhodolith, Hotspring I., British Columbia, Canada, 52.57847 -131.44319, 15.7.2023, L. Lee, D. Okamoto, L. Vigneault & A. Galloway |  |  |  |  |  |  |
| Rhodolith sp. | GWS049806 | Subtidal (11 m), rhodolith, Murchison NW (restoration site near Plot 1), British Columbia, Canada, 22.7.2023, L. Lee, D. Okamoto, L. Vigneault & A. Galloway |  |  |  |  |  |  |
| Rhodolith sp. | GWS049836 | Subtidal (10.4 m), rhodolith, Tanu I., British Columbia, Canada, 52.76233 -131.61822, 23.7.2023, L. Lee, D. Okamoto, L. Vigneault & A. Galloway |  |  |  |  |  |  |
| Rhodolith sp. | GWS049854 | Subtidal (10.4 m), rhodolith, Tanu I., British Columbia, Canada, 52.76233 -131.61822, 23.7.2023, L. Lee, D. Okamoto, L. Vigneault & A. Galloway |  |  |  |  |  |  |
