# Supplementary material for: Assessment of rhodolith‐forming species diversity in British Columbia uncovers novel cryptic diversity in the genera Boreolithothamnion and Rhodolithia gen. nov. (Florideophyceae, Rhodophyta) and the occurrence of hybrid rhodoliths
Source: J Phycol. 2025 Aug 13;61(5):1371–93. doi: 10.1111/jpy.70066 (PMC12547645; doi:10.1111/jpy.70066)
Supplement: Supplementary file 2 — Table S2. Intraspecific and interspecific variation in the psbA (~950 bp) gene region for rhodolith‐forming and crustose genetic groups of Boreolithothamnion and Rhodolithia gen. nov., where n is the number of sequences. [file JPY-61-1371-s001.docx]

**Table S2.**

|  |  | *psb*A |  |  |
| --- | --- | --- | --- | --- |
| Species | *n* | Max intraspecific variation (%) | Nearest neighbor | Distance to nearest neighbor (%) |
| *Boreolithothamnion* |  |  |  |  |
| *B.* *astragaloi* | 94 | 0 | *B.* *soriferum* | 0.52 |
| *B. glaciale*^a^ | 2 | 0 | *B.* sp. 1glaciale | 0 |
| *B. phymatodeum* | 71 | 0.44 | *B.* *tanuense* | 0.4 |
| *B. soriferum* | 12 | 0 | *B.* *astragaloi* | 0.52 |
| *B.* sp. 1glaciale | 2 | 0 | *B.* sp. 1heterocladum | 0 |
| *B.* sp. 1heterocladum | 58 | 0.22 | *B.* sp. 1glaciale | 0 |
| *B. tanuense* | 13 | 0 | *B. phymatodeum* | 0.4 |
| *Rhodolithia* |  |  |  |  |
| *R.* *gracilis* var. *gracilis*^b^ | 15 | 0.21 | *R.* *gracilis* var. *ramosa* | 0.42 |
| *R.* *gracilis* var. *ramosa*^c^ | 7 | 0 | *R.* *gracilis* var. *gracilis* | 0.42 |

^a^GWS007542 excluded from analyses owing to ambiguities in the *psb*A sequence.

^b^Hybrid specimens with the *psb*A sequence of *R.* *gracilis* var. *gracilis* were included in these analyses.

^c^Hybrid specimens with the *psb*A sequence of *R.* *gracilis* var. *ramosa* were included in these analyses.
